# Supplementary material for: Sequence-structure-function relationships in the microbial protein universe
Source: Nat Commun. 2023 Apr 26;14:2351. doi: 10.1038/s41467-023-37896-w (PMC10133388; doi:10.1038/s41467-023-37896-w)
Supplement: Supplementary file 1 — Supplementary Information [file 41467_2023_37896_MOESM1_ESM.pdf]

Sequence-structure-function relationships in the microbial protein universe

Supplementary Information

Table of Contents

Supplementary Note 1: How to search the MIP database ... 3

Supplementary Methods ... 3

1. MIP dataset construction ... 3
2. Protein structure predictions ... 3
3. Function prediction ... 8

Supplementary Note 2: MIP dataset descriptions ... 11

1. MIP dataset curation ... 12
2. Sequence length distribution ... 13
3. Pairwise structural comparisons in the curated dataset via CATH ... 14

Supplementary Note 3: Model quality assessment on the MIP_random5000 dataset ... 15

1. Explanation of variables ... 15
2. DMPFold models have higher coil content than Rosetta models ... 18
3. Model quality is independent of sequence length and correlates with agreement between Rosetta and DMPFold models ... 19
4. Deeper Multiple Sequence Alignment leads to higher model quality ... 20
5. Transmembrane spans ... 21
6. Disorder ... 22
7. Model complexity ... 23
8. TM-score to CATH ... 24
9. Rosetta score ... 25

Supplementary Note 4: Additional results on the MIP_curated dataset ... 27

1. Transmembrane spans ... 27
2. Contact order ... 27
3. Disorder ... 28

Supplementary Note 5: Novel folds ... 28

1. Novel fold identification: overall procedure ... 28
2. Novel fold identification: verifying a sensible TM-score cutoff ... 28

3. Novel fold identification: comparison against CATH and PDB90 29
4. Clustering procedure to identify novel fold clusters 31
5. AlphaFold2 verification of putative novel folds 33
6. Novel fold examples 44
7. Largest novel fold clusters 48
8. Functions of the novel fold proteins 49
Supplementary Note 6: Protein universe visualization 50
1. Protein features mapped onto the protein universe visualization 50
2. Sample structures 53
3. Caveat 55
Supplementary Note 7: Sanity checks for sequence-structure-function relationships 56
1. Sequence-structure comparison 58
2. Sequence-function comparison 59
3. Structure-function comparison 60
Supplementary Note 8: Structure Function Motifs 61
1. Functional diversity of proteins with the same structure 61
2. Structural diversity of proteins with the same function 63
3. Carbohydrate binding (MF GO:0030246) - Fig. 4a 63
4. Maintenance of CRISPR repeat elements (BP GO:0043571) - Fig. 4b 64
5. Sole sub-class for lyases that do not belong in the other subclasses (EC 4.99.1.-) - Fig. 4c 65
Supplementary References 67

Supplementary Note 1: How to search the MIP database

Please see the Data availability statement on how to access MIP data. The Zenodo folder includes workflows and scripts to search the database using a query sequence, a query structure, or a query function, to find similar proteins in the MIP dataset.

Supplementary Methods

1. MIP dataset construction

The MIP dataset is constructed on the basis of GEBA1003 representative bacterial and archeal genomes from across the tree of life[1]. The dataset includes environmental samples from soil, ocean water, human gut microbiome and was designed to sample the microbial tree of life evenly. For each genome, we generated a list of predicted genes using Prodigal[2].

The raw gene catalog was processed using an incremental clustering approach, similar to the one employed by UniClust[3]. First, redundancy in the dataset was removed by using `linclust` (ie. clustering at 100% sequence identity), as implemented in MMSeqs2[4,5]. Then, the dataset was further clustered into 90%, 70% and 30% sequence identity clusters, with the last step (70% to 30% clustering) executed using the MMSeqs2 `clust` module.

The resulting dataset was sorted according to sequence length, sampling the entirety of sequences between 40 and 200 residues.

2. Protein structure predictions

1. Sequence alignments

Sequences for predictions were selected following the use of the microprot pipeline (https://github.com/biocore/microprot). Briefly, for each gene in each of the selected genomes the pipeline runs HHsearch[6] to discard fragments (domains) with detectable similarity to known structures (PDB70 database).

```
% hhsearch -i seq.fasta -d pdb70 -e 0.1 -Z 2000 -B 2000 -o seq.out -
oa3m seq.a3m

% python split_search.py seq.out seq.fasta -e 0.1 -l 40 -o
seq_cm.fasta
```

Then, each fragment is split further by running HHsearch against Pfam.

```
% hhsearch -i seq_cm.fasta -d pfamA -e 0.1 -Z 2000 -B 2000 -o
seq_cm.out -oa3m seq_cm.a3m

% python split_search.py seq_cm.out seq_cm.fasta -e 0.01 -p 90.0 -l
40 -o seq_pfam.fasta
```

Each putative domain is assessed as being suitable for *ab initio* predictions or not on the basis on the number of effective sequences in an alignment (Neff) following HHblits.

```
% hhblits -i seq_pfam.fasta -d UniRef30 -n 3 -e 0.01 -diff inf -cov
50 -Z 10000 -B 10000 -o seq_pfam.out -oa3m seq_pfam.a3m

% python calculate_Neff.py -i seq_pfam.a3m -c 80 -o seq_msa.neff
```

Within the lifetime of the Microbiome Immunity Project (Aug 2017 - Jun 2021) reference databases were consistently updated to reflect up-to-date changes.

2. Fragment picking

Rosetta's *ab initio* structure prediction protocols use a fragment assembly approach of 3 to 9 residue peptide fragments extracted from experimentally determined structures in the PDB and that have similar sequences and/or predicted properties to the target sequence. Protein fragments capture local structure and can speed up the search of conformation space of the target sequence being predicted. We created fragment libraries using the standard protocol[7]. The fragment picking protocol makes use of NCBI BLAST[8] (version 2.2.17) and the NCBI Non-Redundant database (downloaded Aug 2017) for alignments and sequence profiles, SPARKS-X[9] and PSI-PRED[10] (version 3.3) for secondary structure prediction and Rosetta (version ac6fa1beb57c70914b419b2e07450f1add8b81f4) for picking fragments. For each of the MIP sequences, 200 fragments of length 3 and 9 residue were generated per position. The fragment picking tools are part of the standard Rosetta distribution package and require only the target sequence. Fragments are generated using a command like below.

```
% perl make_fragments.pl MIP_${KEY}.fasta
```

3. Residue-residue contact predictions

The accuracy of protein structure prediction can be improved by incorporating restraints from residue-pair interactions into the structure prediction trajectory, which are inferred from evolutionary couplings. We inferred residue-residue contacts from sequences closely-related to the target sequence using GREMLIN[11] (version 2.0.1). GREMLIN takes as input a target sequence and a multiple sequence alignment (described above) and outputs scores representative of the likelihood of a contact for each residue-pair in the target sequence. Multiple

sequence alignments were filtered using hhfilter, converted to PSICOV format[12] using the HH Suite reformat script, and then GREMLIN was run with the default values using the command lines below.

```
% hhfilter -id 90 -cov 75 -i ${A3M_PATH} -o MIP_${KEY}.a3m

% perl reformat.pl a3m psi MIP_${KEY}.a3m MIP_${KEY}.psi -l 10000 -M
first -r

% run_gremlin.sh /PATH/TO/MATLAB_Runtime/v717/ MIP_${KEY}.psi
MIP_${KEY}.mat
```

The raw GREMLIN score matrix was converted into a format that can be used in Rosetta: Raw scores between residue-pairs with a sequence separation of less than 3 were removed. Raw scores from all remaining residue-pairs in the score matrix were averaged. Individual residue-pair scores were scaled by dividing by the average score and residue-pairs with a scaled score less than 2.00 were removed. The Rosetta *ab initio* structure prediction protocol does not require all residues to be restrained and only the most confident predictions were used. The remaining scaled scores were then sorted and only the top scoring contacts (the number of contacts used was up to 1.5 times the sequence length of the target sequence) were converted into AtomPair constraints for the Rosetta constraint framework using the form described below (https://new.rosettacommons.org/docs/latest/rosetta_basics/file_types/constraint-file).

```
AtomPair {atomname_i} {res_i} {atomname_j} {res_j} SCALARWEIGHTEDFUNC
{scaled_score_ij} SUMFUNC 2 SIGMOID {nbr_dist_ij} 3.0 CONSTANTFUNC -
0.5
```

The distance between residue-pairs *i* and *j* in the structure prediction trajectory was constrained using a sigmoidal function between the "neighbor atoms" of each residue (Cβ atom except for glycine where Cα was used). The functional form of the constraint was

$$f_{sigmoid}(x) = 1/(1 + exp(-m \cdot (x - x_{0,ij}))) - 0.5$$

where *m* is the slope (fixed to 3.0 for all atom-pairs), *x* is the distance between the neighbor atoms of residues *i* and *j*, and $x_{0,ij}$, the center of the sigmoid function, is the sum of the "neighbor distance" for the two residue types. The neighbor distance is the radius of the sphere that represents the side-chain in Rosetta's centroid residue representation (neighbor atoms and distances for all residue types are shown in table below). The sigmoid function is reduced by a constant 0.5, so that it is bounded [-1,0), and then multiplied by the scaled score of the residue-pair. In contrast to harmonic or bounded constraints, sigmoid constraints are smooth and differentiable, and allow residues to slide into contact during the gradient based minimization

steps of the structure prediction protocol. The flattened tails of the sigmoid function ensure that unsatisfied constraints receive minimal penalties, satisfied constraints receive an almost constant bonus relative to the confidence in the prediction and at close distance the full atom energy function to determine fine-grained interactions.

Supplementary Table 1: Neighbor distance that represents the radius of the side-chain in centroid representation around the neighbor atom.

Residue	Neighbor Distance (Å)	Neighbor Atom Name
ALA	3.245	Cβ
ARG	5.640	Cβ
ASN	4.290	Cβ
ASP	4.250	Cβ
CYS	4.170	Cβ
GLU	4.950	Cβ
GLY	4.900	Cα
GLN	2.400	Cβ
HIS	4.660	Cβ
ILE	4.320	Cβ
LEU	4.760	Cβ
LYS	5.300	Cβ
MET	4.950	Cβ
PHE	4.815	Cβ
PRO	3.580	Cβ
SER	3.560	Cβ
THR	3.790	Cβ
TRP	4.910	Cβ
TYR	4.715	Cβ
VAL	3.900	Cβ

4. Rosetta model building on the IBM World Community Grid

We predicted the structure for each MIP sequence by generating 20,000 evolutionary contact-assisted (GREMLIN) models using Rosetta, in a project deployed on the IBM World Community Grid (WCG) called the Microbiome Immunity Project (MIP). The WCG is a citizen science initiative that allows researchers to perform calculations on volunteers' computers when they would otherwise be idle. A modified version of Rosetta (based on the 2016.32.58837 release) was used to generate all Rosetta models on the WCG. The modifications were cosmetic and provided a graphical animation of the protein structure prediction process for WCG participants that ran the graphical client program. No scientific code was modified and the equivalent command line and options file (a.k.a. "flags") are shown below.

```
% minirosetta.default.linuxgccrelease @/PATH/TO/MIP_${KEY}.flags -
out::suffix MIP_${KEY} -out::file::silent -jran ${RNG_SEED}
```

Contents of the command line flags file (`MIP_${KEY}.flags`) are shown below.

```
-database ./database

-in:file:fasta ./MIP_KEY.fasta
-in:file:frag3 ./MIP_KEY.1
-in:file:frag9 ./MIP_KEY.2

-abinitio:relax
-relax:fast
-abinitio::increase_cycles 10
-abinitio::rg_reweight 0.5
-abinitio::rsd_wt_helix 0.5
-abinitio::rsd_wt_loop 0.5

-use_filters true
-psipred_ss2 ./MIP_KEY.psipred_ss2

-nstruct 30
-run:constant_seed

-beta_nov15

-cst_file ./MIP_KEY.cst
-cst_weight 3

-cst_fa_file ./MIP_KEY.cst
-cst_fa_weight 3
```

The protein sequence, fragment files (described above), constraints based on predicted contacts (described above), secondary structure prediction of the target sequence (described above), and a random seed are provided as inputs. The random number generator seed was to ensure that all clients received a different seed. Identical constraints were used during both the centroid and full atom phases of the protocol. The REF2015 score function[13] was used during the full-atom phase of the protocol (called `beta_nov15` in this release of Rosetta). The model with the lowest energy score according to the REF2015 score function was used for function prediction and further analysis.

5. DMPfold model building

We additionally predicted the structures of all MIP sequences using DMPFold[14]. In contrast to fragment-based protein structure prediction techniques like Rosetta, DMPFold uses a consensus neural network to predict geometric constraints from the target sequence and uses them for structure calculations. Specifically, DMPFold takes as input a target protein sequence and generates multiple sequence alignments using HHBlits on the uniclust30 database. In this work, we instead used the alignments that were used for contact prediction for Rosetta. Then, DMPFold

uses a neural network to generate geometric constraints, which are then used in an iterative structure prediction pipeline using the Crystallography and NMR System (CNS), similarly to how NMR data are used for structure calculations. DMPFold produces up to 5 ranked structural models.

Representative command lines for both steps are shown below. The “map” and “21c” files are intermediate files generated in the alignment step. DMPFold was run with the default 3 iterations and 50 models per iteration. The best ranking model, “final_1.pdb” was used for function prediction and further analysis.

```
% csh seq2maps.csh MIP_${KEY}.fasta

% bash run_dmpfold.sh MIP_${KEY}.fasta MIP_${KEY}.21c MIP_${KEY}.map
models 3 50
```

3. Function prediction

To further understand the sequence, structure, and functional relationships in our dataset, we predicted the function of the lowest energy Rosetta models and highest-ranking DMPFold models for all MIP sequences using DeepFRI[15]. DeepFRI uses a graph convolutional neural network to predict protein function from sequence and/or structure, yet does not use homology transfer to accomplish the predictions. It has been trained to predict both Gene Ontology (GO) and Enzyme Commision (EC) identifiers. DeepFRI's prediction performance on predicted protein models is similar to ones on experimentally determined structures. Additionally, DeepFRI can be used for class activation mapping to indicate which residues are the most salient for the predicted functions. DeepFRI takes protein structures (from which it extracts the protein sequence and constructs a residue-residue pair contact maps), and a set of trained weights as input. It outputs a vector of scores where each element in the vector is assigned to a specific function and scores range from 0 to 1. This means that each protein has multiple different functions that don’t compete with one another.

In this work, we used an updated set of weights trained on the SIFTS 2021 database release called the “newest trained models” available (https://github.com/flatironinstitute/DeepFRI). Generating class activation maps increases the runtime and was not performed on the full dataset. The command lines for batch prediction on a directory of PDB formatted protein structures are shown below. Each of the top level GO classifications, and the EC identifiers need to be run separately; molecular function (mf), cellular component (cc), biological process (bp), and enzyme commission (ec).

```
% python DeepFRI/predict.py --verbose --model_config
newest_trained_models/model_config.json --saliency --use_guided_grads
--verbose --ont {cc,mf,bp,ec} --pdb_dir /PATH/TO/PDB_DIR
```

The confidence metric for DeepFRI should be interpreted in the following way: DeepFRI scores > 0.5 have a high confidence, which is mostly due to high occurrences in the training dataset and which often happens for more general functions at the top of the GO hierarchies. DeepFRI scores < 0.2 don't necessarily indicate an incorrect prediction, but rather that the number of examples in the training dataset is low. DeepFRI is further trained such that scores propagate along the GO tree with higher scores towards the top of the tree and lower scores at the leafs, meaning that DeepFRI produces self-consistent predictions and does not "randomly" sample the GO tree. We often find that DeepFRI scores of 0.2 or even lower are perfectly adequate as indicated by very similar predictions for vastly different sequences (yet same structures), which is another indication of confidence in our predictions (see Fig. 4).

The plot below shows the performance of DeepFRI on the independent test set that was used for evaluating the trained DeepFRI model - this test set had < 30% sequence identity to the training set. Here we evaluate the precision, recall, and F1-score for the function prediction vectors above a certain threshold, shown on the x-axis. For the molecular function branch of the GO-tree, a threshold of 0.2 gives an F1-value > 0.5 for the GCN (=GNN) which in our eyes is sufficiently confident for using this threshold for the prediction. We provide the scores here for the branches across the GO tree and Enzyme Commission number to allow anyone using DeepFRI to make their own decision on what prediction threshold they feel most comfortable with.

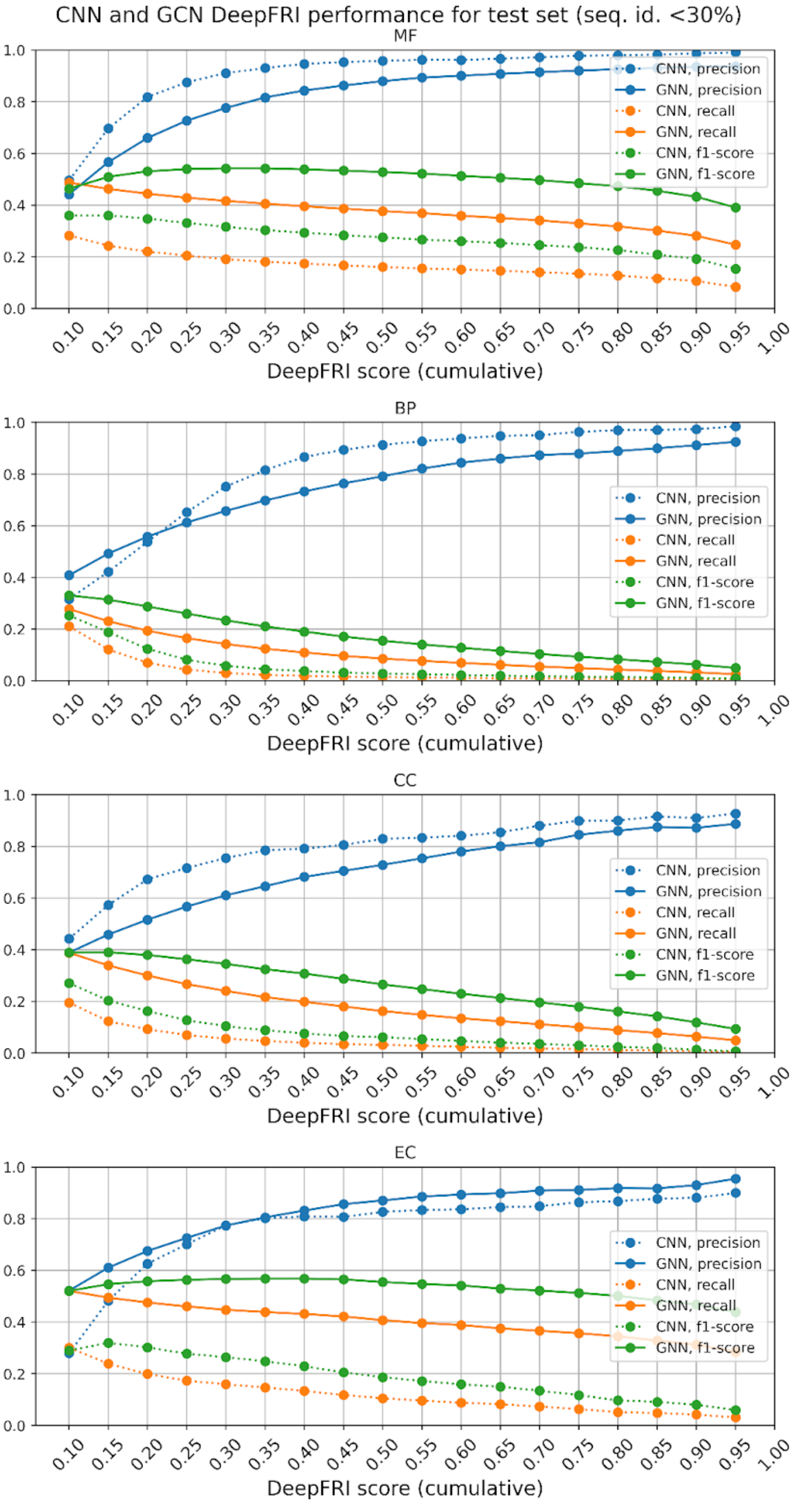

Supplementary Figure 1. Precision, recall, and F1-score for the DeepFRI test set that had less than 30% sequence identify to the training set, for the three branches of the GO tree (Molecular Function (MF), Biological Process (BP), Cellular Component (CC)) and Enzyme Commision numbers (EC).

For functional similarity, we used cosine similarity between DeepFRI vectors thresholded at 0.1 (the non-zero threshold is important for denoising). DeepFRI was developed such that output scores are normalized between 0 and 1 and cosine similarity of two DeepFRI score vectors is a normalized sum of products of positive values (and therefore cannot be negative). We used cosine similarity as a similarity metric of two function prediction vectors because proteins have multiple functions and therefore DeepFRI predictions are vectors in a multi-dimensional function space. If two proteins have the same vectors, they would have the same function. If two proteins have the same functions but with slightly different scores, the function vectors would have a similar direction in that multi-dimensional space, therefore using cosine similarity as a similarity metric makes intuitive sense. In contrast, if two proteins have very different functions, the elements with non-zero scores in the two vectors would differ and therefore the cosine similarity would be zero or close to zero, meaning the directions of the score vectors in the multi-dimensional space would be different.

Supplementary Note 2: MIP dataset descriptions

Datasets were created in the following order:

(1) *MIP_raw* is the starting dataset with all entries, containing both Rosetta and DMPFold models of varying quality from both high-quality to low-quality models.

(2) We used 5,000 random entries from the *MIP_raw* dataset to derive model quality metrics – this dataset is denoted *MIP_random5000_raw*.

(3) When filtering out the low-quality models from *MIP_random5000_raw*, we are left with 3,052 high-quality models, denoting *MIP_random5000_curated*

(4) When filtering out the low-quality models from the entire *MIP_raw* dataset, we get the *MIP_curated* dataset that only contains high-quality models.

(5) We also created a subset of *MIP_curated* for visualization purposes, these are 10,000 pairs of high-quality Rosetta and DMPFold models.

The tables below describe our datasets and the number of models in each dataset. Note that there is partial overlap between the datasets of Rosetta models and DMPFold models because either DMPFold didn't converge, or Rosetta models weren't completed due to interrupted runs on the World Community Grid.

Supplementary Table 2: Definitions of the MIP datasets.

Dataset	Description
MIP_raw	All MIP entries. Includes both Rosetta and DMPFold models.
MIP_curated	Highest quality dataset created by filtering *MIP_raw* by quality metrics. Used for most quantitative studies in the main paper and the supplement.
MIP_visualization	Representative dataset from *MIP_curated* for visualization purposes; randomly sampled 9839 MIP IDs and added 161 representatives of novel fold clusters (in total: 10,000 points for DMPFold + 10,000 for Rosetta + 6631 CATH representatives).
MIP_random5000_raw	5000 random entries from *MIP_raw* with IDs common between Rosetta and DMPfold sampled by length distribution. The dataset was used to estimate the quality metric thresholds for constructing the *MIP_curated* dataset.
MIP_random5000_curated	The curated part of MIP random5000_raw was used to compute pairwise similarity measures in Fig. 2.

Supplementary Table 3: Number of structures in the MIP datasets.

	Rosetta models	DMPFold models	CATH superfamilies	Common Rosetta-DMPfold models
MIP_raw	245,443	241,834	0	240,703
MIP_curated	211,069	203,877	0	184,642
MIP_visualization	10,000	10,000	6,631	10,000
MIP_random5000_raw	5000	5000	0	5000
MIP_random5000_curated	3052	3052	0	3052

1. MIP dataset curation

To identity high quality models in the *MIP_raw* dataset we used the following quality measures (MQA - model quality assessment):

- Rosetta: average TM-score between the 10 structures with the lowest energies
- DMPfold: quality metric of the structural model that describes the agreement between the neural-network predicted contact map and the contact map back-computed from the final model

The metrics, together with other parameters (e.g. Stride[16] output), were then analyzed (independently for Rosetta and DMPFold) using the Random5000_raw dataset - see section *Random5000 data analysis* below for details. As a result, the *MIP_curated* dataset was constructed based on the following criteria:

- Rosetta: MQA >= 0.4 and coil content ≤ 60%
- DMPFold: MQA >= 0.4 and coil content ≤ 80%

The coil content was computed as a sum of T and C Stride annotations normalized by sequence length. In Supplementary Figure 2 we show how the above criteria shift the density distribution between *MIP_raw* and curated i.e. we can notice higher agreement between Rosetta and DMPFold models for the latter.

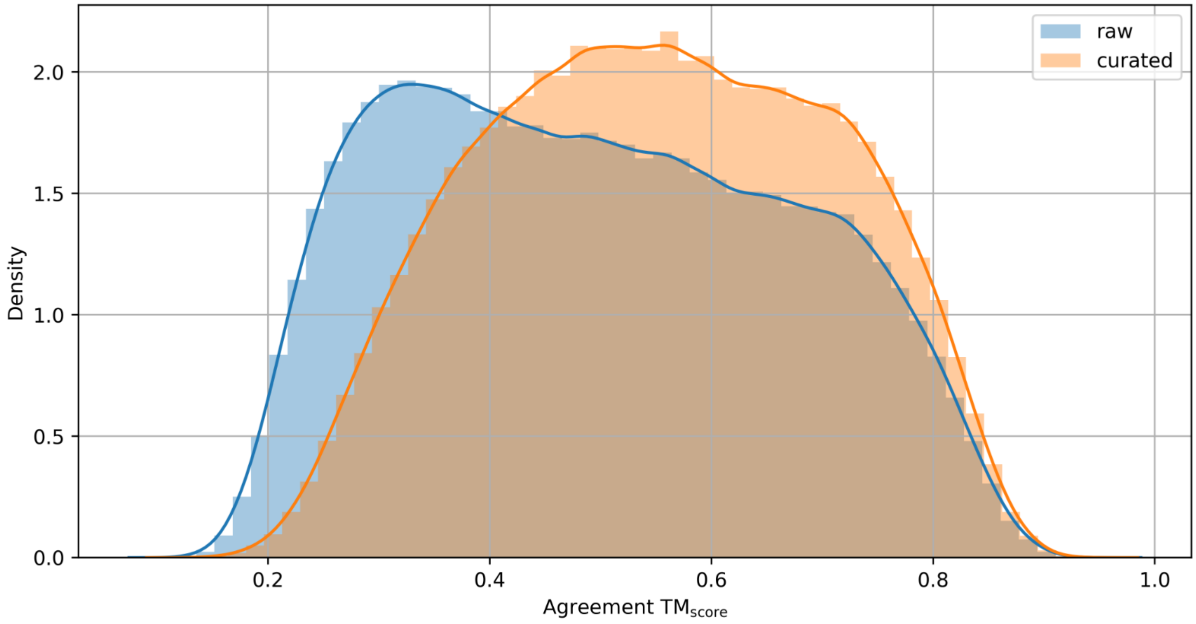

Supplementary Figure 2. Density plot of MIP structures as a function of structural similarity (agreement TM-score between Rosetta and DMPFold models) for MIP_raw and MIP_curated.

2. Sequence length distribution

MIP sequences are relatively short (between 40 and 200 residues) - see upper panel in Supplementary Figure 3. Comparing sequence lengths distributions for *MIP_curated* (lower panel in Supplementary Figure 3) we see that more short DMPFold models (<100 residues) were discarded, as were more longer Rosetta models (>100 residues), compared to the *MIP_raw* dataset. However, the overlap (common part) of the models that survived is still high (89%) - see also Supplementary Table 3.

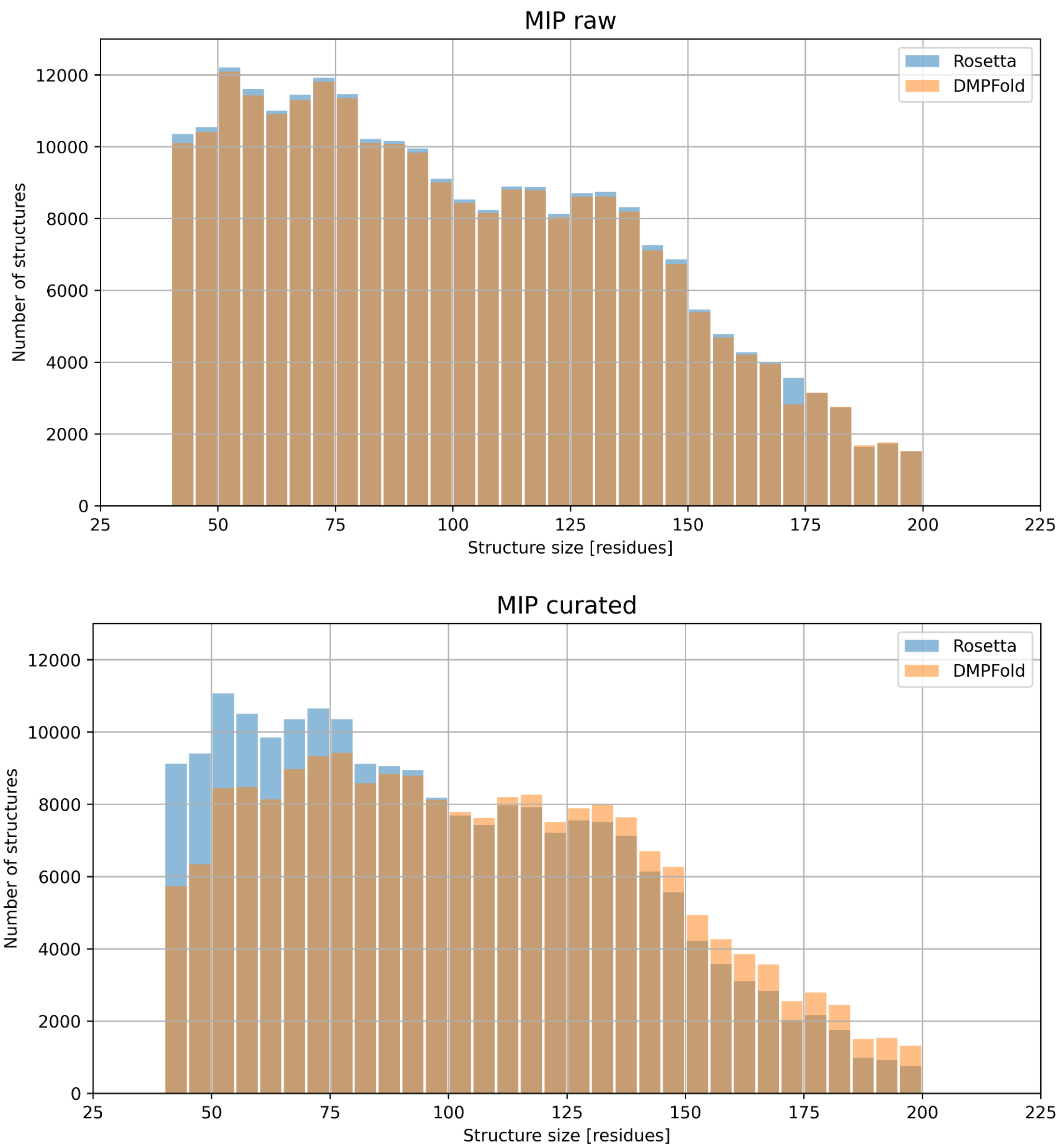

Supplementary Figure 3. Sequence length distributions for MIP_raw and MIP_curated.

3. Pairwise structural comparisons in the curated dataset via CATH

Our next goal was to identify how structurally similar any two MIP models were to each other, to find clusters of similar structures and to find novel folds. However, this would have required 200,000 x 200,000 / 2 = 20 billion comparisons which is computationally prohibitive. Instead, we decided to compare each of our *MIP_curated* models to CATH superfamilies, of which there are about 6,000, and then compare CATH superfamilies to each other. This led to 6,000 x 6,000 / 2 = 18 million comparisons, which was far more tractable.

To accomplish this, all models in the *MIP_curated* dataset were superimposed with CATH 4.3.0 superfamily structures using TM-align (see Methods section in the main paper). For each model we then found the most similar CATH representative (the largest TM-score normalized by MIP

sequence length) and annotated the model by CATH hierarchy levels (class, architecture, fold, superfamily) assigned to that CATH structure. We found that approx. 70% of CATH superfamilies were identified as the closest structural homologs for the Rosetta curated dataset and approx. 65% for the DMPFold curated set. The percentages drop slightly (to 68% and 62% respectively) when considering only structures with TM-scores >= 0.5.

Overall, Rosetta predicts more alpha-helical models than DMPFold and inversely for beta and alpha/beta models (left panel of Supplementary Figure 4). The difference even grows with TM-score (better quality of annotations). The above conclusion also holds on the architecture level (right panel in Supplementary Figure 4). For all major architectures in each CATH class we notice similar differences between Rosetta and DMPFold.

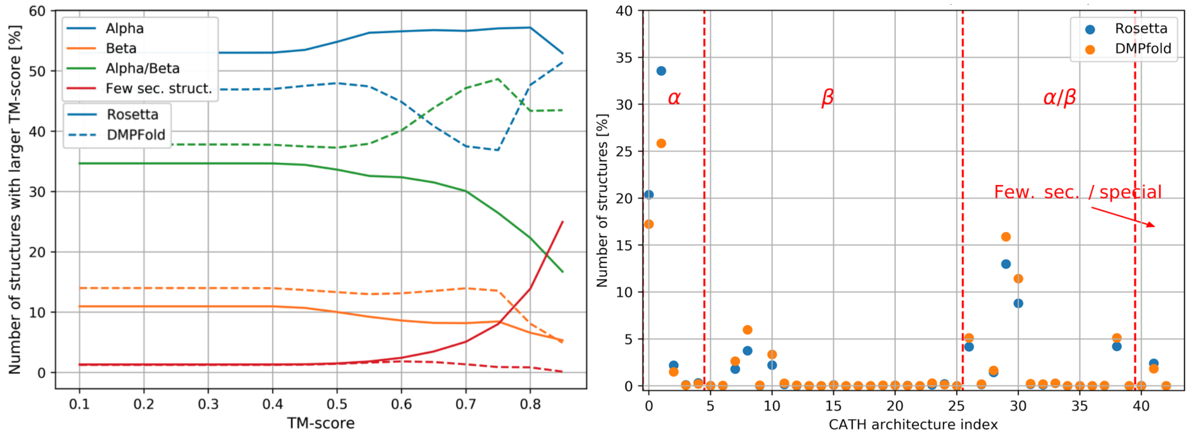

Supplementary Figure 4: Left: Comparing the models in the MIP_curated dataset against CATH classes and plotting the TM-score over the percentage with the specific classes. Solid lines for Rosetta models, dotted lines for DMPFold models. For a TM-score around 0.7, DMPFold the percentage of models switches between alpha and alpha/beta proteins. With increasing TM-score, the percentage of Rosetta models with few secondary structure elements increases while the alpha/beta percentage decreases. Right: percentage of structures in MIP_curated datasets (Rosetta, DMPFold) for a given CATH architecture (only structures with maximum TM-score against CATH >= 0.6 were considered).

Supplementary Note 3: Model quality assessment on the MIP_random5000 dataset

1. Explanation of variables

N_eff

N effective is the number of sequences in the multiple sequence alignment that each MIP entry has. For contact prediction, the larger N_eff is, the higher quality the predicted contacts and therefore the models are. It has previously been suggested that N_eff has to be at least 5x the sequence length to produce high-quality contacts during prediction. However, we use the lower limit of 16 sequences required to predict contacts using GREMLIN[11].

Sequence length

The raw sequences that came out of sequencing were split into domains based on sequence-based predictions. These domains were given a MIP ID entry and their sequence length was based on this domain sequence.

Secondary structure
The sequence for each MIP ID was used for structure prediction both with DMPFold[14] and Rosetta *abinitio* modeling using contact restraints predicted from GREMLIN[11]. Both DMPFold and Rosetta models were run through Stride[16] to extract the secondary structure definitions for each residue. Stride classifies residues into the following secondary structures: H – alpha helix; G – 3-10 helix; I – PI-helix; E – extended conformation; B or b – isolated bridge; T – turn; C – coil (none of the above). To create a 3-state secondary structure definition, we grouped H, G, and I into a helix definition; E and Bb into strand definition; and defined (1 - helix - strand) as coil. Coil percentage is defined as the number of coil residues over the sequence length; similar for helix and strand.

Membrane content
The sequence for each MIP ID was used to predict transmembrane helices through OCTOPUS[17] and transmembrane strands through BOCTOPUS[18]. The membrane-helix percentage is the number of residues predicted as a transmembrane helix divided by the sequence length; similar for membrane-strand prediction.

Disorder
Protein disorder was predicted from the sequence of each MIP entry using MobiDB-lite[19] v.1.0 (March 2016) and DISOPRED3[20] (zip downloaded on September 16, 2021; dependencies: Blast v.2.2.26, Uniref90 v.20210731).

TM-score to CATH
Predicting the structures of millions of proteins raises the question of how many novel folds are predicted in this dataset. This requires each new model to be compared with known protein structures. CATH[21] is a database that contains all known non-redundant protein domains present in the Protein Data Bank[22]. An advantage is the small size of the database encompassing ~6000 domains, as compared to ~150,000 structures in the PDB, minimizing runtime. We use the TM-score[23] ('template modeling score') as a structural similarity measure which falls between 1 for identical folds, to 0 for different folds. From a technical perspective, the TM-score rarely falls below 0.2 and a cutoff of 0.4 is often used to distinguish novel folds (TM-score ≤ 0.4) from known folds (TM-score > 0.4). The TM-score is computed as

$$\text{TM-score} = \max\left(\frac{1}{L_{target}} \cdot \sum_{i}^{L_{aligned}} \frac{1}{1 + \left(\frac{d_i}{d_0(L_{target})}\right)^2}\right)$$

where L_{target} and $L_{aligned}$ are the lengths of the protein target and the aligned regions, respectively. d_i is the distance between the i-th pair of residues and

$$d_0(L_{target}) = 1.24 \cdot \sqrt[3]{L_{target} - 15} - 1.8$$

is a scaling factor that normalizes distances.

Agreement TM-score

Because we have two models for each MIP ID, a DMPFold and a Rosetta model, we can compute a TM-score of the Rosetta model (aligned) to the DMPFold model (target) and vice versa. This leaves us with two TM-scores, the average of which we define as the agreement TM-score. Like the TM-score, the agreement TM-score falls between 0 and 1, with 1 being ideal model structural agreement.

Contact order

The contact order is a measure of fold complexity. The absolute contact order is computed as

$$Absolute\ contact\ order = \frac{1}{N}\sum^{N} \Delta S_{ij}$$

with N being the total number of contacts and ΔS_{ij} being the sequence separation between residues i and j. The relative contact order is the absolute contact order normalized by the sequence length, therefore being length independent.

MQA score

We sought a model quality assessment score that is fast to compute, is ideally a single model score and tells us something about how 'good' the overall model is, irrespective of details such as bond lengths, bond angles, rotamers etc. We considered the VoroMQA score, yet ultimately refrained from using it for reasons mentioned below. We decided on a MQA score that is dependent on the method used. We know that Rosetta is often able to find the global minimum of an energy landscape, especially looking at the top 10 (i.e. 10 lowest-energy) models from 20,000 models (a.k.a. 'decoys'). Even if the top model belongs to a false minimum on the energy landscape, we know from experience that some models among the top 10 are close to the 'native' structure if an experimental structure exists. In the vast majority of cases, clustering the top 10 models and looking at the largest cluster, will find the 'correct' solution. While this exact approach is somewhat cumbersome and computationally inefficient, we use a shortcut which we show below seems to work just as well: we compute the average of the pairwise TM-scores between the top 10 Rosetta models. Again, this TM-score is in the range between 0 and 1. The higher this Rosetta MQA score, the more of the top 10 models have the same structure, supporting the notion that Rosetta has found the global minimum in this case. Therefore, the MQA score also encodes our confidence in the correctness of the Rosetta model.

DMPFold is unfortunately inconsistent and outputs 1-5 final models, however the models have a confidence score associated with them. We use this confidence score as MQA score for the DMPFold model.

Rosetta score

Each Rosetta model has a score associated with them which represents the 'energy' of the model in the energy landscape of the Rosetta scoring function. The total score, which we are using here, is a weighted combination of different score terms, encoding both physics-based and knowledge-based terms. The total Rosetta score is not normalized by protein length. A detailed review of the Rosetta scoring function was recently published[13].

2. DMPFold models have higher coil content than Rosetta models

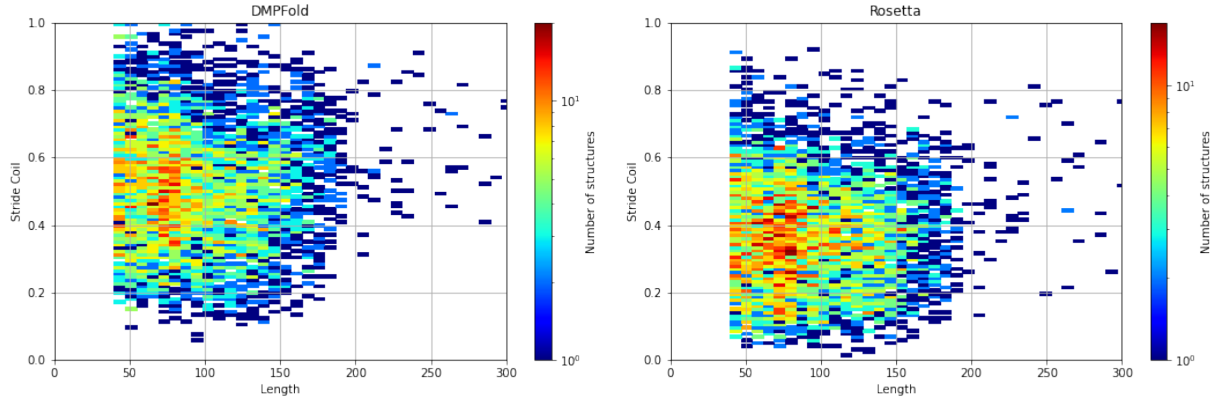

Supplementary Figure 5: Percentage of coil residues in the models, identified by Stride, over the sequence length. DMPFold models have a higher percentage of coil residues due to local inaccuracies in backbone torsion angles. DMPFold uses evolutionary restraints from contact prediction and predicts the structure using the CNS software under the influence of a molecular dynamics force field. Visually analyzing a large number of models indicates that Rosetta's fragment-based approach (in conjunction with contact constraints) and its scoring function are able to produce better local features in these models, while DMPFold is able to produce higher quality models of larger proteins.

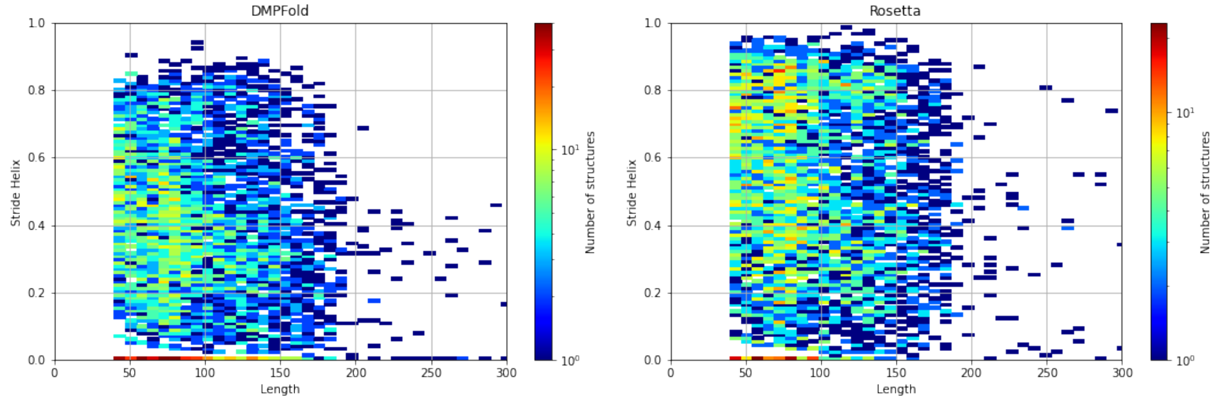

Supplementary Figure 6: Percentage of helical residues in DMPFold and Rosetta models over the sequence length. Rosetta models generally have a higher percentage of helical residues than DMPFold models.

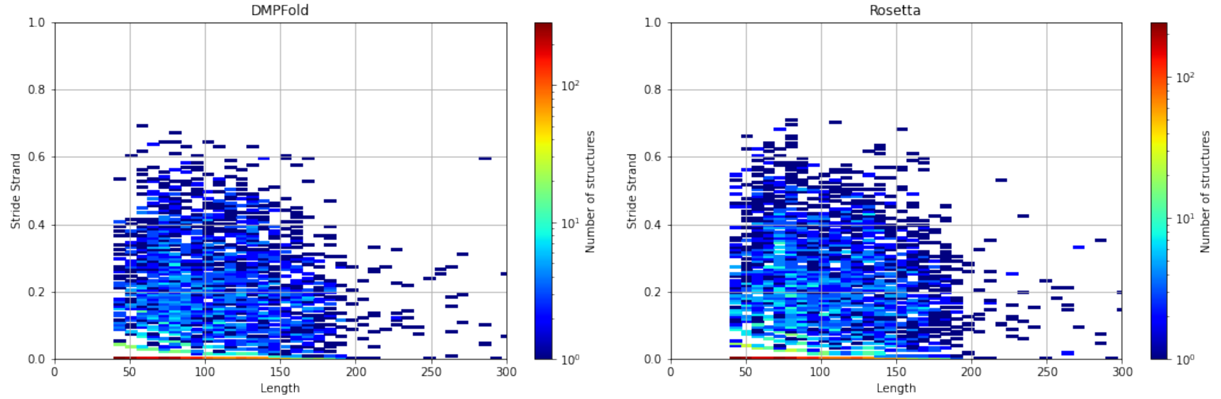

Supplementary Figure 7: Percentage of strand residues in DMPFold and Rosetta models over the sequence length. Rosetta models have a slightly higher percentage of strand residues than DMPFold models.

3. Model quality is independent of sequence length and correlates with agreement between Rosetta and DMPFold models

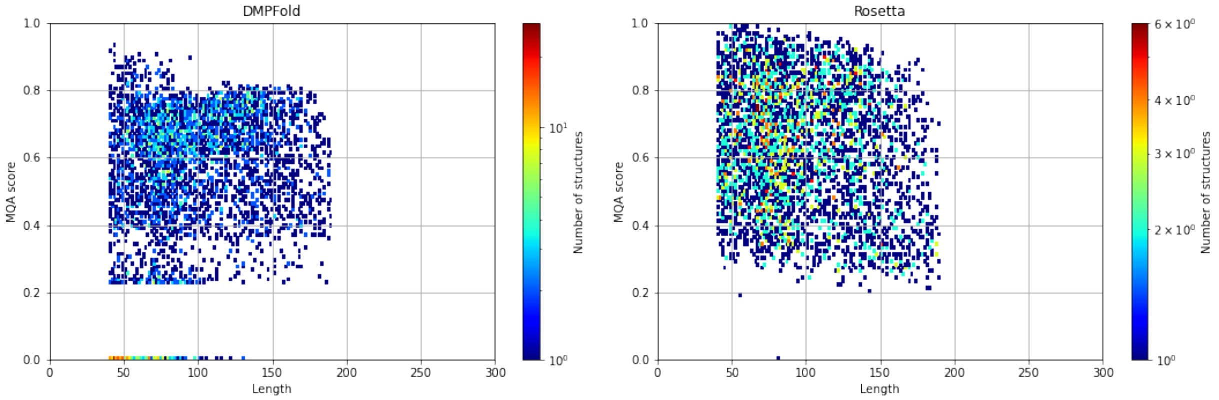

Supplementary Figure 8: The MQA score is largely independent of protein length. While there is no correlation for DMPFold models, the MQA score limits slightly decrease for Rosetta models. Still, this lack of correlation is a major advantage for using this MQA score in model quality assessment, in contrast to, for example the VoroMQA score24, which we tested.

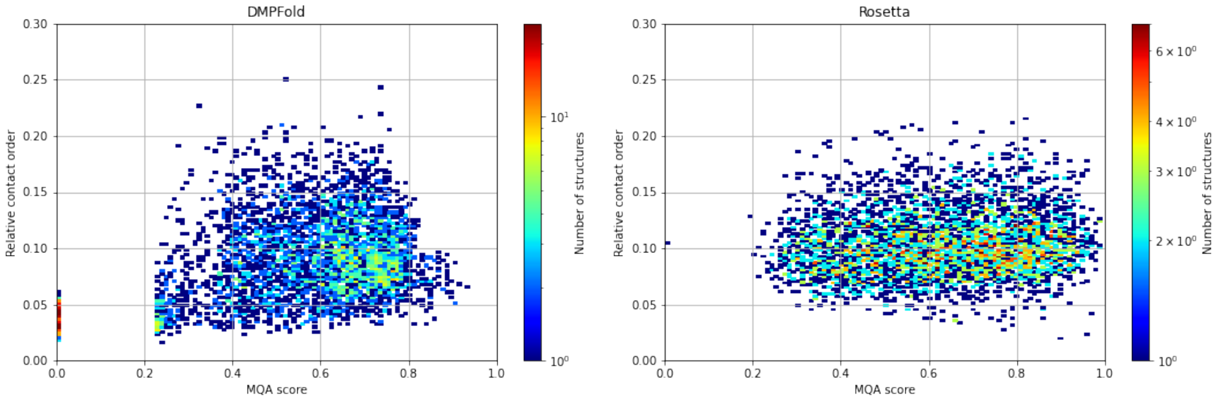

Supplementary Figure 9: The MQA score does not correlate with relative contact order, making it independent of the protein size. This is another indication that the MQA score is a good measure to evaluate model quality.

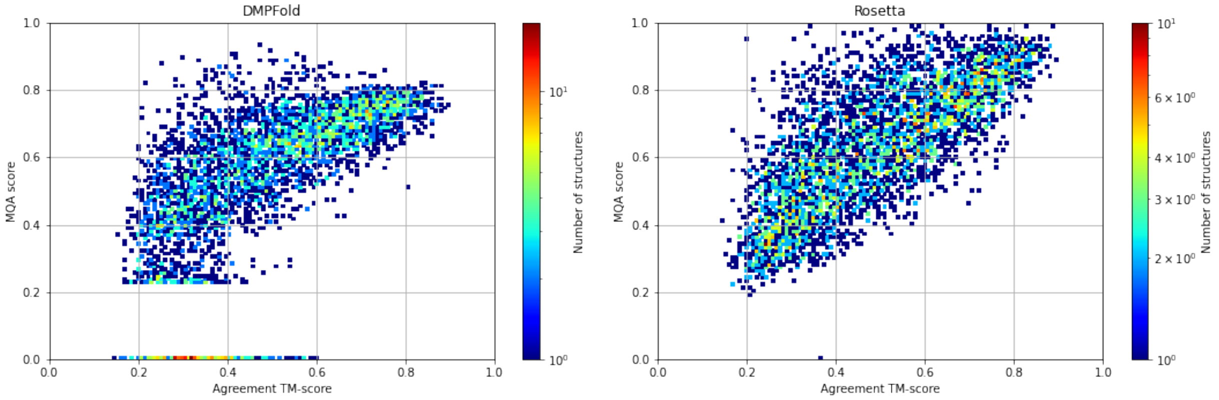

Supplementary Figure 10: The agreement TM-score between Rosetta and DMPFold models correlates with the MQA score, making the latter a good metric for model quality. The higher the model quality is, the higher the MQA score and the more likely it is that the DMPFold and Rosetta models are similar to each other. This works especially because the

structure prediction methods use contact-predicted constraints which have a high impact on the total score and model quality.

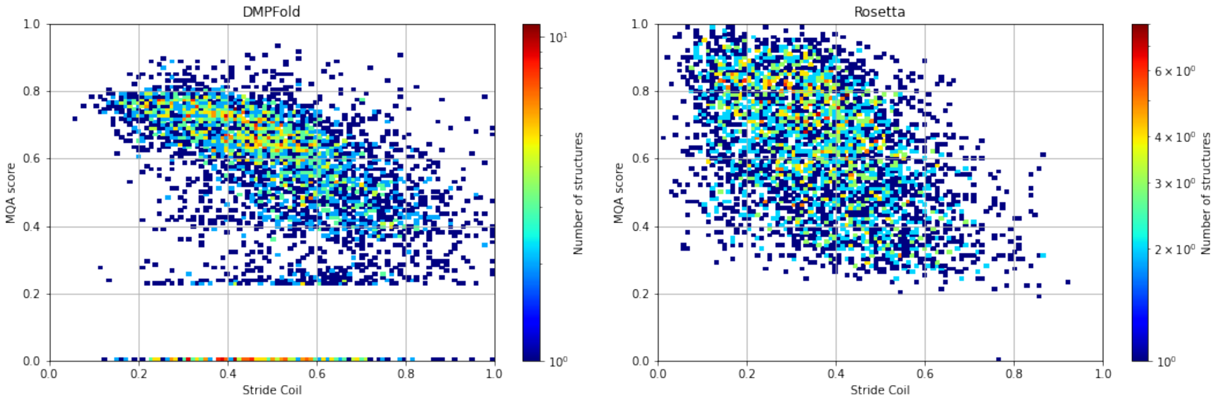

Supplementary Figure 11: The MQA score decreases with increasing coil content because these models have less defined secondary structure.

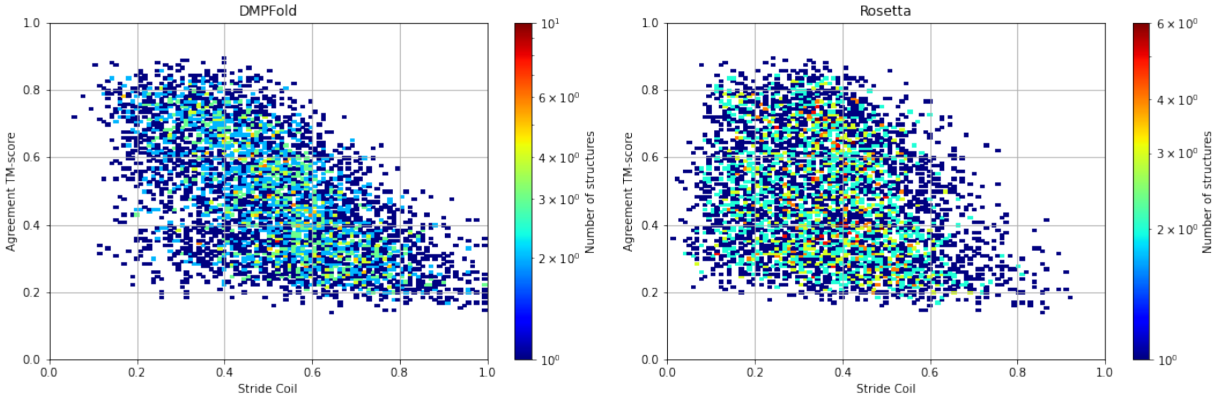

Supplementary Figure 12: The agreement TM-score between DMPFold and Rosetta models decreases as a function of coil percentage. Models with a higher percentage of coil have worse quality and therefore less agreement between DMPFold and Rosetta.

4. Deeper Multiple Sequence Alignment leads to higher model quality

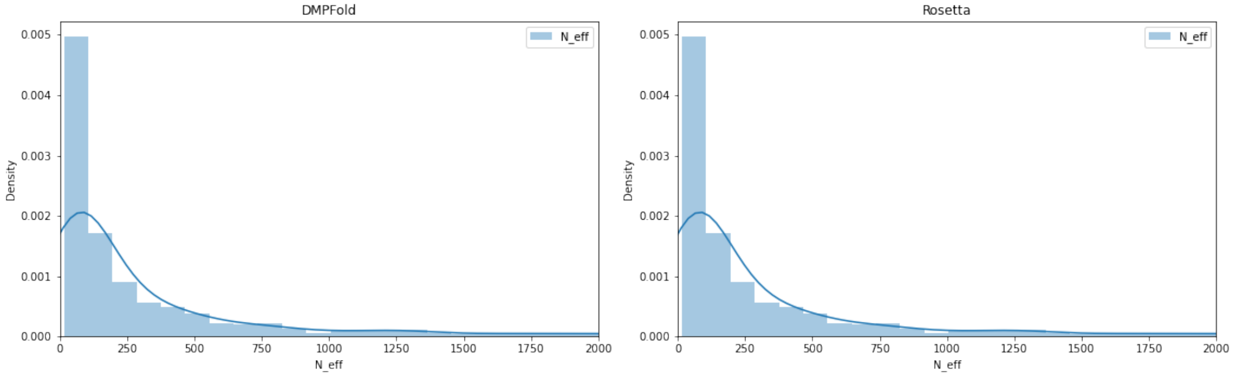

Supplementary Figure 13: Our models are predicted de novo using restraints from contact prediction methods derived from Multiple Sequence Alignments. The majority of MIP entries has less than 250 effective sequences in their multiple sequence alignment, as indicated by N_eff.

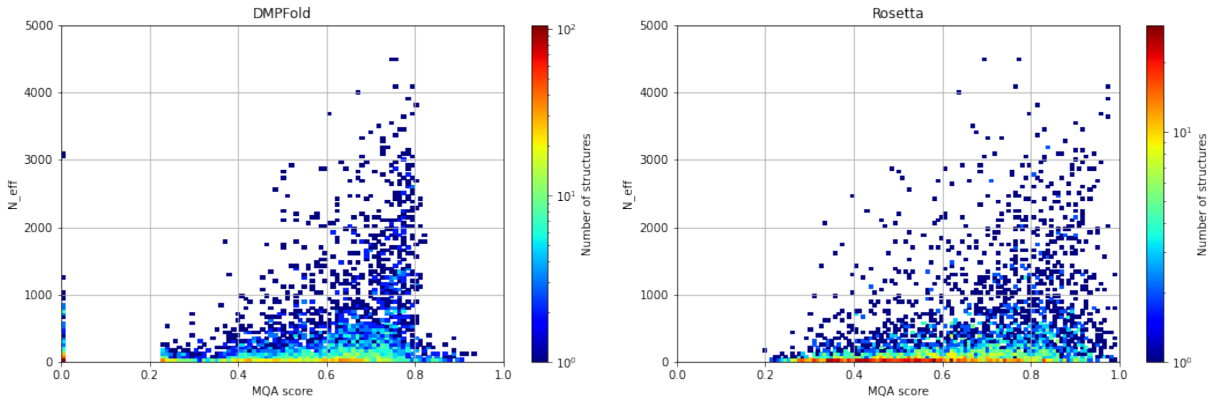

Supplementary Figure 14: A higher N_eff leads to higher quality models for DMPFold, as indicated by its MQA score. Similarly, a higher N_eff leads to better quality models for Rosetta, using the Rosetta MQA score. Comparing both methods, the same number of sequences in the MSA leads to higher MQA scores in Rosetta as compared to DMPFold. However, MQA scores for Rosetta and DMPFold were derived independently and are therefore not perfectly comparable.

5. Transmembrane spans

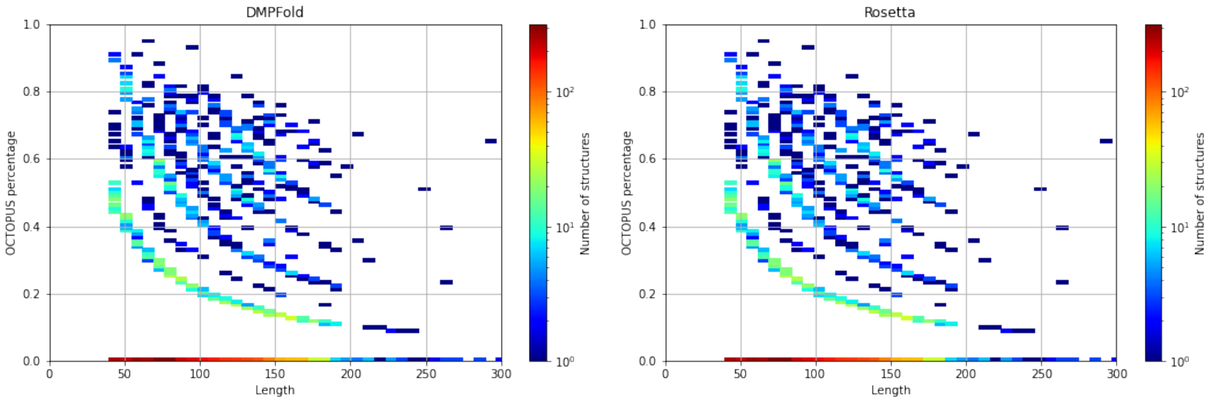

Supplementary Figure 15: Percentage of transmembrane helix residues predicted by OCTOPUS over the sequence length. The decay of the curves is a feature of the number of transmembrane helices as normalized by the sequence length. The bottom curve is a single transmembrane helix, the curve directly above is for two helices, and so on.

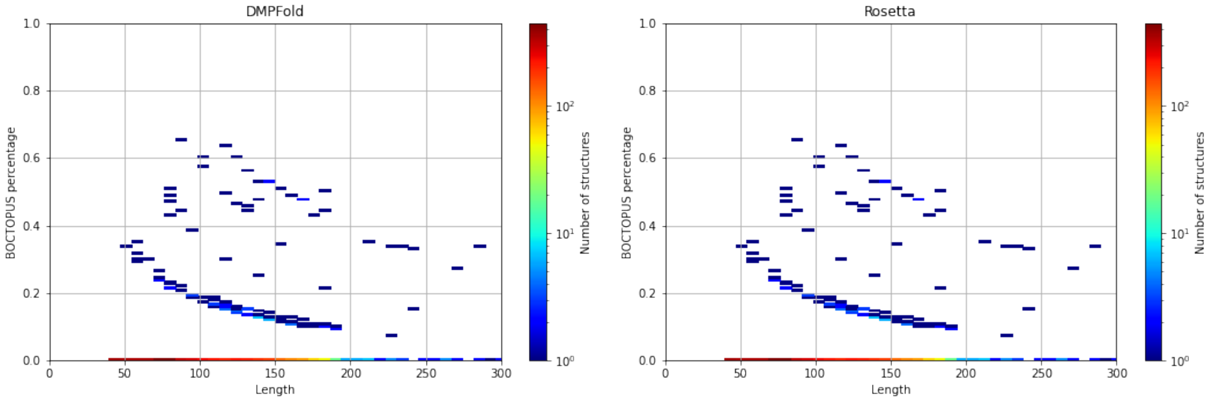

Supplementary Figure 16: Percentage of transmembrane strand residues predicted by BOCTOPUS over the sequence length. The decay of the curves is a feature of the number of transmembrane strands as normalized by the sequence length.

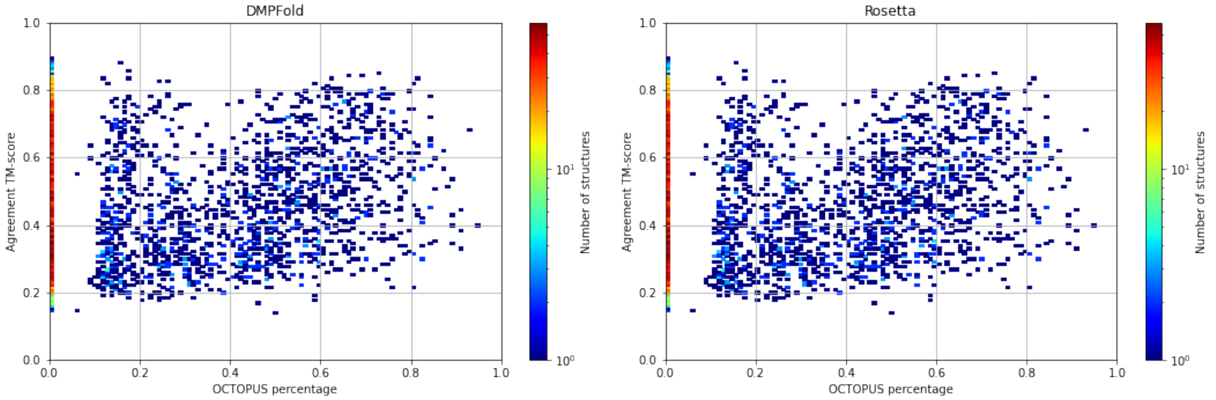

Supplementary Figure 17: Agreement TM-score between Rosetta and DMPFold models as a function of the percentage of transmembrane helices as predicted by OCTOPUS.

6. Disorder

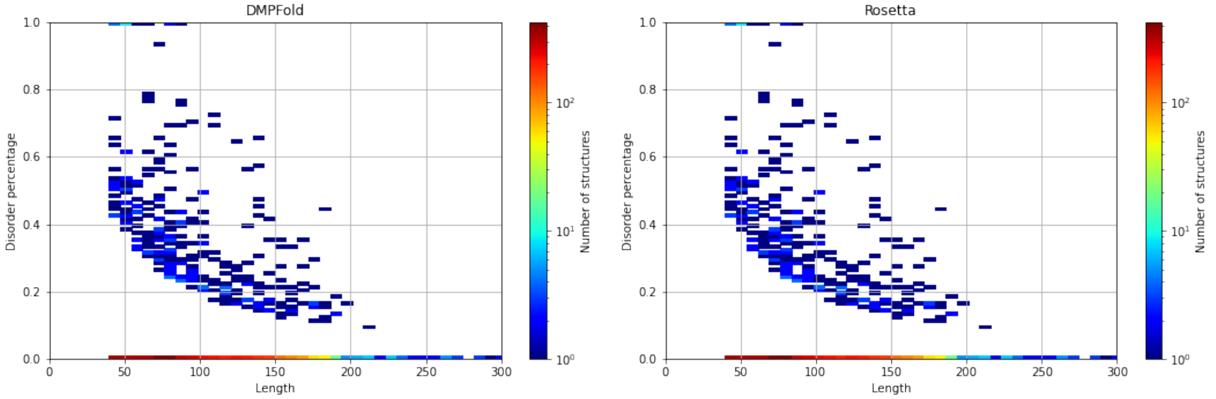

Supplementary Figure 18: Percentage of protein disorder as predicted by DISOPRED3, plotted over the sequence length. Again, the decay of the curve is a feature over the normalization by sequence length.

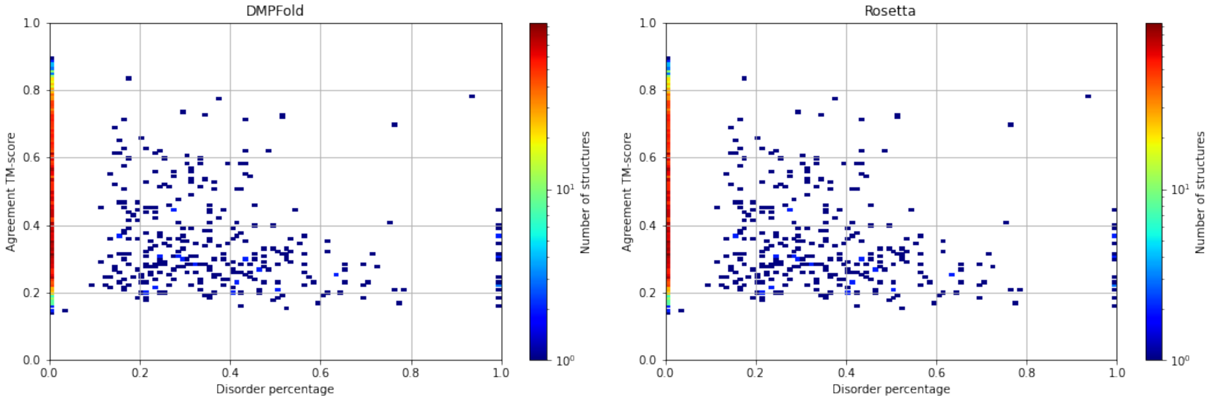

Supplementary Figure 19: The agreement TM-score between DMPFold and Rosetta decreases with increasing disorder percentage. This originates in a larger set of possible conformations between DMPFold and Rosetta and in decreasing model quality of higher disorder conformations.

7. Model complexity

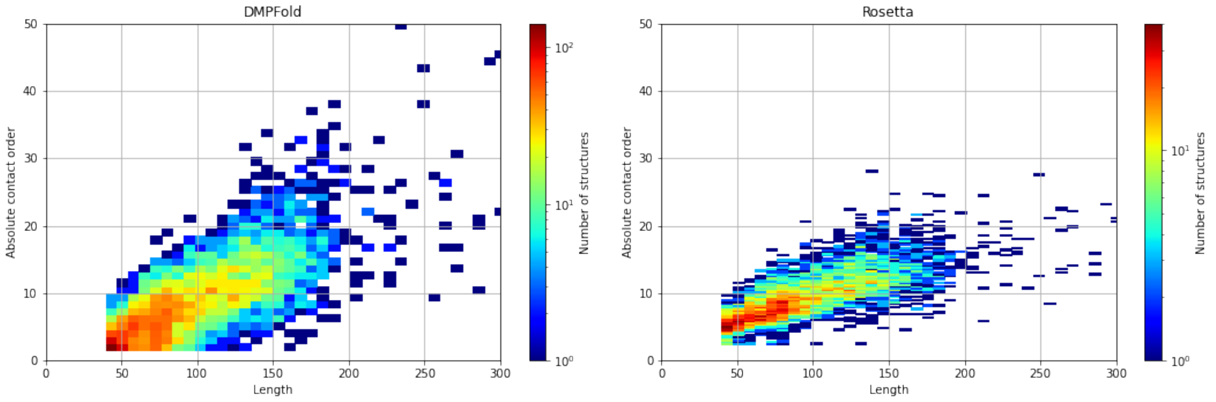

Supplementary Figure 20: The absolute contact order correlates with sequence length. The slope is larger for DMPFold than for Rosetta because DMPFold is better at predicting larger protein models, unlike Rosetta, which was originally designed to predict small, soluble proteins. Note that the absolute contact order is length dependent.

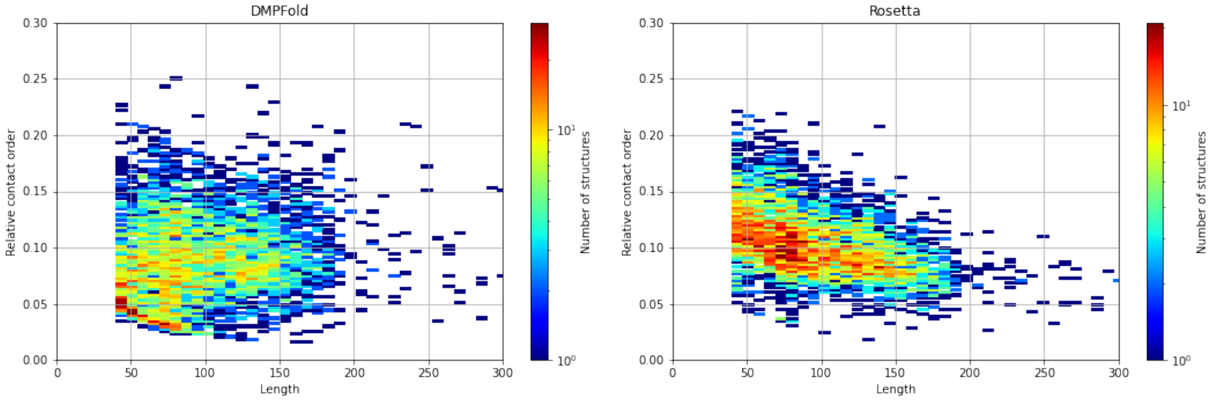

Supplementary Figure 21: The relative contact order, which is the absolute contact order normalized by length, as a function of the sequence length. DMPFold models do not exhibit a dependence of the relative contact order on sequence length, whereas Rosetta models decrease in their relative contact order, because it is more difficult to predict larger proteins with Rosetta.

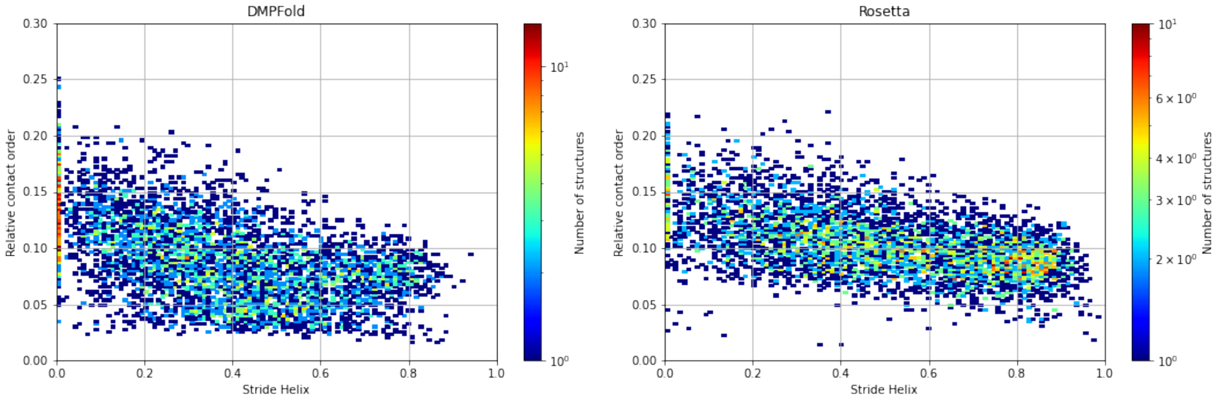

Supplementary Figure 22: The relative contact order decreases with the percentage of helix residues. There are many proteins in the MIP dataset that have one or two helices. These have a high helical content but a small contact order. Also, contact order increases with long-range contacts, yet helices have local contacts between residues.

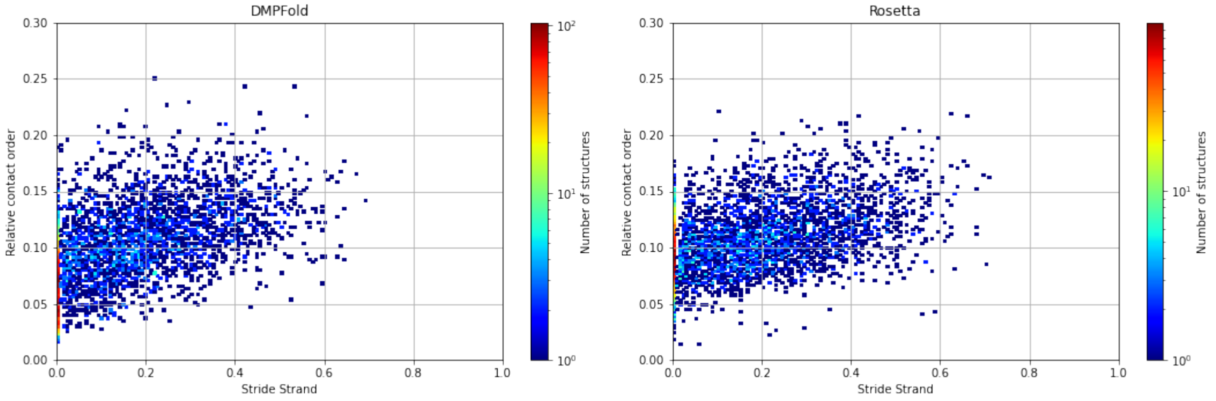

Supplementary Figure 23: The relative contact order as a function of strand content. The higher the strand content, the higher the contact order because strands are involved in long-range contacts.

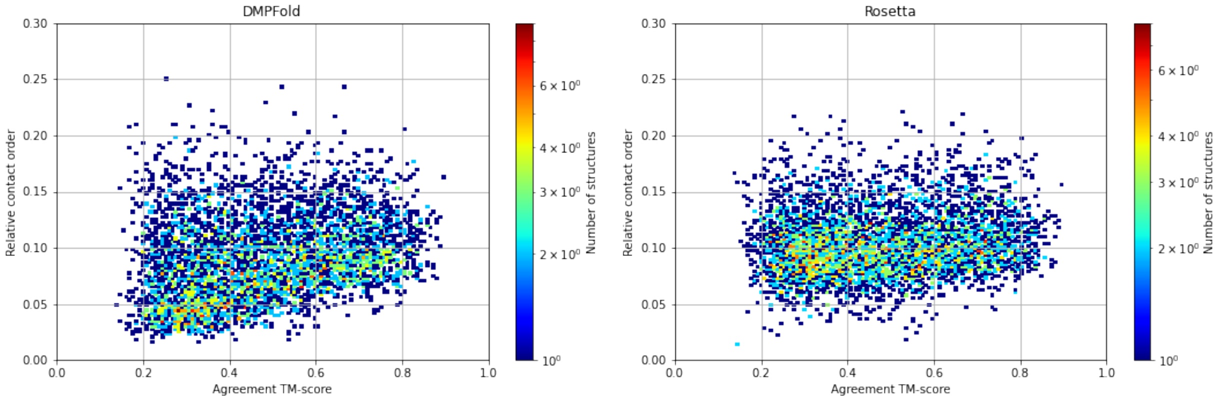

Supplementary Figure 24: The agreement TM-score increases slightly with the relative contact order for DMPFold models, but not for Rosetta models.

8. TM-score to CATH

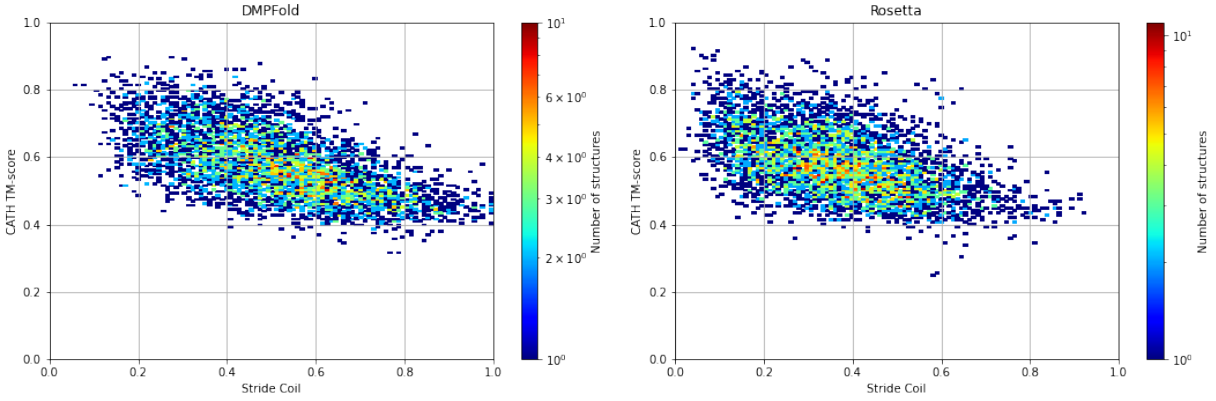

Supplementary Figure 25: The TM-score to CATH superfamilies as a percentage of coil residues in the models, indicating a slightly negative correlation. The more unstructured the protein is, the harder it is to find a match in CATH and the lower the TM-score will be because of structural differences.

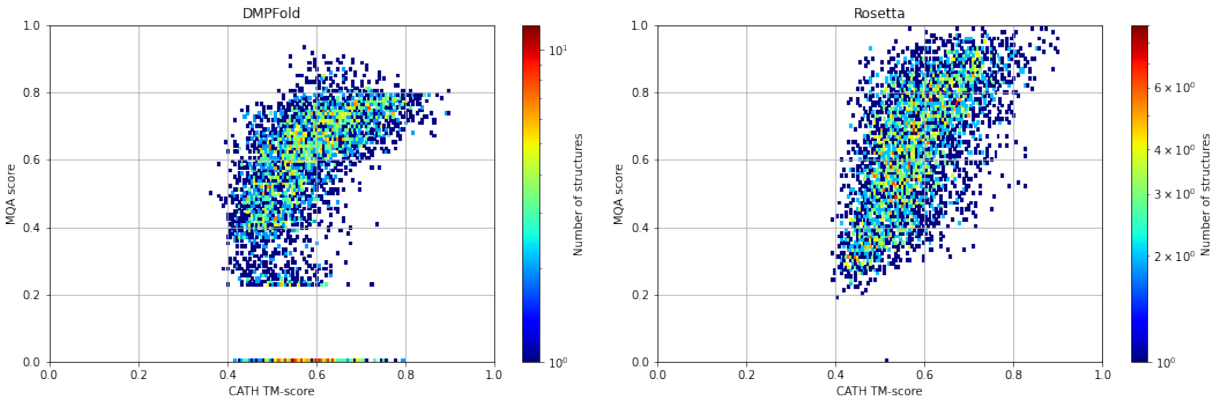

Supplementary Figure 26: There is a small correlation between model MQA score and TM-score to CATH, meaning the higher the model quality is, the more likely it is to find a match in CATH.

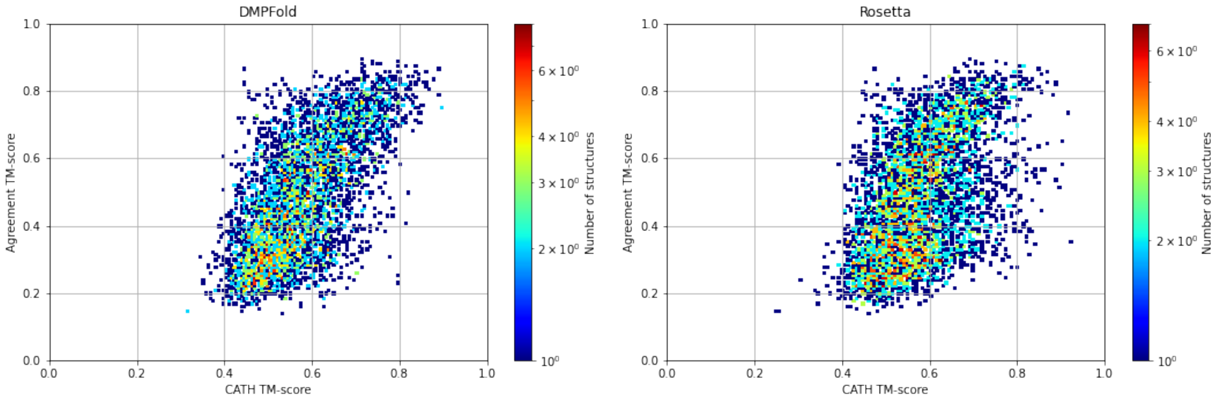

Supplementary Figure 27: A higher agreement TM-score between Rosetta and DMPFold models correlates with a higher TM-score to a structure in the CATH database. Since agreement TM-score correlates with MQA score as well, this Fig. extends the previous one.

9. Rosetta score

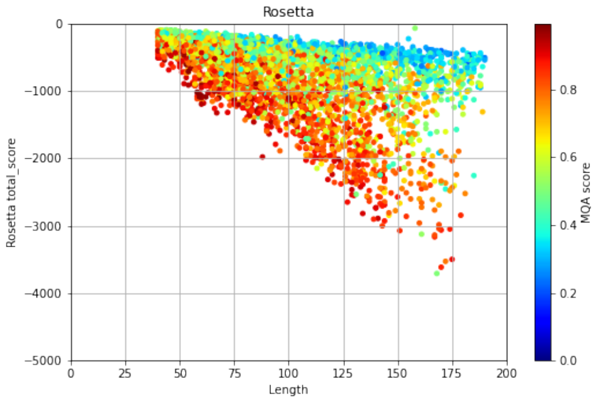

Supplementary Figure 28: The Rosetta total score decreases for larger protein models. This is expected because the score is a sum of the scores over all residues in the protein. Further, models with the lowest (i.e. best) Rosetta scores have the highest MQA scores, which is another advantage of the MQA score.

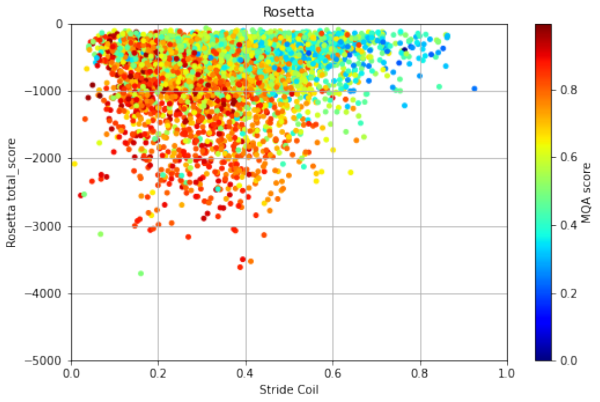

Supplementary Figure 29: The total Rosetta score generally gets worse (i.e. increases) with increasing coil content because of lack of hydrogen bonds, optimal solvation, etc.

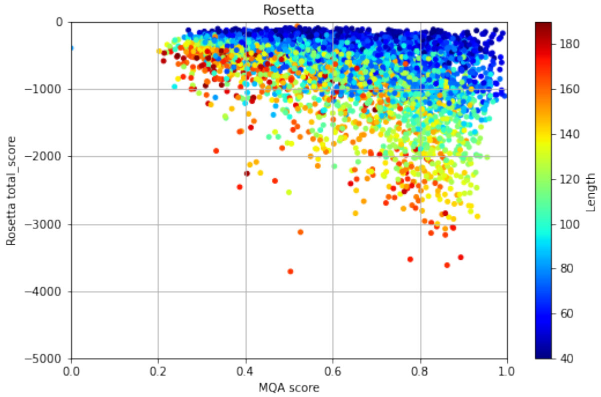

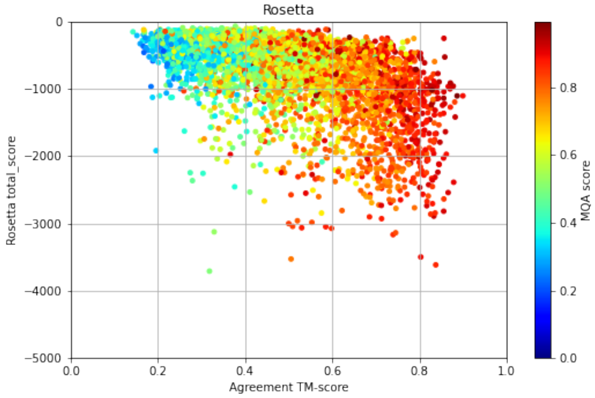

Supplementary Figure 30: Models with higher MQA scores can achieve lower Rosetta total scores, which are typically achieved by larger proteins.

Supplementary Figure 31: The more similar the DMPFold and Rosetta models are (high agreement TM-score), the higher the Rosetta MQA score and more likely that they can achieve a low (good) Rosetta score.

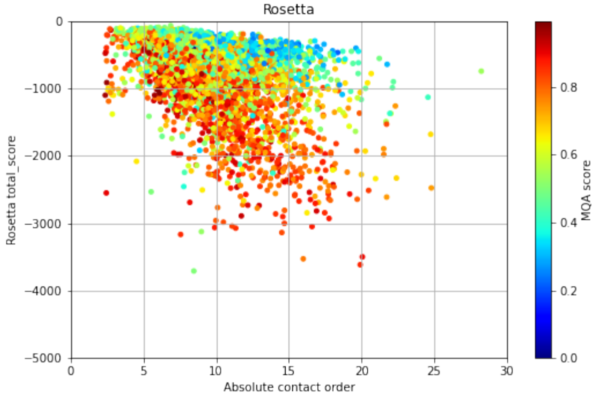

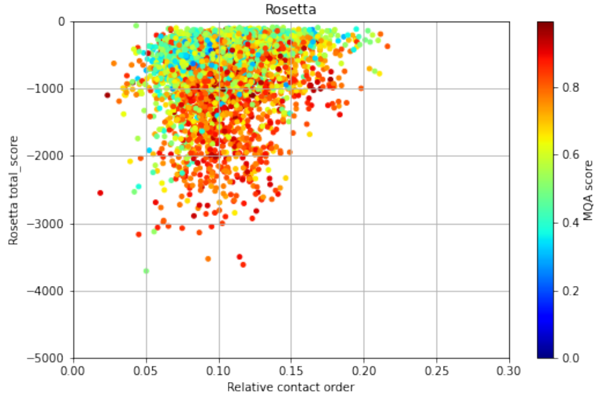

Supplementary Figure 32: The higher the absolute contact order (i.e. the larger the proteins are), the lower the Rosetta total score these models can achieve. The lower the Rosetta total score, the better the model quality, as shown by higher MQA scores.

Supplementary Figure 33: The correlation for the absolute contact order is washed out in the relative contact order, yet models on the higher end of the relative contact order typically have higher MQA scores and can have lower total Rosetta scores.

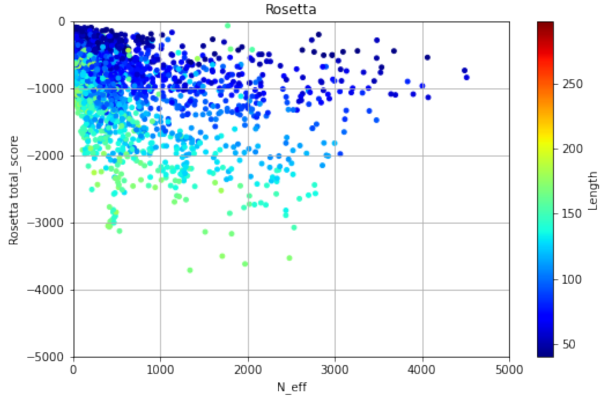

Supplementary Figure 34: For Rosetta, a higher N_eff leads to lower total Rosetta scores, indicating higher model quality. Further, this relationship also correlates with length – longer proteins have lower Rosetta scores,

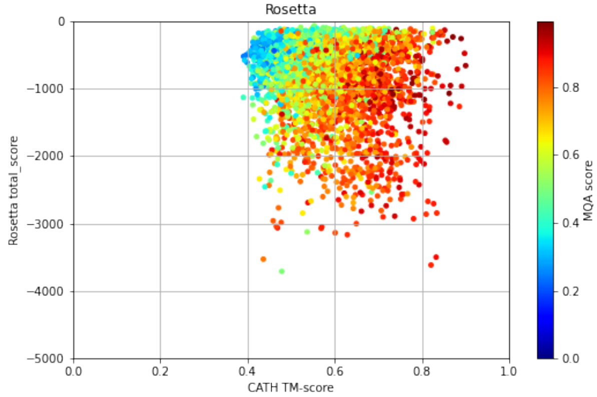

Supplementary Figure 35: Models with a higher TM-score to CATH can achieve lower Rosetta total scores (i.e. are better models) and typically also have a higher MQA score.

because the total Rosetta score is not normalized by protein length.

Supplementary Note 4: Additional results on the *MIP_curated* dataset

We performed a series of analyses including structure- and sequence-based annotations. The former include pairwise sequence and structural similarities (TM-align), function similarities (DeepFRI) and secondary structure assignments (Stride), which are already discussed in the main text and in this supplement (see MIP dataset curation, Novel folds and Sequence-structure-function relationships sections), and the contact order.

1. Transmembrane spans

Using the protein sequences, we predicted alpha-helical transmembrane spans with OCTOPUS and beta-strand transmembrane spans with BOCTOPUS (see methods in main paper). We discovered that the percentage of alpha-helical transmembrane spans in the common *MIP_curated* proteins is equal to 33%, which is according to expectations[25,26]. In all cases the transmembrane region is located at the center of the sequence and not at the termini. As a reference, the CATH 4.3.0 superfamily dataset comprises 6.8% of helical transmembrane proteins. Interestingly, the percentage of beta barrels in *MIP_curated* common (annotated by BOCTOPUS) is much lower, around 0.3%. In this case, although still below expectations (about 3% of proteins in gram-negative bacterial genomes[27]), the number is much closer to the reference CATH 4.3.0 superfamily dataset, where we also found ~0.3% of beta barrels.

2. Contact order

The contact order is a measure of fold complexity (see section Supplement section 6) and the relative contact order is the absolute contact order normalized by sequence length. Since our proteins are relatively small, their relative contact order is very similar to reference structures in CATH. We further observe that proteins with high beta-strand content have a larger relative contact order than alpha-helical ones (see Supplementary Figure 36).

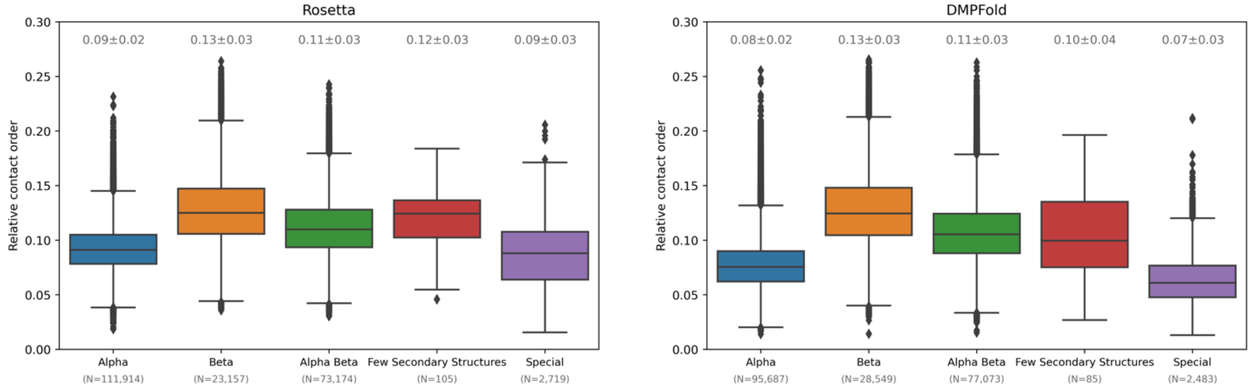

*Supplementary Figure 36: Box-and-whisker plots of the relative contact order for MIP_curated models stratified by CATH class. The box extends from Q1 to Q3 quartile values of the data, with a line at the median (Q2). The whiskers extend from the edges of box to show the range of the data. By Pandas default, they extend no more than 1.5 * inter-quartile range (IQR = Q3 - Q1) from the edges of the box, ending at the farthest data point within that interval. Outliers*

are plotted as separate dots. Above each box is the mean +/- stdev and the number of data points is shown below the x-axis.

3. Disorder

Intrinsically disordered regions (IDRs) are important parts of proteins but their behavior is still not well understood. About 2% of archean proteins, 4% of eubacterial, and 33% of eukaryotic proteins contain IDRs longer than 30 residues[28]. To identify IDRs in the *MIP_curated* dataset we used MobiDB-lite, which is a fast sequence-based method that, in the first step, uses 8 different predictors to derive a consensus and then filters for spurious short predictions in the second step. We found out that in the *MIP_raw* and curated datasets (considering common parts in both cases) the percentage of sequences containing at least one IDR is equal to 5.7 and 3.3 respectively, of which roughly 70% are located at the termini. The difference between raw and curated datasets is expected since the latter has less coil content and higher-quality models. However, we noticed that MobiDB-lite[19] is not sensitive enough in some cases, therefore for the putative novel folds dataset analysis we used DISOPRED3[20] (see *Novel folds* section below).

Supplementary Note 5: Novel folds

1. Novel fold identification: overall procedure

Novel folds were identified in a two-step procedure: First, we compared protein models in the *MIP_curated* dataset to CATH superfamilies using the TM-score from TMalign. All resulting models without significant matches, i.e. that had a TM-score < 0.5, were then compared against the PDB90, again using the TM-score from TMalign (see below for filtering procedure and cutoff justification). All models without matches in the PDB90 and which had a high agreement score between Rosetta and DMPFold models (agreement TM-score >= 0.5) were never-seen-before structures, of which were 452. These were then clustered into 161 novel folds (see below for clustering procedure) and verified through AlphaFold2, which identified 13 false positives, resulting in 438 novel structures clustered into 148 novel folds.

2. Novel fold identification: verifying a sensible TM-score cutoff

To choose and verify a sensible cutoff value for structural similarity using the TM-score, we computed the posterior probability that two folds within CATH 4.2.0 are similar (Supplementary Figure 37). It has been shown previously (see reference [29], Fig. 6) that a TM-score = 0.4 corresponds to at most 5% probability that two folds are similar. Further, according to Fig. 3 and equation 2 in the same paper[29], the p-value for observing similar folds at random at a TM-score = 0.4 is 3.54 x 10^-5.

Defining novel folds by a strict cut-off is debatable, but a 0.5 cut-off value is a meaningful choice given the data and is the most common definition used (see e.g. reference[30]). For 0.55 the probability of finding a known fold rises to 60-70% which still doesn't guarantee that a given fold is not novel. In these cases, a manual inspection of the models is required (see below).

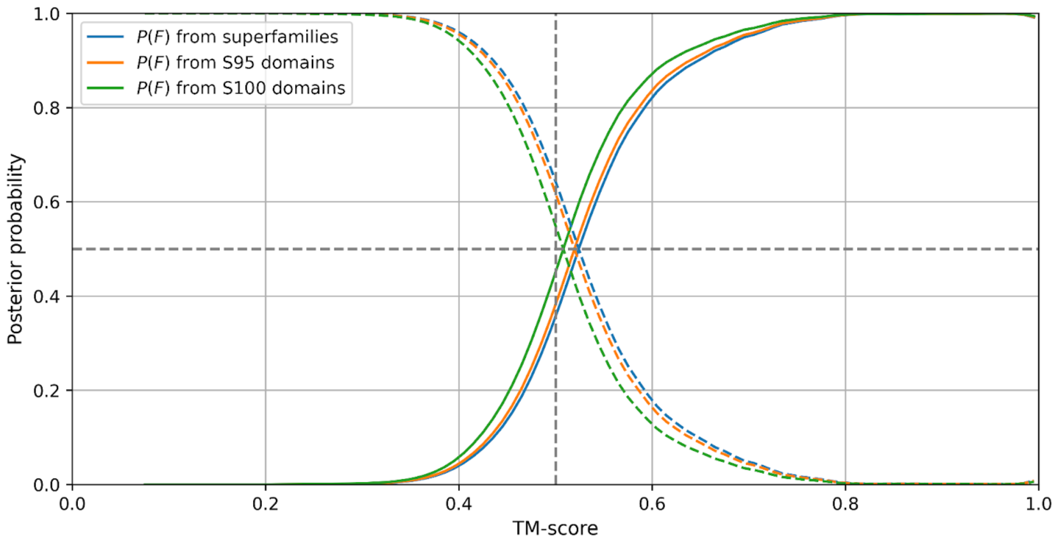

Supplementary Figure 37: Validating the TM-score cutoff by computing the posterior probability that two proteins in CATH 4.2.0 share the same fold, verifying that a TM-score cutoff of 0.5 is a sensible choice.

3. Novel fold identification: comparison against CATH and PDB90

The CATH filtering step is depicted in Supplementary Figure 38 and shows maximum TM-scores for all *MIP_curated* models for both Rosetta and DMFold. Models that survived are placed in the lower left quadrant. Interestingly, the PDB filtering step is much more restrictive for Rosetta predictions - see Supplementary Table 4. Coil content increases after each filtering step (see Supplementary Figure 40) but, in general, is still moderate. Such behavior is expected, since higher coil content generally translates into lower agreement with experimental (well-folded) structures deposited in CATH/PDB. The coil content is usually lower for Rosetta predictions which are constructed from fragments, in contrast to DMPFold. However, the coil content in our novel fold examples is relatively low (~30% on average).

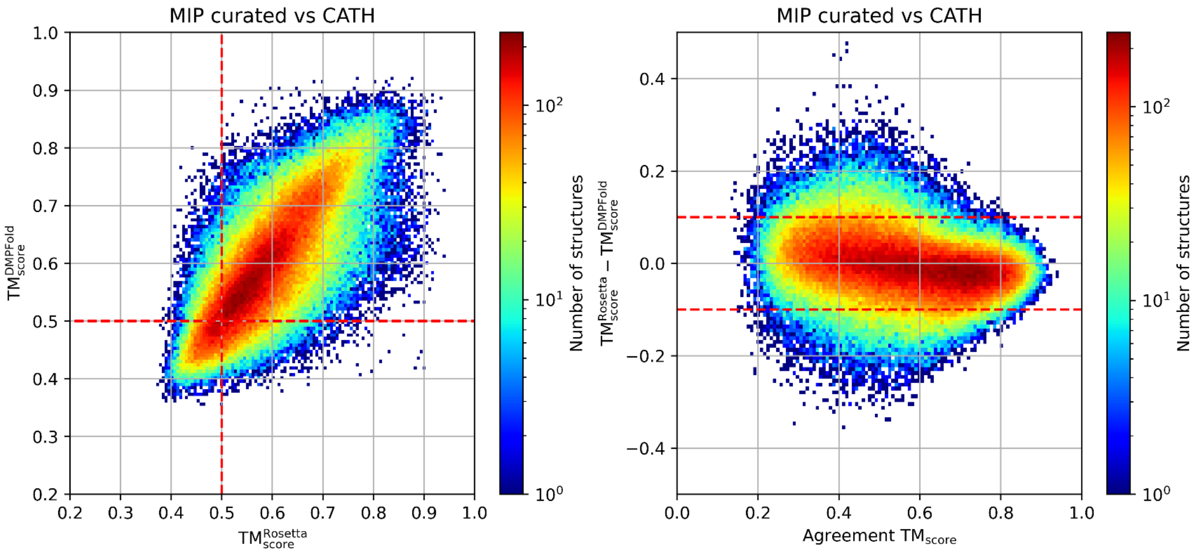

Supplementary Figure 38: CATH filtering step. Left: number of structures in MIP_curated with a given maximum TM-score between Rosetta/DMPFold (shown on axes) and CATH. Only the models in the lower left quadrant (dotted red lines) were compared against the PDB90 in a second step. Right: agreement TM-score between Rosetta and DMPFold vs the difference between TM-score values depicted in the left panel.

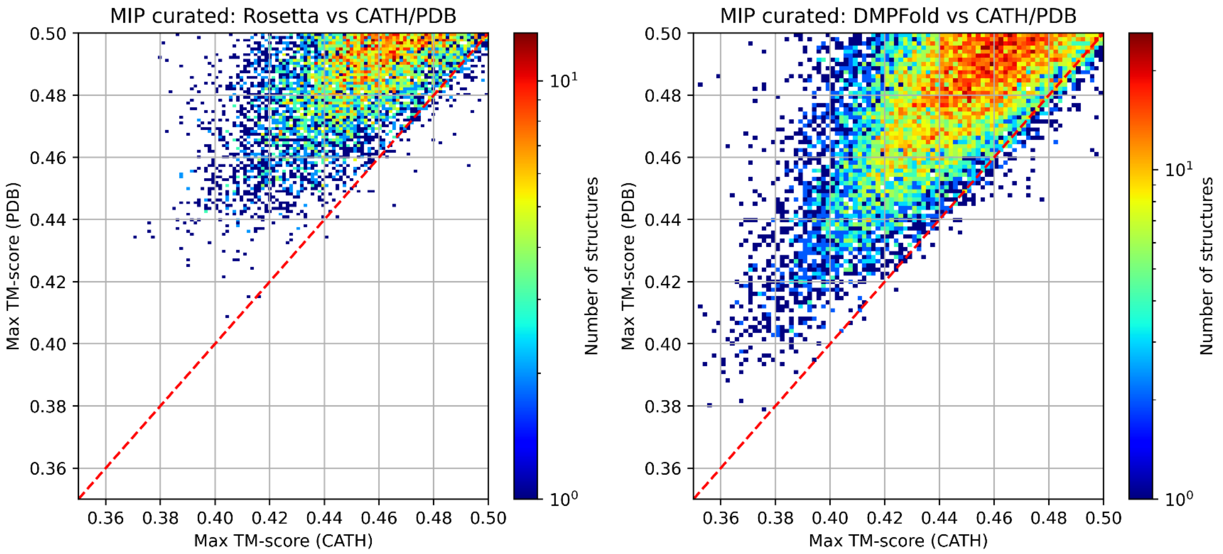

Supplementary Figure 39: PDB vs CATH filtering step. Number of structures in MIP_curated (left: Rosetta, right: DMPFold) with a given maximum TM-score against CATH (horizontal axis) and PDB (vertical axis). Note that the TM-scores are <0.5 indicating low agreement between the predicted models and CATH and PDB.

Supplementary Table 4: Number of *MIP_curated* models that survived after CATH and PDB filtering steps*

	All	# of models after CATH filtering	# of models after PDB filtering
**Rosetta curated**	211,069	29,624	7,434
**DMFPold curated**	203,877	40,079	14,294
**Common**	184,642	16,175	2,751
**Common (TM-score >= 0.5)**			**452**

**Note that about twice as many DMPFold models survived after both filtering steps, than Rosetta models, which likely originates in the facts that DMPFold models have a higher quality for larger proteins as compared to Rosetta and that larger proteins are less likely to have a match in CATH or the PDB due to the increased conformational space it occupies. Further, DMPFold models have a higher coil content than Rosetta models (see Supplementary Figure 5), thereby increasing the conformational space and decreasing the likelihood of a match to CATH or the PDB. The number of putative novel folds is in the last row and last column.*

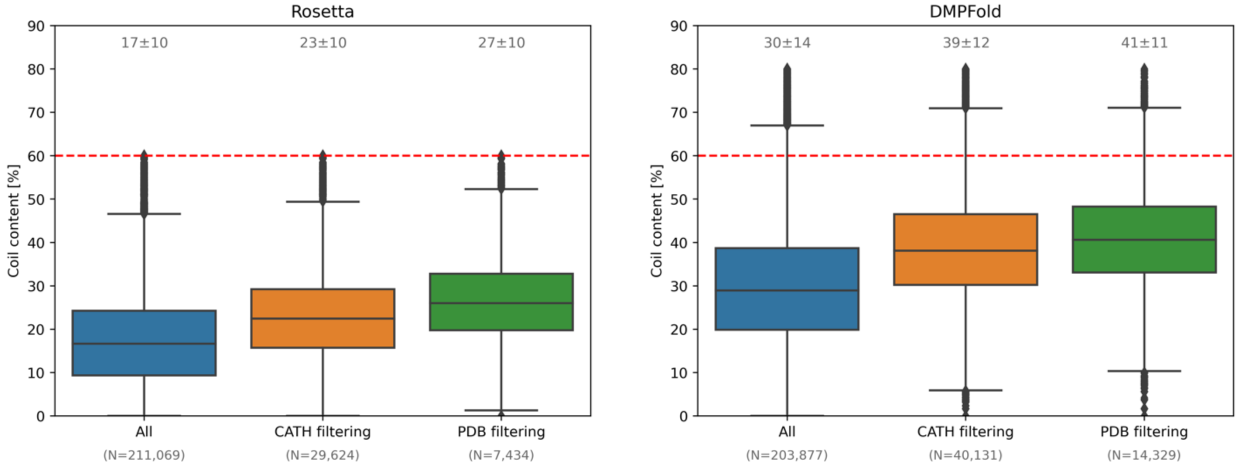

*Supplementary Figure 40: Box-and-whisker plots of the coil content in MIP_curated after CATH and PDB filtering steps. DMPFold models generally have a higher coil content than Rosetta models. The box extends from Q1 to Q3 quartile values of the data, with a line at the median (Q2). The whiskers extend from the edges of box to show the range of the data. By Pandas default, they extend no more than 1.5 * inter-quartile range (IQR = Q3 - Q1) from the edges of the box, ending at the farthest data point within that interval. Outliers are plotted as separate dots. Above each box is the mean +/- stdev and the number of data points is shown below the x-axis.*

4. Clustering procedure to identify novel fold clusters

We identified novel fold clusters within the set of 452 putative novel folds as follows - the Rosetta and DMPFold datasets were handled separately unless otherwise noted:

1. The 452 novel fold predictions from Rosetta (or DMPFold) were superimposed against each other to compute pairwise TM-scores, resulting in `N*(N-1)/2` comparisons. In each dataset (Rosetta or DMPFold) we found similar models with a TM-score >= 0.5

(normalization by query or target sequence length is not relevant since the comparisons are symmetric).
2. We used the TM-score comparisons as a list of edges and created graphs using the Python package NetworkX. Node positions were computed using the Fruchterman-Reingold force-directed algorithm whereas connected components (clusters) were computed using the BFS (Breadth-first search) algorithm.
3. From the two datasets (Rosetta/DMPFold) we took the intersection between the two sets as the final set of novel folds.

Step 1 is summarized in Algorithm S1 - note that edge values i.e. node distances are computed as `1 - tmscore(model_1, model_2)`.

- As a result, the 452 putative novel folds were clustered into 161 novel folds
    - 7 are large clusters (with size 10 to 87)
    - 40 are medium-size clusters (with size 2-9)
    - 114 are singletons
- After discarding 6 hard and 8 soft false positives through AlphaFold2 verification (see section below) we are left with 438 putative novel folds and 148 common clusters where
    - 7 are large clusters (with size 10 to 87)
    - 39 are medium-size clusters (with size 2-9)
    - 102 are singletons

Algorithm S1: Construction of Rosetta and DMPFold edges used as input in NetworkX

```
for dataset in [Rosetta, DMPFold]
    edges[dataset] = []
    # run through all models
    for model_1 in dataset
        similar = []
        # filter out similar models
        for model_2 in dataset
            if tmscore(model_1, model_2) >= 0.5
                similar.add(model_2)
            end if
        end for
        # fill in the `edges` lists
        for model_2 in similar
            distance = 1 - tmscore(model_1, model_2)
            edges[dataset].add([model_1, model_2, distance])
        end for
    end for
end for
```

5. AlphaFold2 verification of putative novel folds

We ran AlphaFold2 on all of 452 putative novel fold sequences. Supplementary Figure 40 compares Rosetta, DMPFold and AlphaFold2 predictions. The majority of our predictions agree well with AlphaFold2. Interestingly, the mean agreement TM-scores between Rosetta/AlphaFold2 and DMPFold/AlphaFold2 are higher than the one between Rosetta and DMPFold which may indicate that AlphaFold2 predictions possess some features of both Rosetta and DMPFold models.

Moreover, the agreement TM-score between Rosetta/AlphaFold2 and DMPFold/AlphaFold2 models correlates well with Rosetta/DMPFold MQA scores (see Supplementary Figure 42). We also see a slightly weaker correlation between the mean pLDDT score (i.e. AlphaFold2 MQA score) and Rosetta/DMPFold MQA scores (see Supplementary Figure 43).

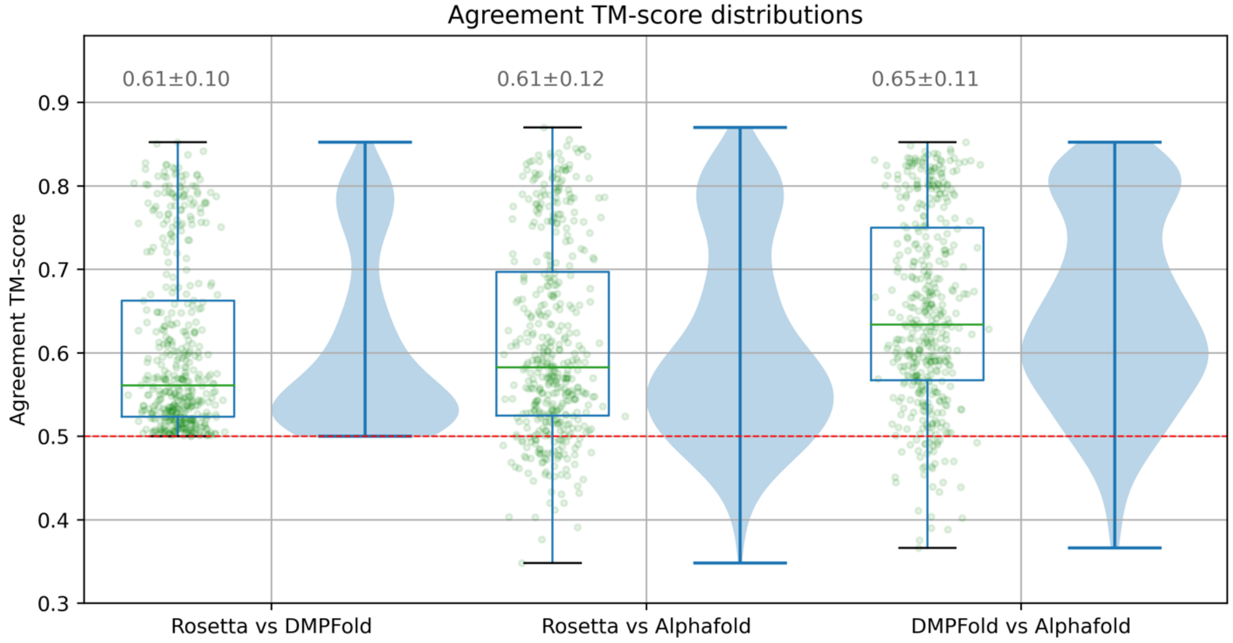

*Supplementary Figure 41: Comparison between Rosetta, DMPFold and AlphaFold2 putative novel fold predictions, showns as box-and-whisker plots. Points correspond to agreement TM-score between two given methods. Note the scale on the y-axis doesn't cover the full range of TM-scores from 0 to 1. The box extends from Q1 to Q3 quartile values of the data, with a line at the median (Q2). The whiskers extend from the edges of box to show the range of the data. By Pandas default, they extend no more than 1.5 * inter-quartile range (IQR = Q3 - Q1) from the edges of the box, ending at the farthest data point within that interval. Outliers are plotted as separate dots. Above each box is the mean +/- stdev and the number of data points is N = 452.*

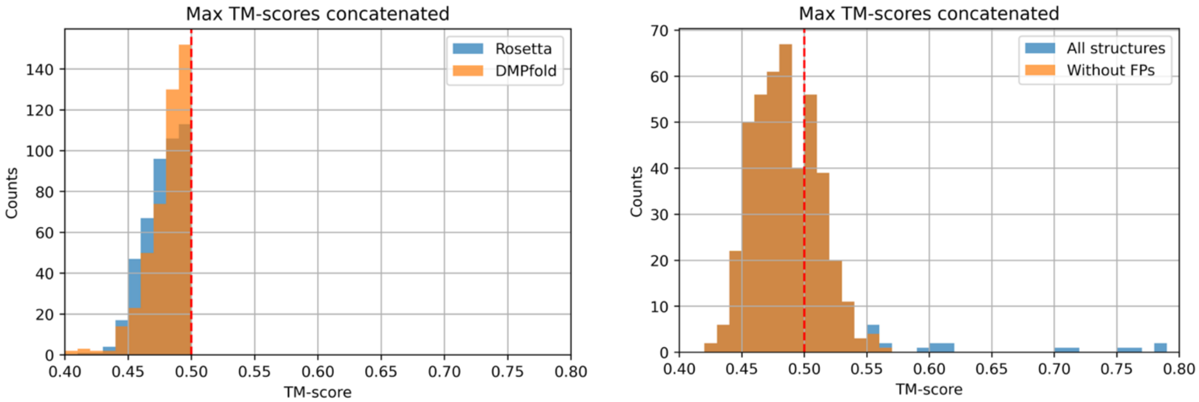

Supplementary Figure 42: Distribution of maximum TM-scores against PDB from putative novel folds dataset (see Supplementary Table 5) for Rosetta and DMPFold (left) and AlphaFold predictions (right).

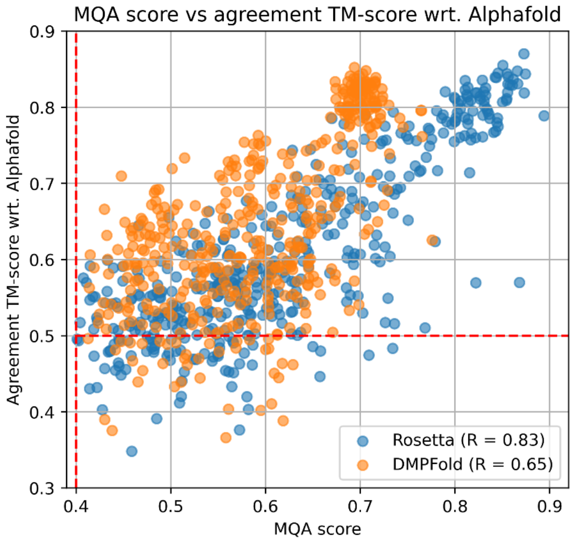

Supplementary Figure 43: MQA score vs agreement TM-score between Rosetta/DMPFold and AlphaFold2. All structures come from the putative novel fold dataset. Pearson R correlation coefficients are shown in the legends.

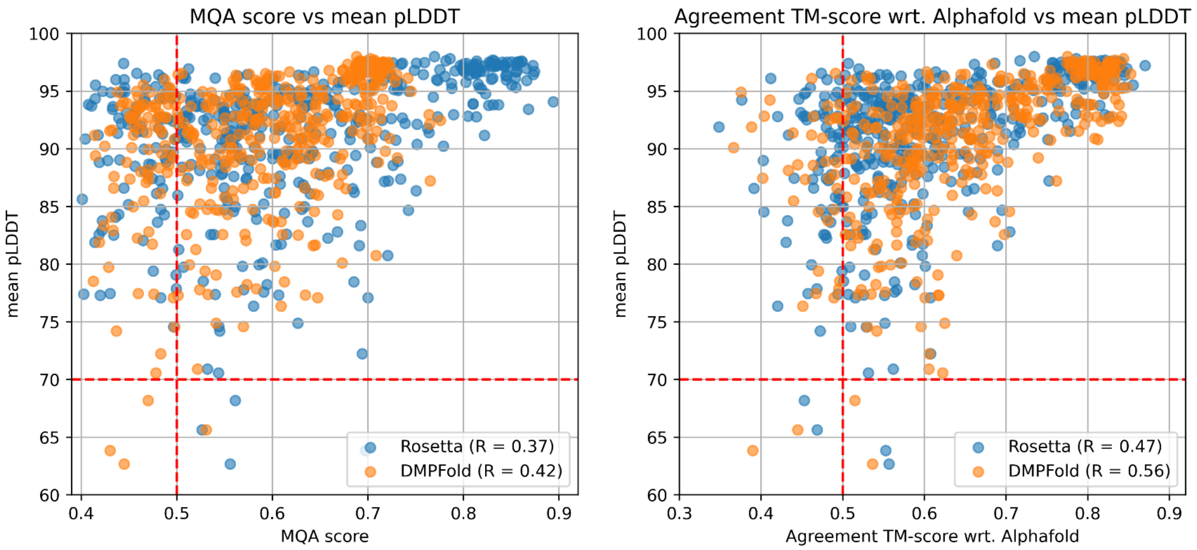

Supplementary Figure 44: Left: MQA score for Rosetta and DMPFold vs mean pLDDT (local accuracy of AlphaFold2 predictions). Right: agreement TM-score between Rosetta/AlphaFold2 and DMPFold/AlphaFold2 vs mean pLDDT. All models come from the putative novel fold dataset. Pearson R correlation coefficients are shown in the legends.

Potential false positives

For the majority of AlphaFold2 novel fold models the maximum TM-score against CATH and PDB is below the threshold of 0.5 (see Supplementary Figure 45). However, there are some models for which similar structures are already deposited in the PDB. We decided not to discard models just above the threshold (~0.5-0.55) since such predictions should be verified experimentally. For the remainder, we distinguished two types of potential false positives (see Supplementary Figure 45):

- Hard false positives - total of 6 models:
  - Maximum TM-score between AlphaFold2 model and PDB >= 0.7
- Soft false positives (SFP) - total of 8 models:
  - maximum TM-score between AlphaFold2 model and PDB >= 0.58 (slightly below 0.6 - see Supplementary Figure 45)
  - **or** maximum TM-score between AlphaFold2 model and CATH >= 0.55
  - **minus** Hard False Positive above

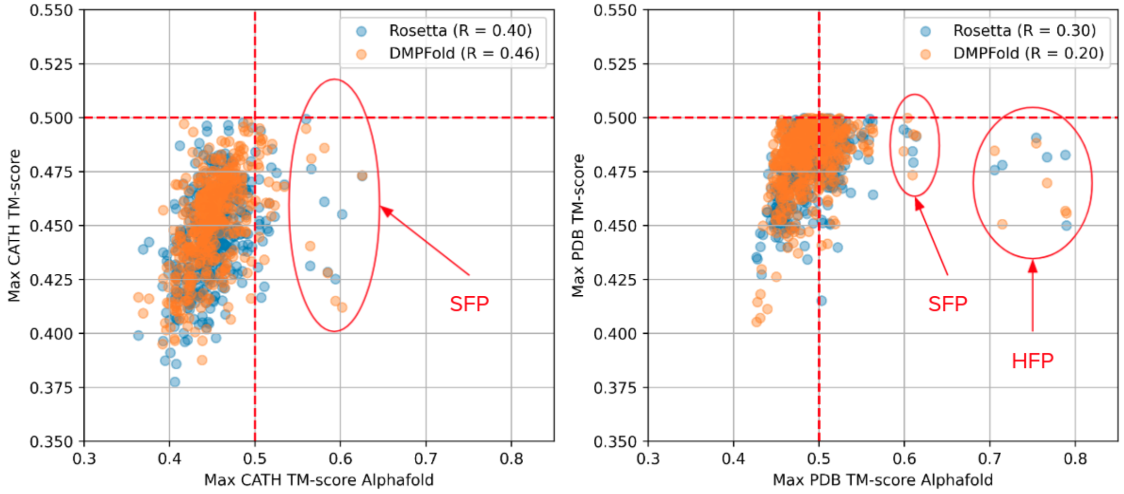

Supplementary Figure 45: Left: maximum TM-score between AlphaFold2 and CATH (horizontal axis) and between Rosetta/DMPFold and CATH (vertical axis). Right: maximum TM-score between AlphaFold2 and PDB (horizontal axis) and between Rosetta/DMPFold and PDB (vertical axis). All structures come from the putative novel fold dataset. Pearson R correlation coefficients are shown in the legends.

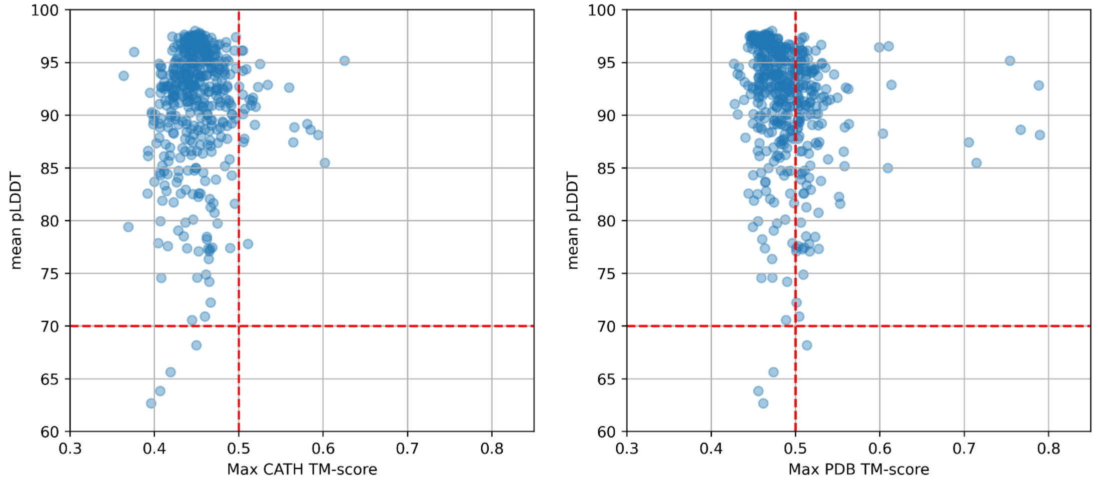

Supplementary Figure 46: maximum TM-score between AlphaFold2 and CATH (left)/PDB (right) vs mean pLDDT.

Supplementary Table 6: Hard false positives

	Agreement TM-score vs Rosetta	Agreement TM-score vs DMPFold	CATH homologous superfamily number	CATH TM-score (MIP-normed)	PDB90 domain name	PDB90 TM-score (MIP-normed)	DISOPRED 3 percentage	mean pLDDT	Cluster ID
MIP_00152462	0.525	0.440	2x8kC01	0.602	5w5fA	0.714	3.4	85.5	38
MIP_00201772	0.470	0.433	4hkhA00	0.594	6j0nn	0.789	0.0	88.1	96
MIP_00074380	0.454	0.404	3vkiA02	0.425	3teeA	0.788	7.6	92.8	87
MIP_00220620	0.528	0.445	2x8kC01	0.585	6j0nb	0.767	0.0	88.6	100
MIP_00297536	0.480	0.483	4hkhA00	0.565	6xgrA	0.705	4.2	87.4	29
MIP_00196377	0.517	0.537	2qzbA00	0.625	4ifaA	0.754	0.0	95.2	62

Supplementary Table 7: Soft false positives

	Agreement TM-score vs Rosetta	Agreement TM-score vs DMPFold	CATH homologous superfamily number	CATH TM-score (MIP-normed)	PDB90 domain name	PDB90 TM-score (MIP-normed)	DISOPRED 3 percentage	mean pLDDT	Cluster ID
MIP_00280029	0.554	0.585	3h0nA00	0.560	3h0nA	0.560	0.7	92.6	32
MIP_00227385	0.501	0.532	3ar4A02	0.449	6a2uA	0.609	18.6	85.0	117
MIP_00253881	0.558	0.586	4fczA00	0.566	5uwbB	0.558	10.1	88.9	5
MIP_00266643	0.551	0.690	2z0rC00	0.581	2z0rA	0.563	0.0	89.2	77
MIP_00251287	0.545	0.645	5gzkA00	0.423	6y56A	0.599	0.0	96.5	11
MIP_00309094	0.467	0.502	5o6hA00	0.534	5kf2A	0.614	1.8	92.9	84
MIP_00054198	0.474	0.491	1c5eA00	0.462	5wk1X	0.610	0.0	96.5	58
MIP_00246175	0.521	0.596	5b1aA00	0.423	6a2uA	0.603	17.4	88.3	117

All hard and soft false positives are singletons in our novel fold clusters. Two SFPs (MIP_00227385, MIP_00246175) belong to the same cluster (cluster 117). The mean pLDDT for those AlphaFold2 predictions is generally >= 0.85, so the confidence in these predictions is pretty high (see Supplementary Figure 46). Therefore, we are more convinced that they are indeed potential false positives and we decided to remove them from the final putative novel fold dataset.

A note on fragments, templates and what the prediction methods "know"

Rosetta does not directly use templates but it uses fragments for model building. The fragments come from the *VALL* database which was last updated around 2011. Contacts for Rosetta are predicted by GREMLIN which uses multiple sequence alignments generated from public databases, which should have been trained around 2016/7.

DMPFold uses neural networks trained on public databases before 2019, so it would not have seen anything after that time.

AlphaFold2, on the other hand, downloads the entire PDB during installation or setup, to use as templates. We downloaded the PDB on July 31, 2021, so AlphaFold2 would have seen every one of the hard false positive example structures in the PDB.

Hard false positive examples

Below we show and briefly describe all hard false positive examples.

Hard False Positive #1

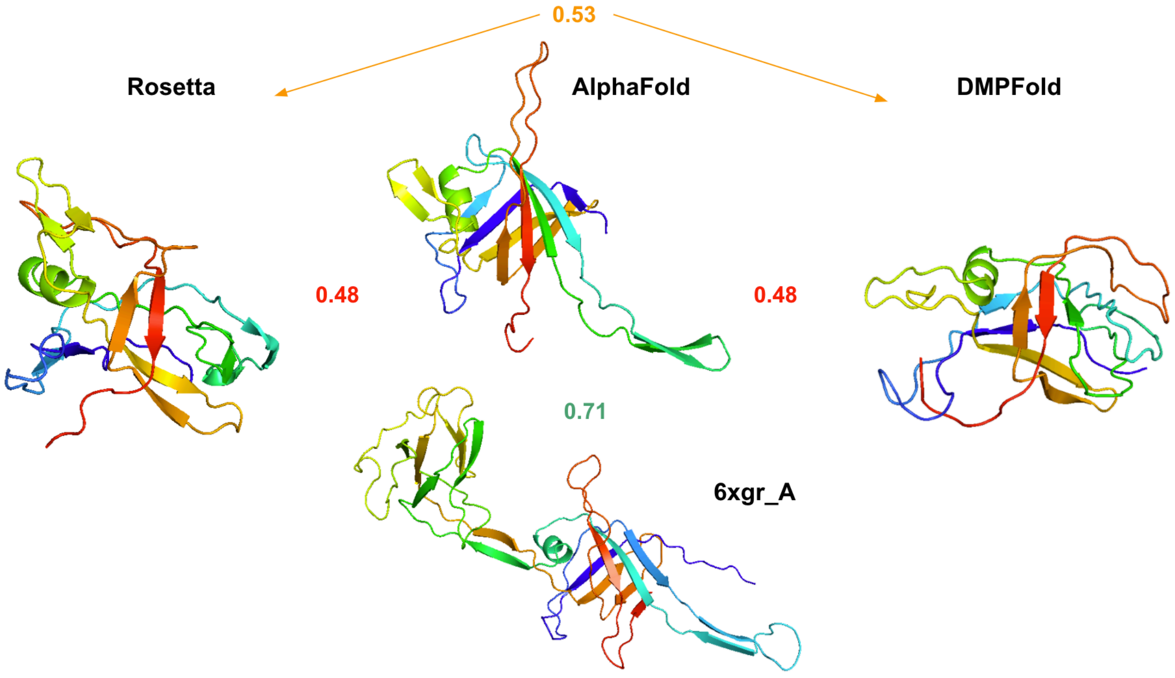

	Rosetta	AlphaFold	DMPFold
**Max CATH TM-score:**	0.43	0.56	0.44
**Max PDB TM-score:**	0.48	0.71	0.48
**MQA score / Coil content:**	0.47 / 28%	0.87 / 26%	0.6 / 27%

Supplementary Figure 47: MIP_00297536 structure comparison between Rosetta, AlphaFold2 and DMPFold (upper models) and the most similar PDB to the AlphaFold2 prediction (lower structure). Mutual TM-scores are shown in between. Additional parameters are listed in the table.

- Most similar PDB90 structure for all models is 6xgr_A (**Deposited:** 2020-06-17 **Released:** 2020-07-01)
- very high agreement of PDB structure against AlphaFold2 model is due to the tail position (similar tail might be found in two templates used by AlphaFold2 for model prediction: 6nbx_N Deposited: 2018-12-10 Released: 2019-02-27; 6nby_N Deposited: 2018-12-10 Released: 2019-02-27)
- Tail fragment predicted by AlphaFold2 with mean pLDDT around 0.77 (confident)
- pTM (global score) for AlphaFold2: 0.77 (confident)

Hard False Positive #2

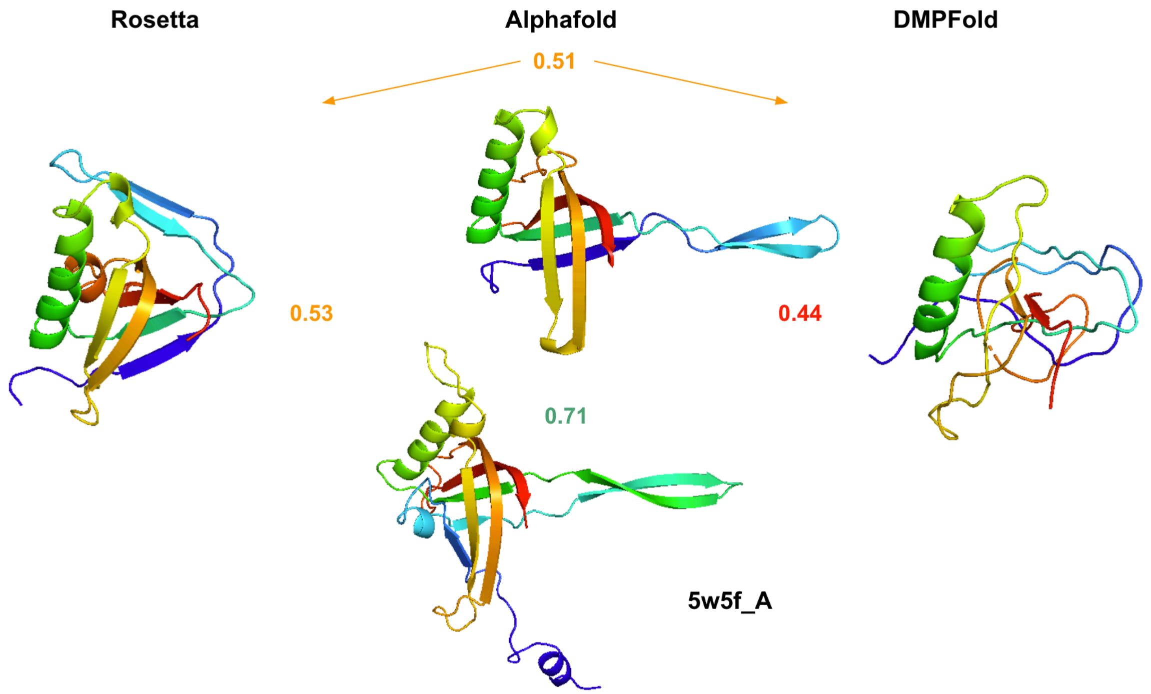

	Rosetta	Alphafold	DMPFold
**Max CATH TM-score:**	0.46	0.6	0.41
**Max PDB TM-score:**	0.48	0.71	0.45
**MQA score / Coil content:**	0.55 / 25%	0.85 / 20%	0.47 / 32%

Supplementary Figure 48: MIP_00152462 structure comparison between Rosetta, AlphaFold2 and DMPFold (upper models) and the most similar PDB to the AlphaFold2 prediction (lower structure). Mutual TM-scores are shown in between. Additional parameters are listed in the table.

- All AlphaFold2 templates released in PDB database before 2019
- Most similar PDB structure for AlphaFold2 model: 5w5f_A (**Deposited:** 2017-06-15 **Released:** 2017-08-16)
- very good agreement between Rosetta and AlphaFold2 predictions except for the tail fragment
- tail fragment predicted by AlphaFold2 with mean pLDDT around 0.82 (confident)
- pTM (global score) for AlphaFold2: 0.76

Hard False Positive #3

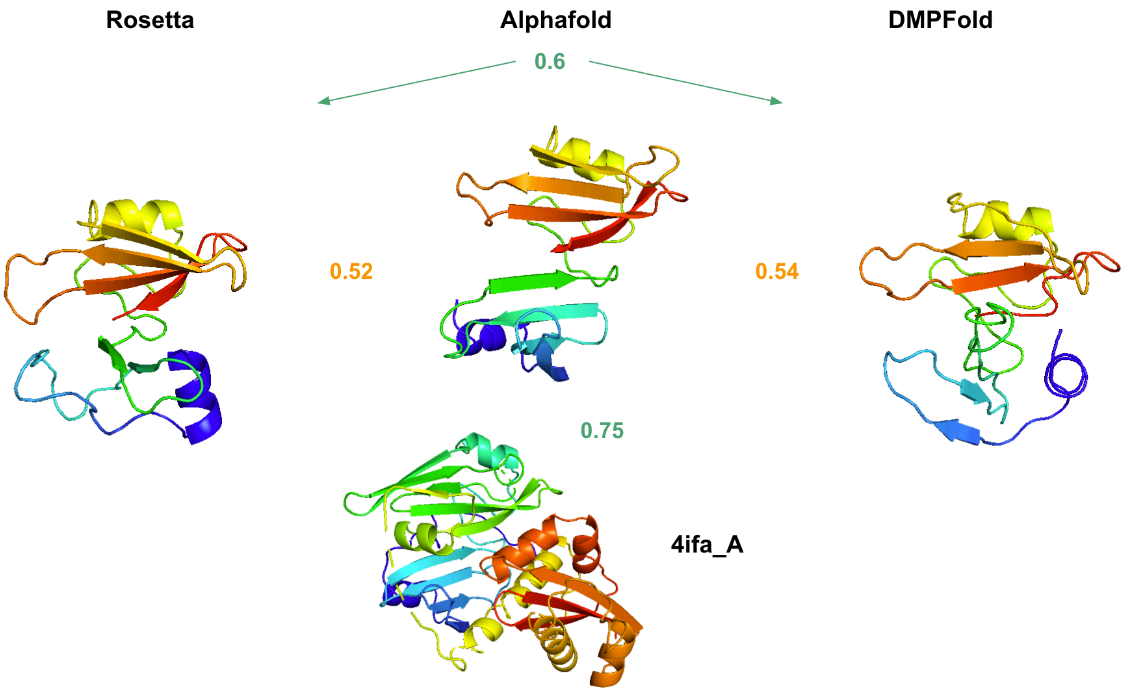

	Rosetta	Alphafold	DMPFold
**Max CATH TM-score:**	0.47	0.63	0.47
**Max PDB TM-score:**	0.49	0.75	0.49
**MQA score / Coil content:**	0.62 / 32%	0.95 / 34%	0.49 / 44%

Supplementary Figure 49: MIP_00196377 structure comparison between Rosetta, AlphaFold2 and DMPFold (upper models) and the most similar PDB to the AlphaFold2 prediction (lower structure). Mutual TM-scores are shown in between. Additional parameters are listed in the table.

- All AlphaFold2 templates released in PDB database before 2019
- Most similar PDB structure for AlphaFold2 model: 4ifa_A (**Deposited:** 2012-12-14 **Released:** 2012-12-26)
- AlphaFold2 prediction has high symmetry which is the reason for high agreement to 4ifa_A
- No disorder, very confident AlphaFold2 predictions
- pTM (global score) for AlphaFold2: 0.86

Hard False Positive #4

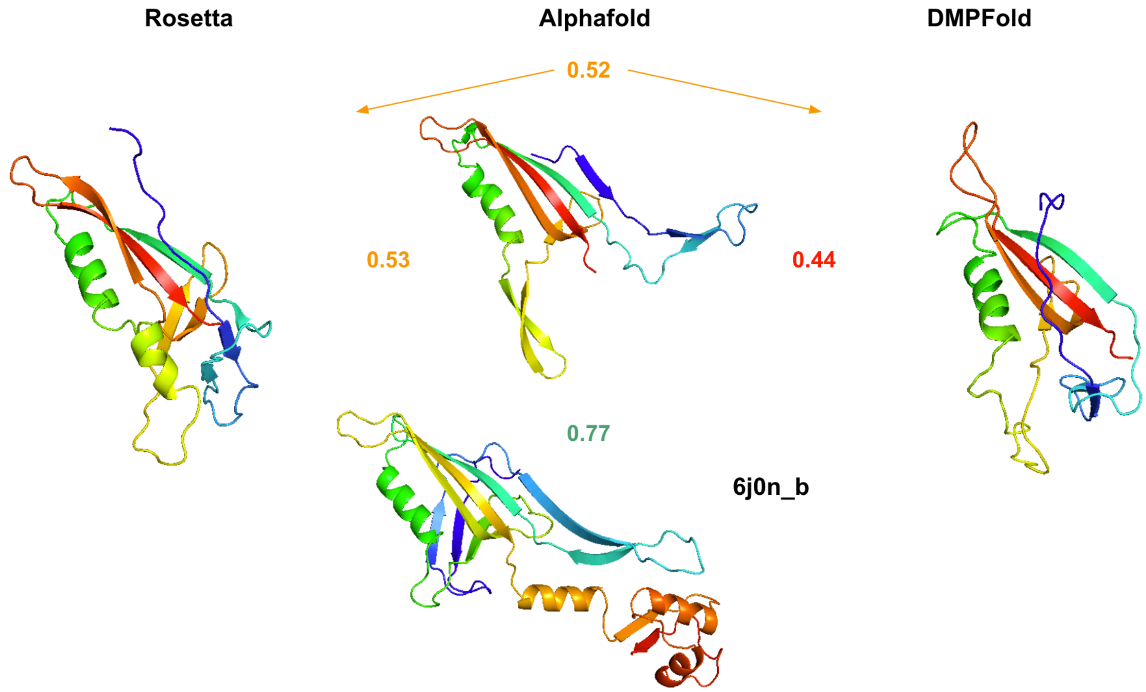

	Rosetta	Alphafold	DMPFold
**Max CATH TM-score:**	0.43	0.58	0.43
**Max PDB TM-score:**	0.48	0.77	0.47
**MQA score / Coil content:**	0.47 / 23%	0.89 / 19%	0.61 / 35%

Supplementary Figure 50: MIP_00220620 structure comparison between Rosetta, AlphaFold2 and DMPFold (upper models) and the most similar PDB to the AlphaFold2 prediction (lower structure). Mutual TM-scores are shown in between. Additional parameters are listed in the table.

- Most similar PDB structure for AlphaFold2 model: 6j0n_b (**Deposited:** 2018-12-25 **Released:** 2019-04-10)
- However, among templates used by AlphaFold2 we can find: 6rao_G Deposited: 2019-04-06 Released: 2019-04-17; 6j0n_d Deposited: 2018-12-25 Released: 2019-04-10
- Similarly to *Hard False Positive #1* and *Hard False Positive #2*, high agreement with PDB is due to matching tail fragments
- DISOPRED (sequence-based) predicts that blue/yellow tail fragments weakly disordered up to 40%/20% (17%/13% on average)
- However, they are predicted by AlphaFold2 with mean pLDDT around 0.77 (confident)
- pTM (global score) for AlphaFold2: 0.76

Hard False Positive #5

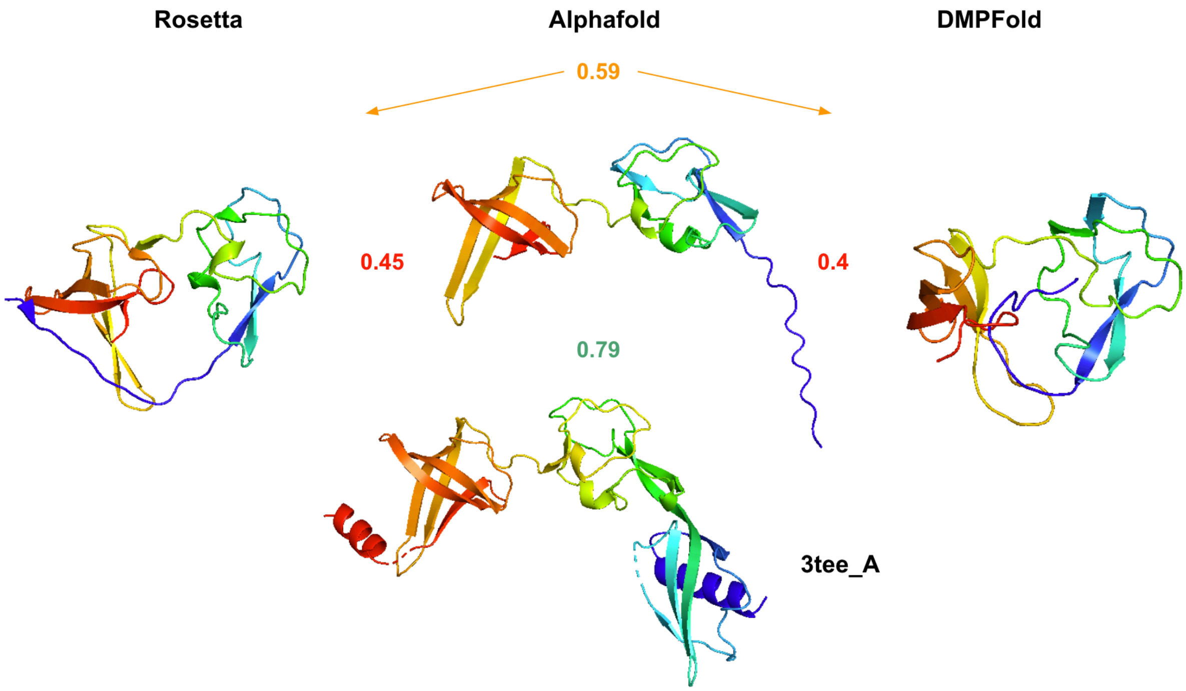

	Rosetta	Alphafold	DMPFold
**Max CATH TM-score:**	0.41	0.43	0.42
**Max PDB TM-score:**	0.48	**0.79**	0.46
**MQA score / Coil content:**	0.56 / 32%	0.93 / 39%	0.56 / 32%

Supplementary Figure 51: MIP_00074380 structure comparison between Rosetta, AlphaFold2 and DMPFold (upper models) and the most similar PDB to the AlphaFold2 prediction (lower structure). Mutual TM-scores are shown in between. Additional parameters are listed in the table.

- the most similar PDB structure (3tee_A) was also used by AlphaFold2 as a template:
  - 3tee_A - Deposited: 2011-08-12 Released: 2012-08-15
- However, the alignment is not perfect due to disordered N-terminus (65% on average) which doesn’t match well with the template (pLDDT for that fragment is around 0.63)
- pTM (global score) for AlphaFold2: 0.71

Hard False Positive #6

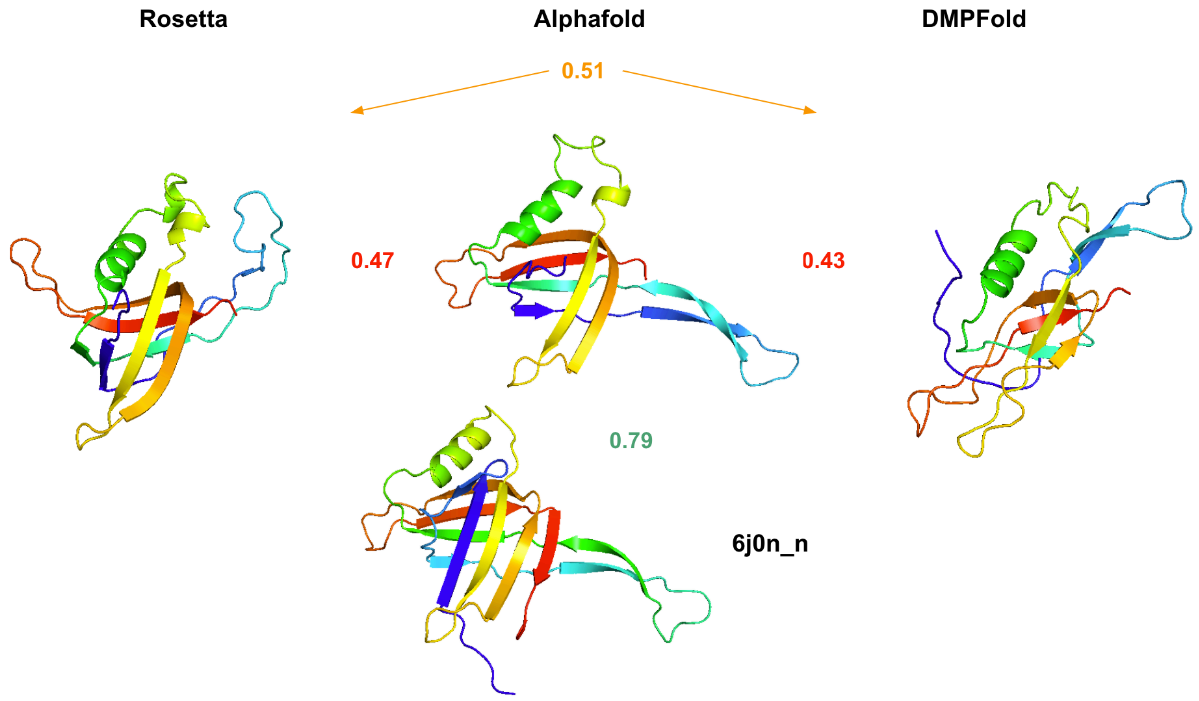

	Rosetta	Alphafold	DMPFold
**Max CATH TM-score:**	0.43	0.59	0.42
**Max PDB TM-score:**	0.45	0.79	0.46
**MQA score / Coil content:**	0.52 / 26%	0.88 / 22%	0.5 / 26%

Supplementary Figure 52: MIP_00201772 structure comparison between Rosetta, AlphaFold2 and DMPFold (upper models) and the most similar PDB to the AlphaFold2 prediction (lower structure). Mutual TM-scores are shown in between. Additional parameters are listed in the table.

- Most similar PDB structure for AlphaFold2: 6j0n_n (**Deposited:** 2018-12-25 **Released:** 2019-04-10)
- this structure (different chain name) is also present among templates used by AlphaFold2 (not all templates listed below):
  - 6rao_F Deposited: 2019-04-06 Released: 2019-04-17
  - 6j0n_r Deposited: 2018-12-25 Released: 2019-04-10
  - 6rbn_B Deposited: 2019-04-11 Released: 2019-04-24
  - 6rap_A Deposited: 2019-04-07 Released: 2019-04-17
- The prediction of the tail fragment (mean pLDDT around 94%) agrees perfectly with the template; there are however some subtle differences in the middle part (yellow color)
- No disordered fragments
- pTM (global score) for AlphaFold2: 0.78

A similar trend is observed for Soft False Positives (SFPs) i.e. AlphaFold2 predictions are generally more similar to structures in the PDB, likely because it used them as templates. However, the TM-scores between all three predictions is higher (with mean around 0.55 as compared to 0.5 for Hard False Positives). Moreover, mean pLDDT and pTM for AlphaFold2 predictions are larger: 0.8 and 0.7 respectively.

6. Novel fold examples

Below we describe a few examples of our predicted novel folds verified by AlphaFold2.

Novel fold example #1

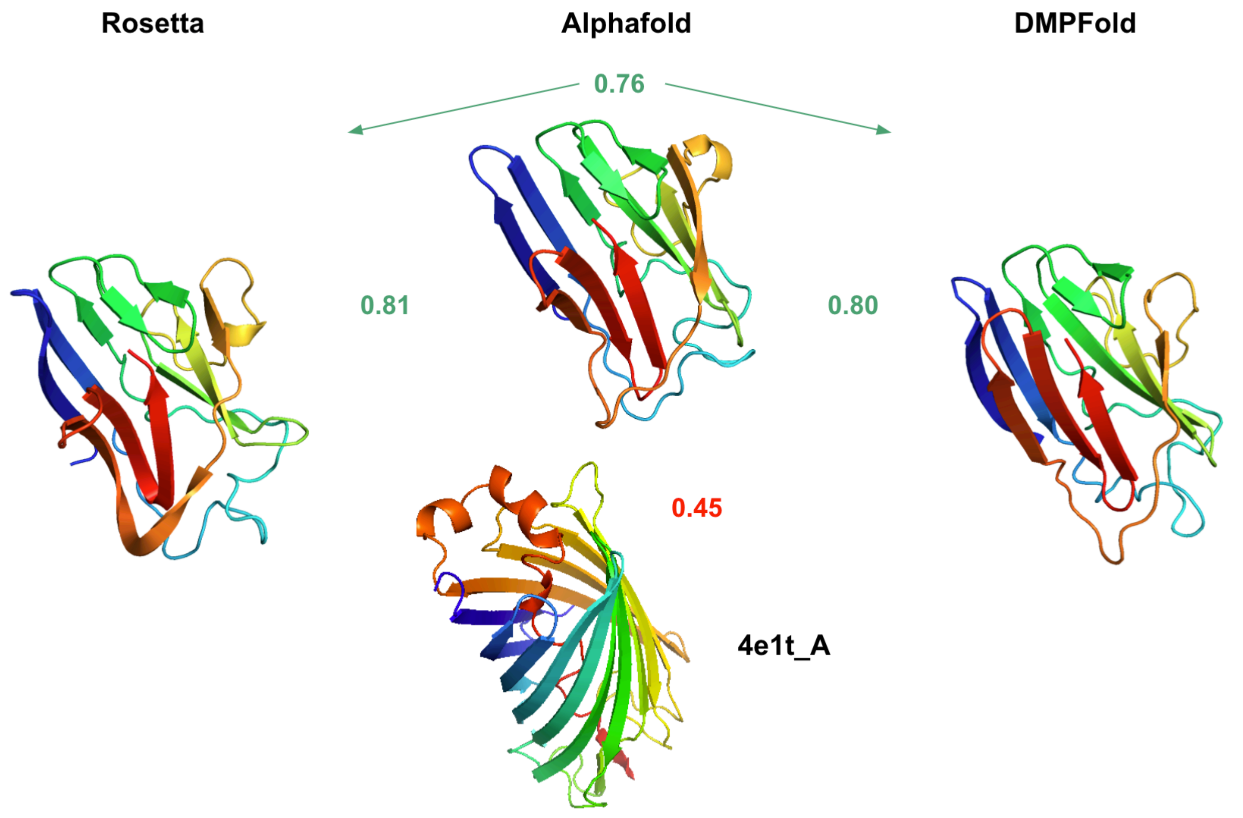

	Rosetta	Alphafold	DMPFold
**Max CATH TM-score:**	0.44	0.44	0.45
**Max PDB TM-score:**	0.46	0.45	0.49
**MQA score / Coil content:**	0.80 / 30%	0.97 / 24%	0.71 / 31%

Supplementary Figure 53: MIP_00261827 (cluster 161) structure comparison between Rosetta, AlphaFold2 and DMPFold (upper models) and the most similar PDB to the AlphaFold2 prediction (lower structure). Mutual TM-scores are shown in between. Additional parameters are listed in the table.

- this example shows the models in the largest cluster with 87 representatives
- Excellent agreement between all methods
- All max TM-scores against CATH and PDB (including AlphaFold2) below 0.5
- No disorder
- pTM (global score) for AlphaFold2: 0.90

Novel fold example #2

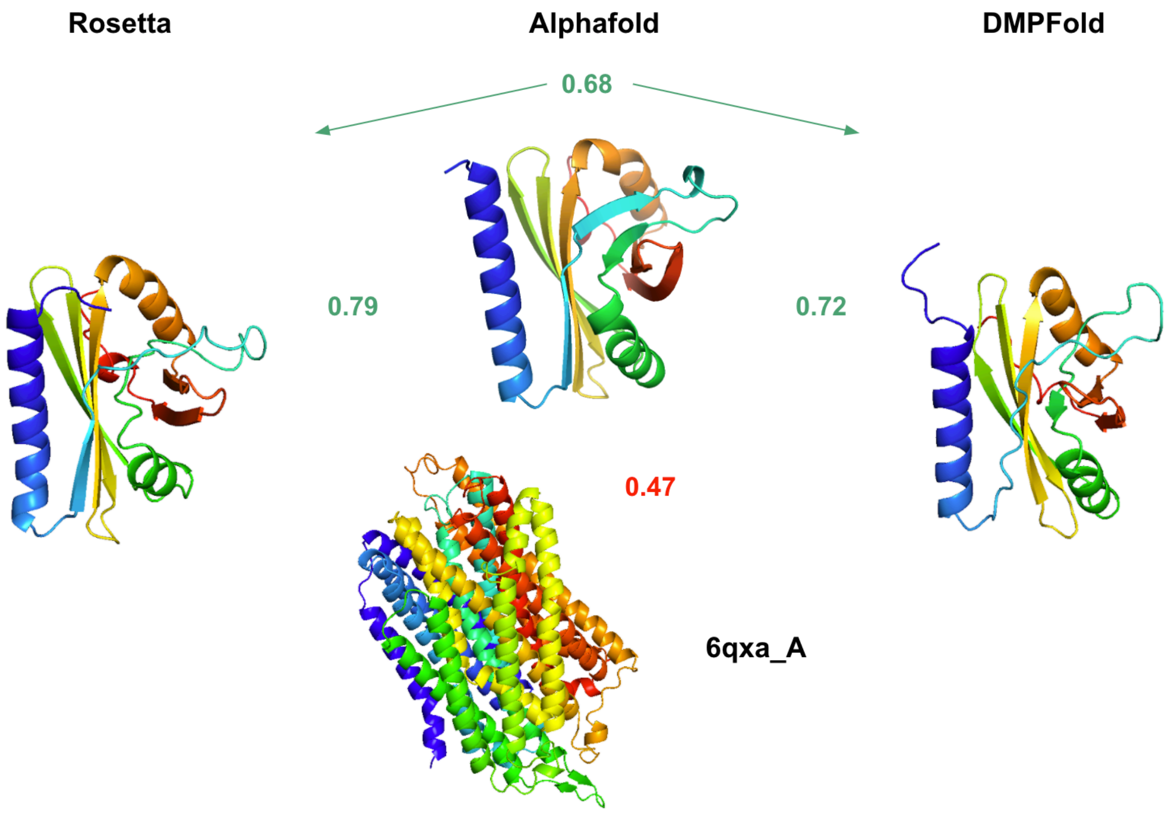

	Rosetta	Alphafold	DMPFold
**Max CATH TM-score:**	0.44	0.43	0.42
**Max PDB TM-score:**	0.46	0.47	0.47
**MQA score / Coil content:**	0.67 / 19%	0.93 / 14%	0.68 / 19%

Supplementary Figure 54: MIP_00189616 (cluster 102 singleton) structure comparison between Rosetta, AlphaFold2 and DMPFold (upper models) and the most similar PDB to the AlphaFold2 prediction (lower structure). Mutual TM-scores are shown in between. Additional parameters are listed in the table.

- singleton cluster with high agreement between predicted models and low agreement to PDB structure
- pTM (global score) for AlphaFold2: 0.88

Novel fold example #3

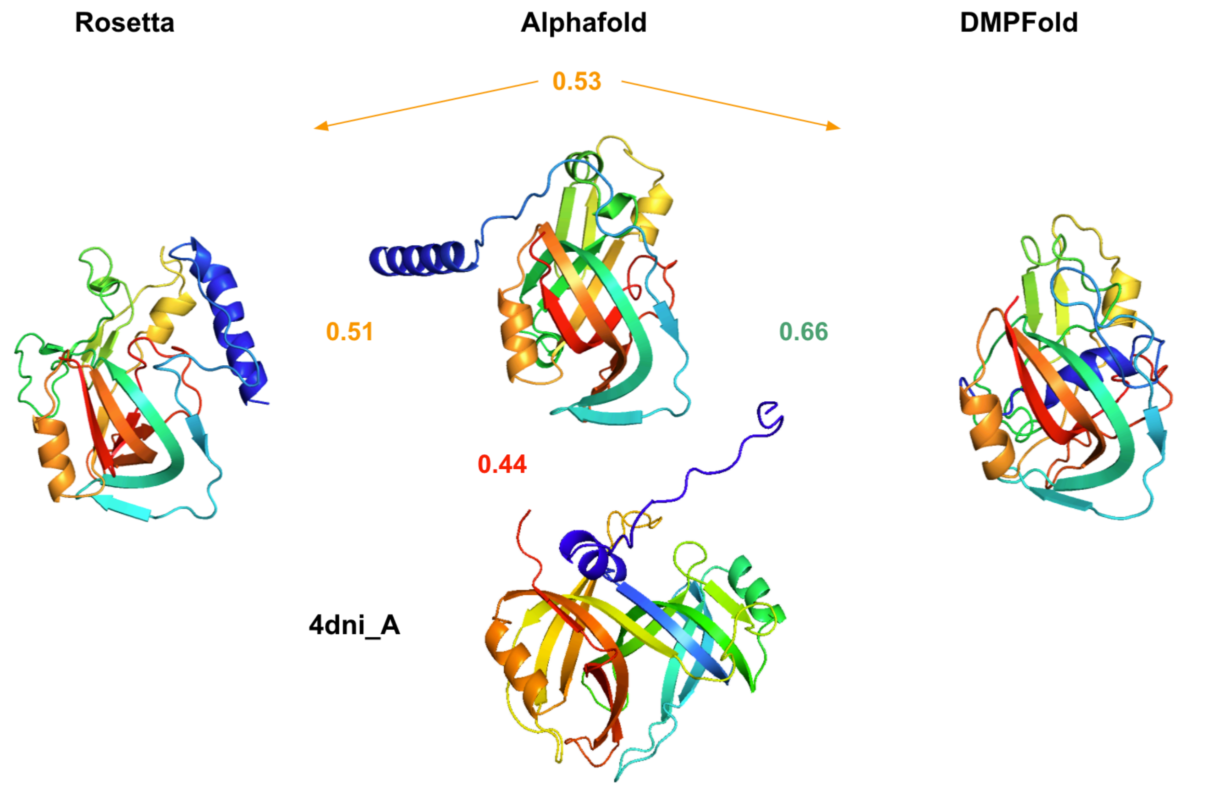

	Rosetta	Alphafold	DMPFold
**Max CATH TM-score:**	0.42	0.41	0.41
**Max PDB TM-score:**	0.46	0.44	0.41
**MQA score / Coil content:**	0.46 / 30%	0.93 / 18%	0.57 / 36%

Supplementary Figure 55: MIP_00307861 (cluster 149 representative) structure comparison between Rosetta, AlphaFold2 and DMPFold (upper models) and the most similar PDB to the AlphaFold2 prediction (lower structure). Mutual TM-scores are shown in between. Additional parameters are listed in the table.

- Lower agreement due to disordered (83% on average) N-terminus alpha helix which is predicted differently by all methods
- Mean pLDDT for AlphaFold2 for that fragment is equal 72% (confident)
- pTM (global score) for AlphaFold2: 0.85

Novel fold example #4

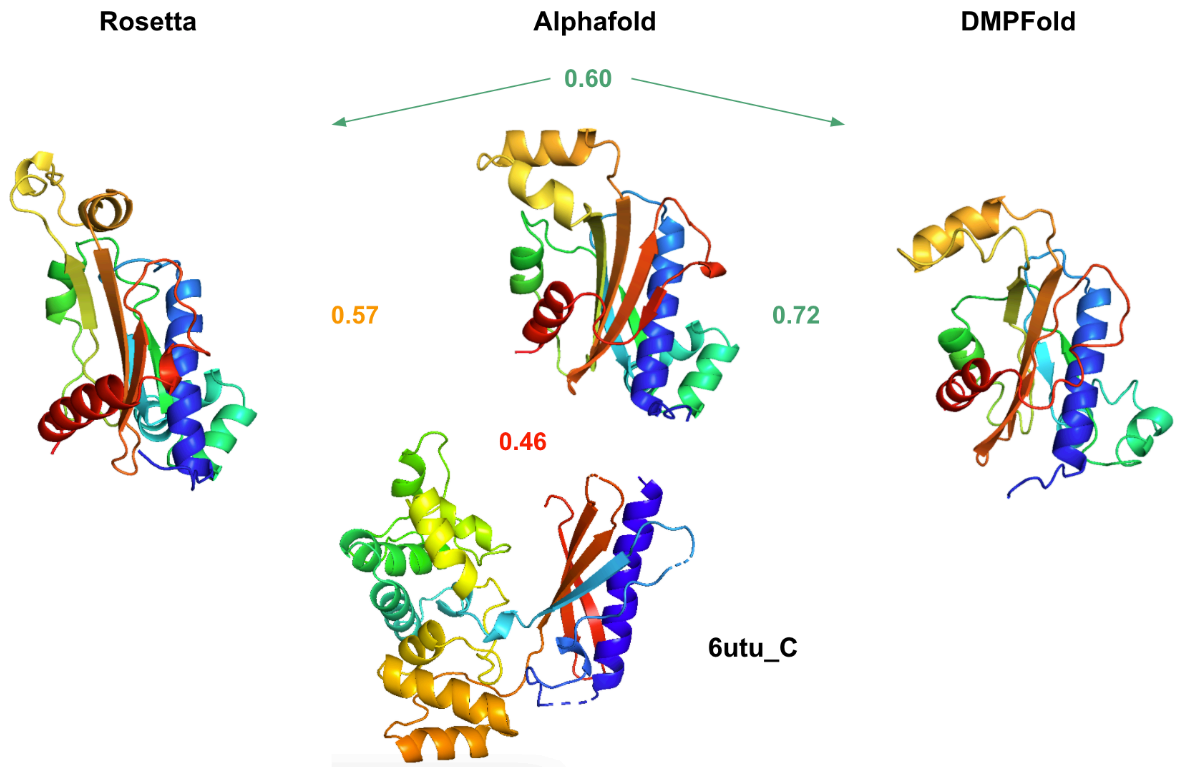

	Rosetta	Alphafold	DMPFold
**Max CATH TM-score:**	0.43	0.42	0.42
**Max PDB TM-score:**	0.46	0.46	0.47
**MQA score / Coil content:**	0.57 / 30%	0.93 / 22%	0.66 / 37%

Supplementary Figure 56: MIP_00261729 (cluster 158 representative) structure comparison between Rosetta, AlphaFold2 and DMPFold (upper models) and the most similar PDB to the AlphaFold2 prediction (lower structure). Mutual TM-scores are shown in between. Additional parameters are listed in the table.

- As in *Novel fold example* #2, the first 6 residues in N-terminus are disordered and therefore predicted differently by all methods but it doesn't influence the results
- pTM (global score) for AlphaFold2: 0.88

7. Largest novel fold clusters

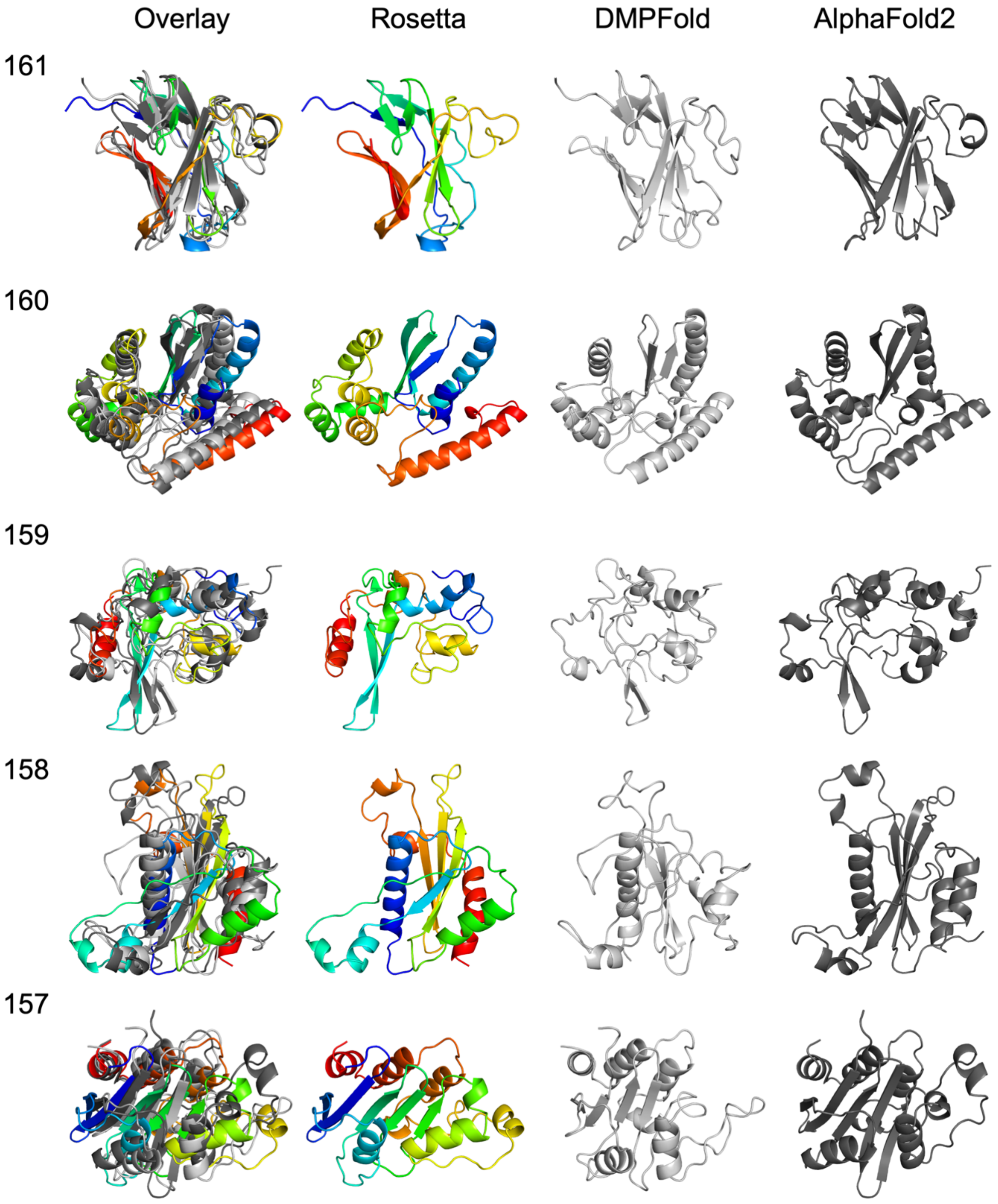

Supplementary Figure 57: Comparison of modeling methods for the largest novel fold clusters.

8. Functions of the novel fold proteins

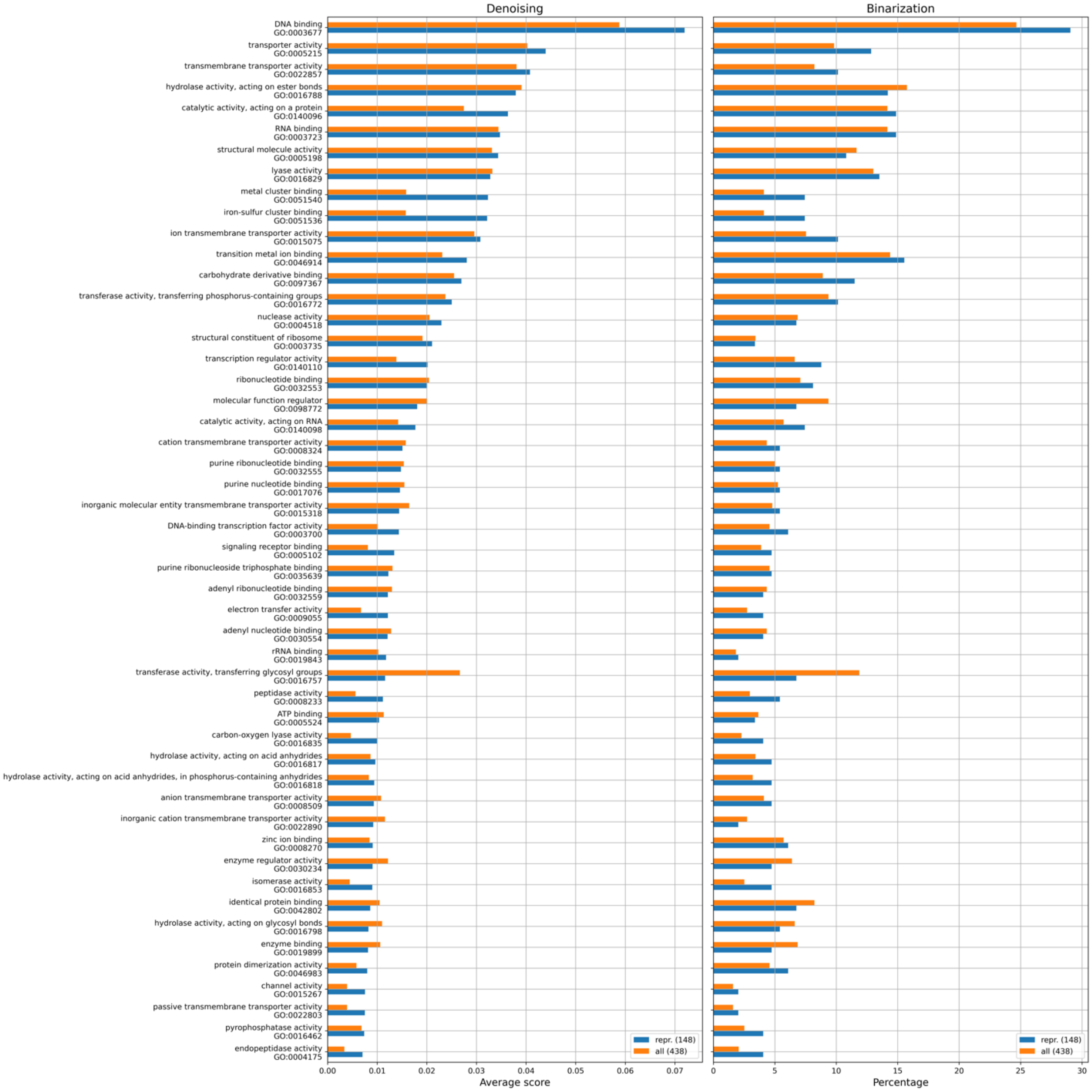

Supplementary Figure 58: The 50 most prevalent functions for the novel fold dataset, covering either the 148 representatives for all novel fold clusters (blue) or all 438 models (orange). For the histogram in the left panel, the function vectors were denoised with a score cutoff of 0.1, then summed over the models in the novel fold dataset and then sorted from the largest to the smallest scores. This means that the left histogram takes into account the magnitude of the scores. For the histogram on the right, the score vectors were binarized: scores ≤ 0.1 were set to 0 and scores > 0.1 were set to 1. Score vectors were summed over the models in the novel fold dataset and then sorted from the largest to the smallest scores. This means that the histogram on the right does not consider the magnitude of the scores but rather the prevalence of these particular functions.

Supplementary Note 6: Protein universe visualization

The details about how we embedded the models from the MIP visualization dataset into the 3D structure space are described in the Methods section of the main paper - an overview is shown in Supplementary Figure 59. In the following sections we show visualizations of the protein universe using both UMAP and Principal Component Analysis (PCA), color coded by various protein features. Each dot in the protein universe represents one model from the MIP visualization dataset.

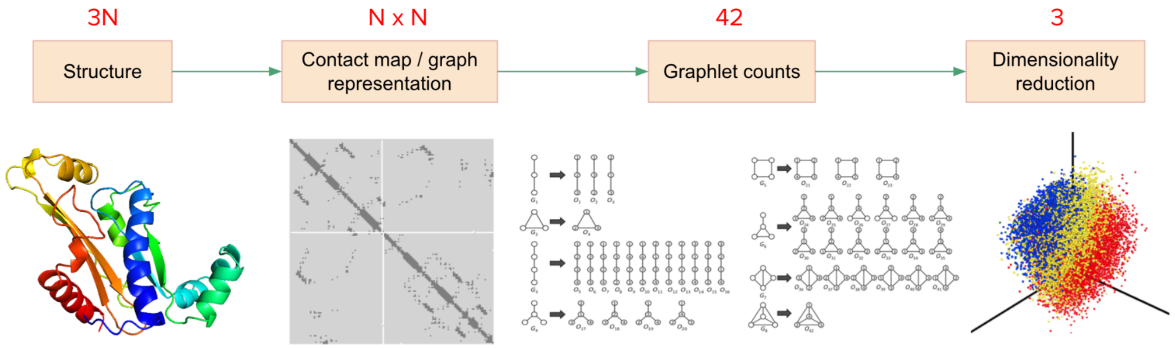

Supplementary Figure 59: An overview of the protein visualization pipeline. N denotes the number of residues in the structure. The graphlet count image was reprinted without changes and with permission from reference[31], which was published under a Creative Commons license (https://creativecommons.org/licenses/by/4.0/).

1. Protein features mapped onto the protein universe visualization

Sequence length

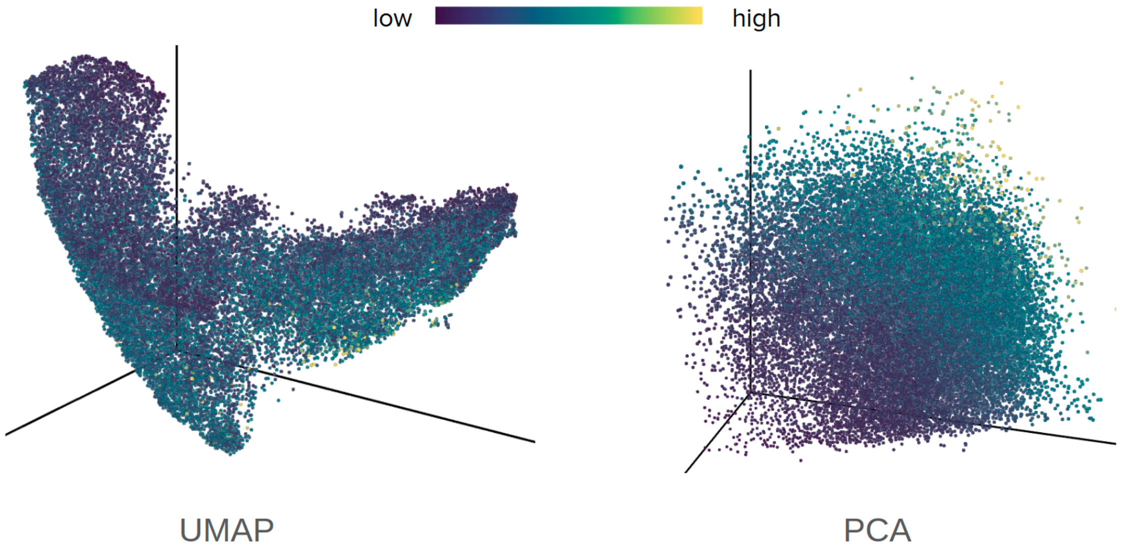

Supplementary Figure 60: 3D structure space visualization for the sequence length.

- Strong gradient in sequence length might be observed (especially for PCA).
- The largest proteins are placed in the bottom right in the UMAP plot and in the top right in the PCA plot (they correspond to CATH proteins with > 1000 residues).

CATH classes

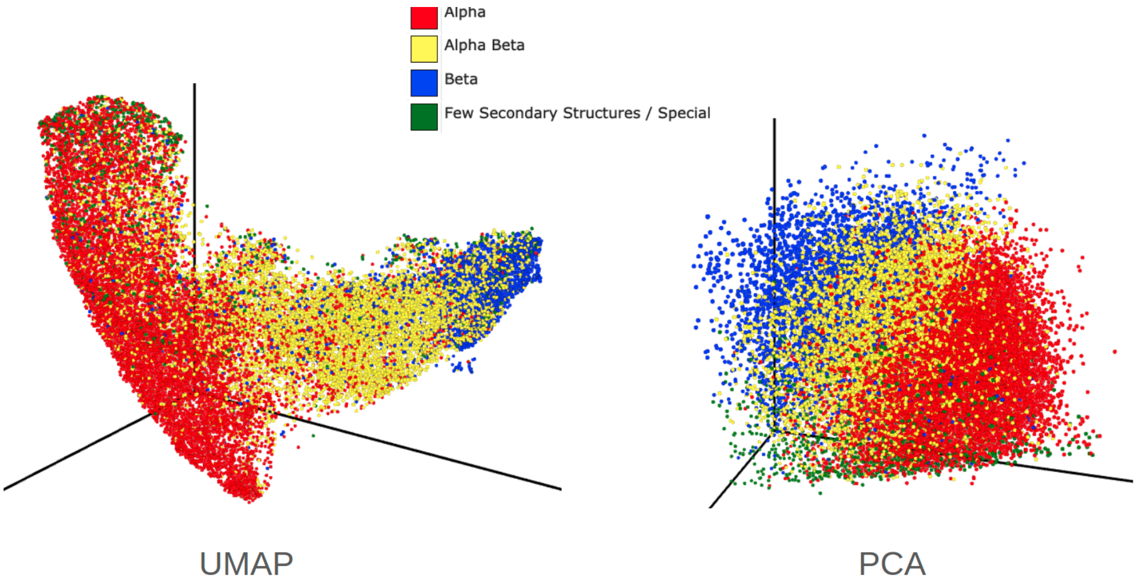

Supplementary Figure 61: 3D structure space visualization for the CATH classes.

- Structure space is continuous (general conclusion to all plots).
- Strong separation between CATH classes.
- Majority of green points (corresponding to 4th and 6th CATH classes) come from CATH structures - few Rosetta and DMPFold models were annotated this way.
- Majority of points on the outskirts in the PCA plot (in green) are also mainly from CATH.

Relative contact order

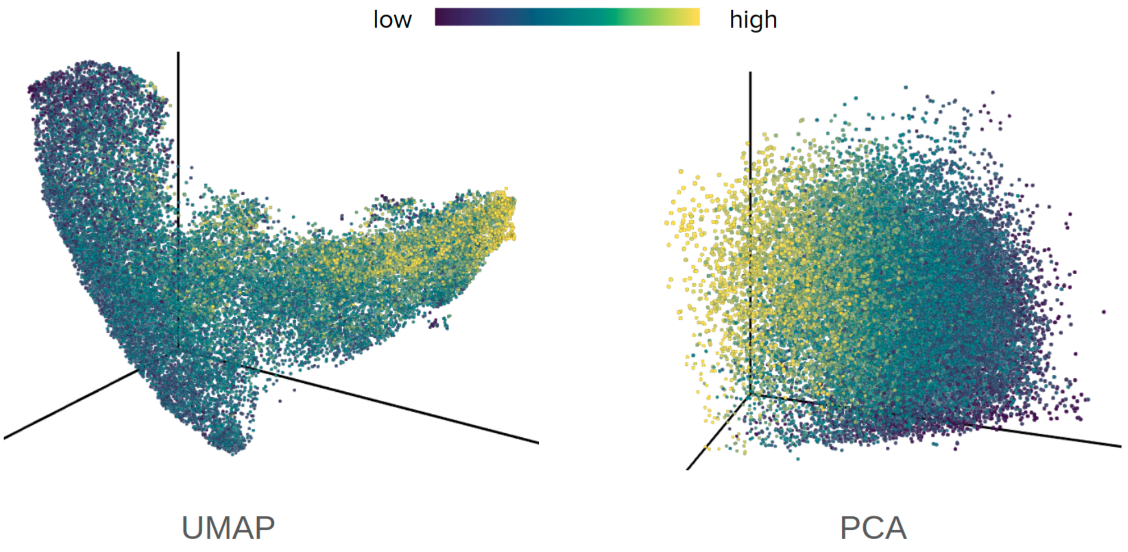

Supplementary Figure 62: 3D structure space visualization for relative contact order.

- Clear correlation between CATH class and relative contact order. Proteins with higher strand content generally have a higher relative contact order due to long-range contacts, which are fewer in alpha-helical proteins.

Alpha-helical transmembrane spans

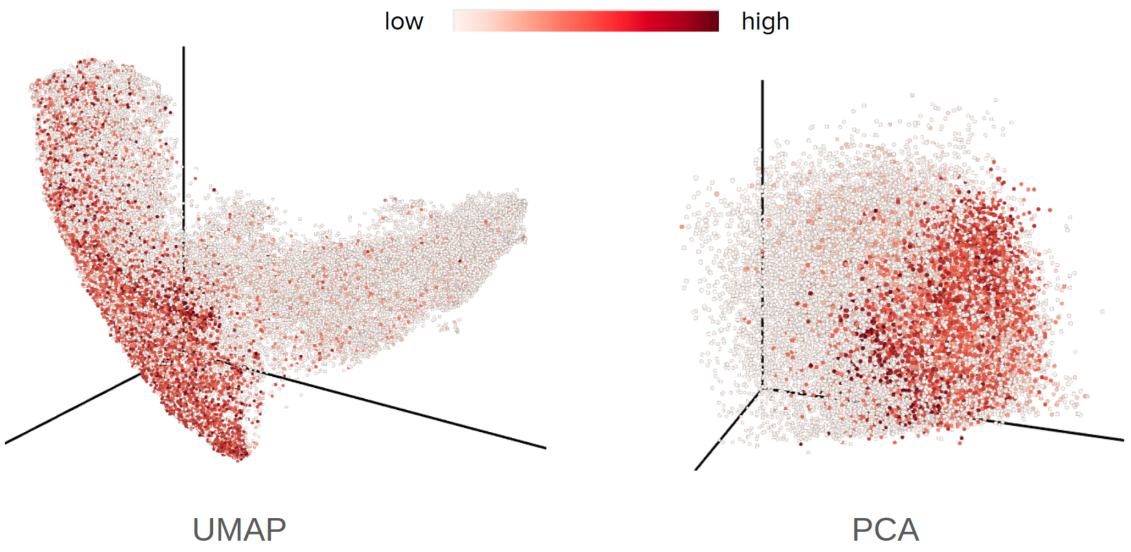

Supplementary Figure 63: 3D structure space visualization for alpha-helical transmembrane spans.

Beta-strand transmembrane spans

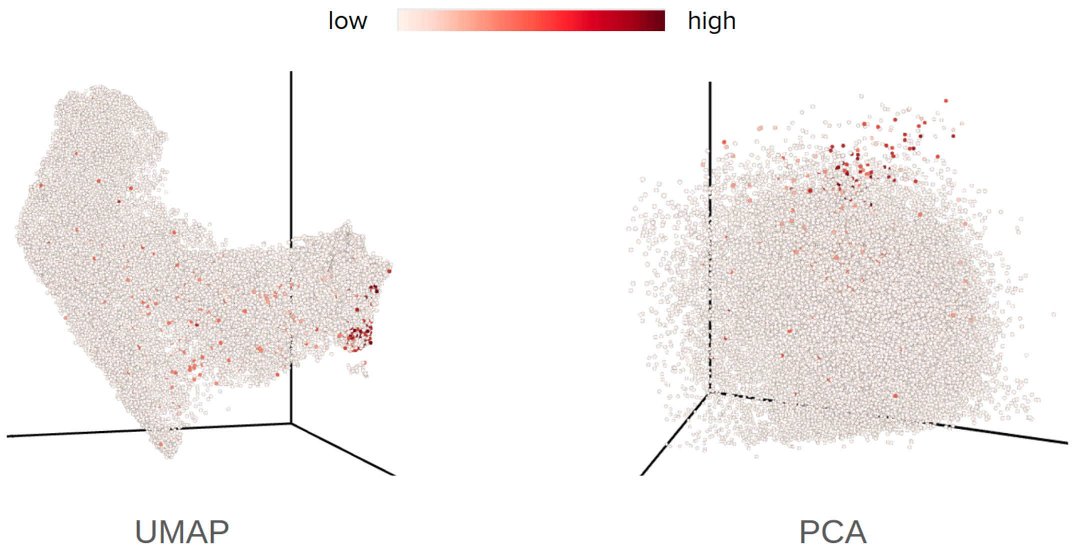

Supplementary Figure 64: 3D structure space visualization: beta-strand transmembrane spans.

- Alpha/beta transmembrane spans are grouped mainly in the alpha/beta region. Note that alpha/beta transmembrane spans were predicted using OCTOPUS/BOCTOPUS and were predicted using the protein sequence only.

Putative novel folds

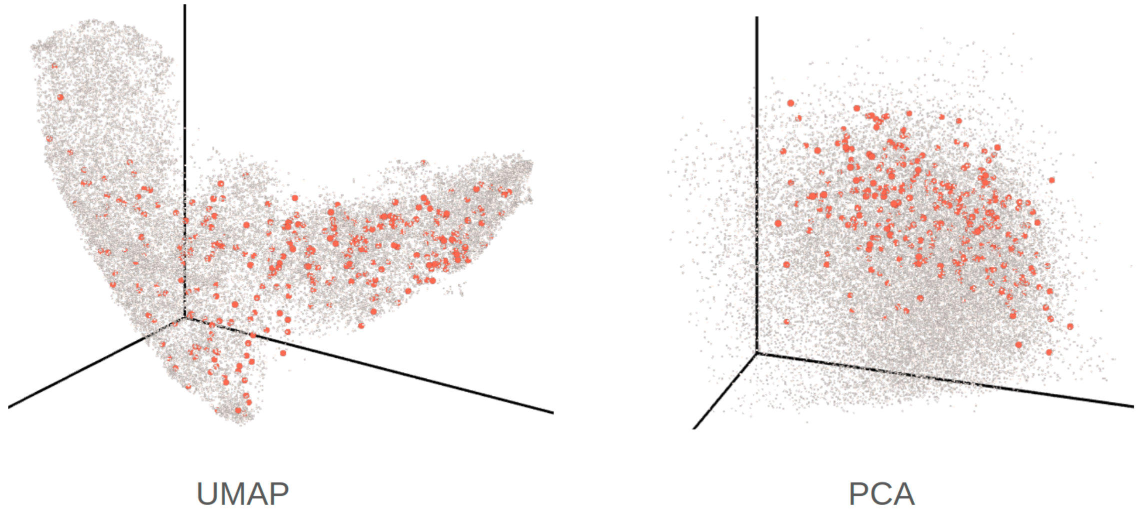

Supplementary Figure 65: 3D structure space visualization for 161 putative novel folds.

- Putative novel folds are mainly in alpha/beta proteins and cover the entire space with fewer novel folds in purely alpha or purely beta proteins.

2. Sample structures

In order to have a better intuition of what structures are located in specific parts of our UMAP and PCA projections, in Supplementary Figure 66 we identified positions of our novel fold examples and denoted a few representative structures on the edges (presented in Supplementary Figure 67 and Supplementary Figure 68).

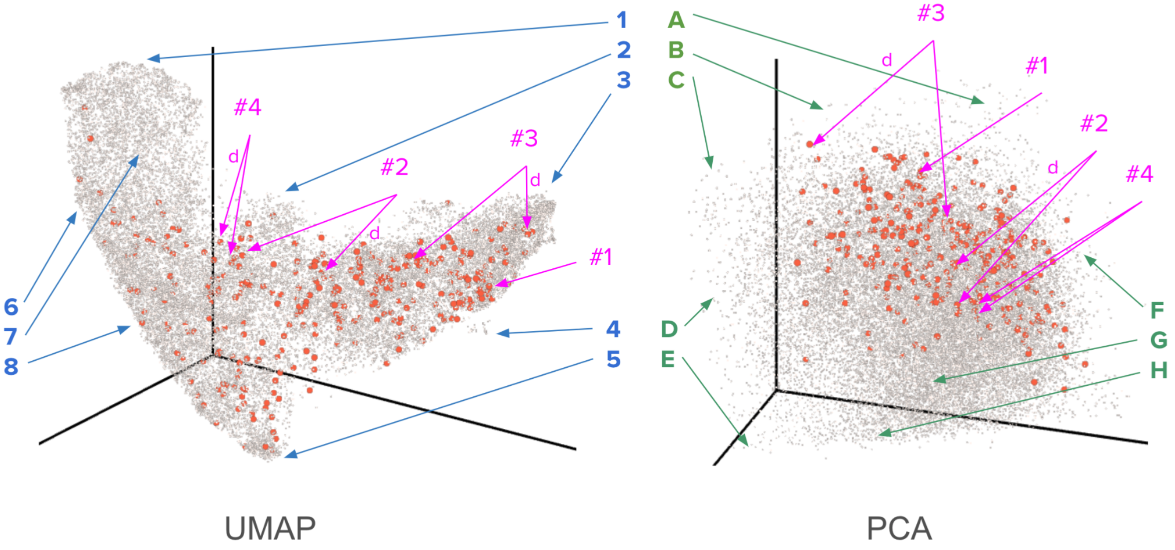

Supplementary Figure 66: 3D structure space visualization: sample structures (shown in Supplementary Figure 67 and Supplementary Figure 68) among putative novel folds. Blue and green arrows correspond to sample structures placed on the edges in UMAP and PCA projections respectively. Pink arrows point to putative novel fold examples in Supplementary Figure 53 – 56 (the arrow labelled with 'd' points to the DMPFold model, the other arrow points to the Rosetta model).

The Rosetta and DMPFold models are close in 3D space for the novel fold examples #1 and #4. Larger distances are observed for the novel fold examples #2 and #3 using both methods UMAP and PCA. For example #3 the larger distance is likely due to the N-terminal alpha helix position which significantly changes the graphlets counts. Importantly, examples shown in Supplementary Figure 67 and Supplementary Figure 68 agree with our expectations with respect to alpha/beta-like structure positions, sequence length and transmembrane protein locations.

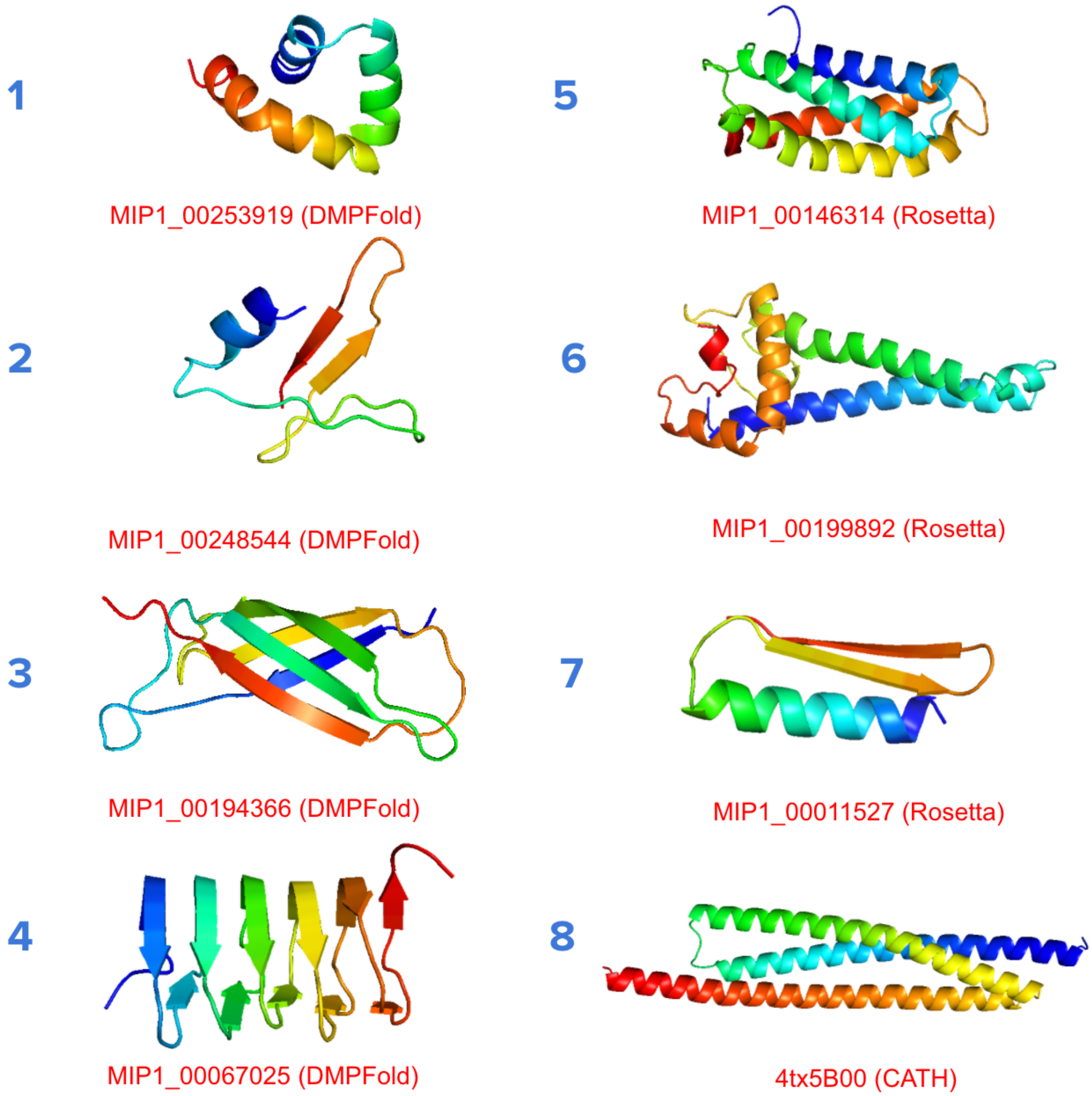

Supplementary Figure 67: Sample structures presented in Supplementary Figure 66 (UMAP projection).

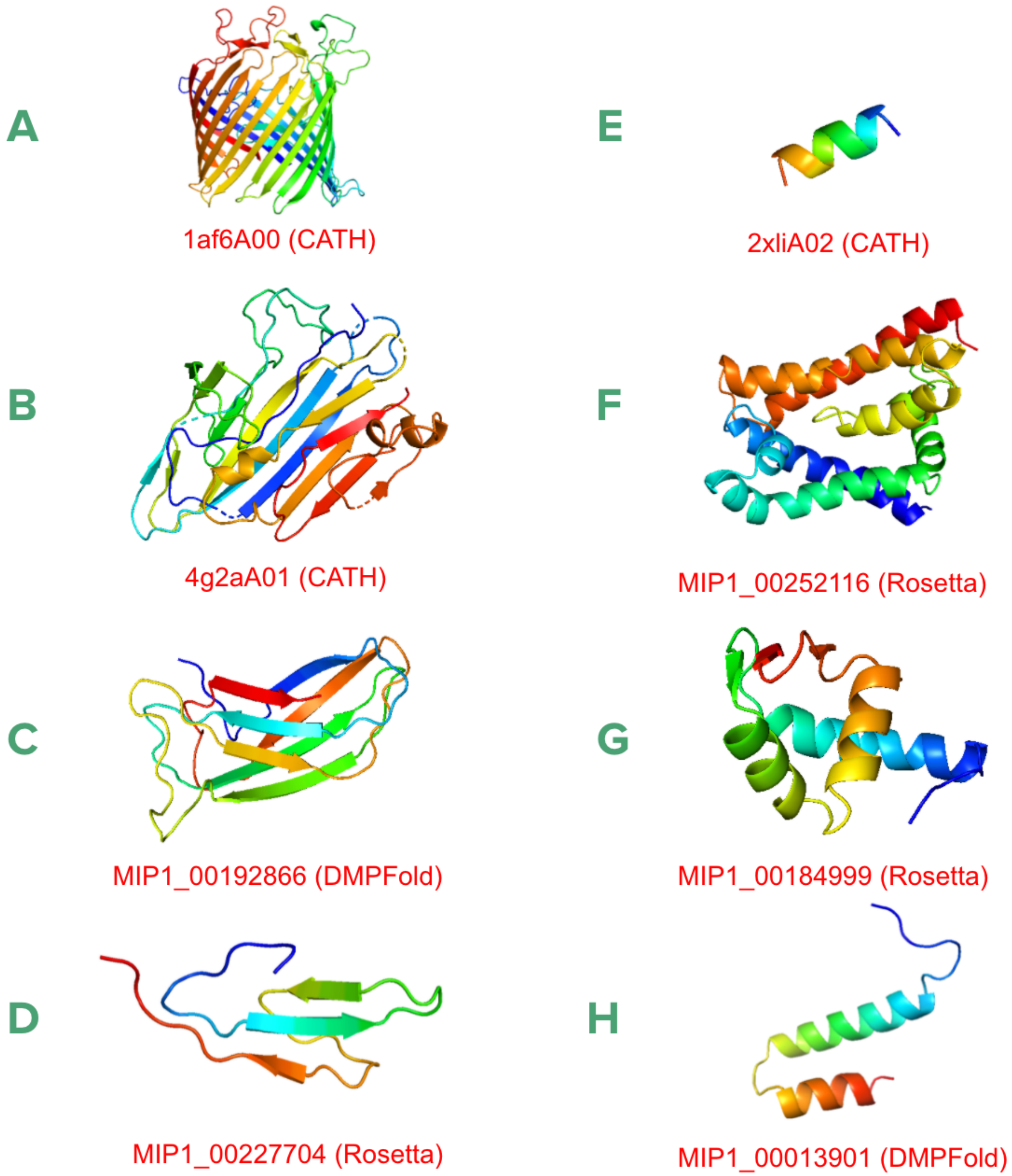

Supplementary Figure 68: Sample structure presented in Supplementary Figure 66 (PCA projection).

3. Caveat

Graphlet counts are simple and in some cases perform better than the other, more sophisticated methods[31] but they reflect local rather than global protein geometry. Therefore, when looking at specific examples in UMAP/PCA projections we can find different folds close to each other, which

creates potential room for improvements. Nevertheless, this approach is sufficient for our needs and in general agreement TM-score increases if Rosetta and DMPFold models are closer to each other (see Supplementary Figure 69). The overall topology created by UMAP visualization should be interpreted with caution, as its shape will depend on starting parameters and stochastic effects. Still, the overall features of this projection do not change with the choice of parameters.

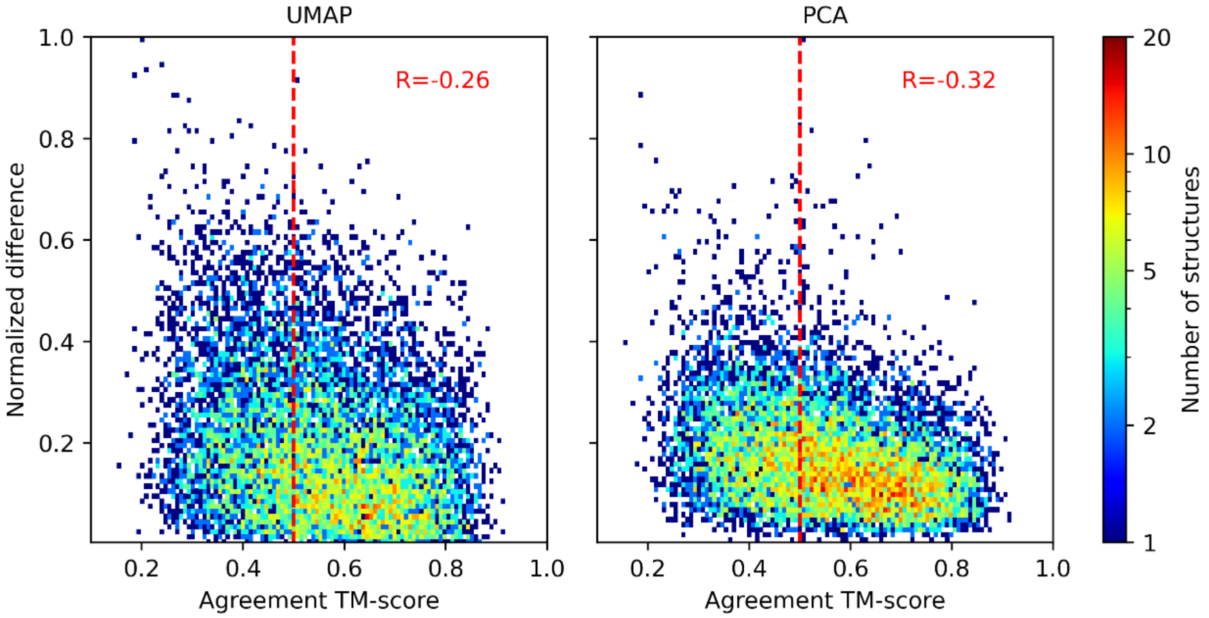

Supplementary Figure 69: Euclidean distance between Rosetta and DMPFold predictions for UMAP (left) and PCA (right) visualizations. Pearson R correlation coefficient is depicted in red in the upper right corners. For easy comparison between PCA and UMAP, the y-axes are normalised by the largest value.

Supplementary Note 7: Sanity checks for sequence-structure-function relationships

For initial sanity checks, we compared our *Random5000* data to subsets of the PDB. For this, we constructed two additional datasets by clustering the PDB based on sequence identity:

1. 1000 chains with pairwise sequence identities distributed between 0 and 100% (with 10% step size - 10perc BINS)
2. sequence identity threshold ≤ 20% (20perc THR)

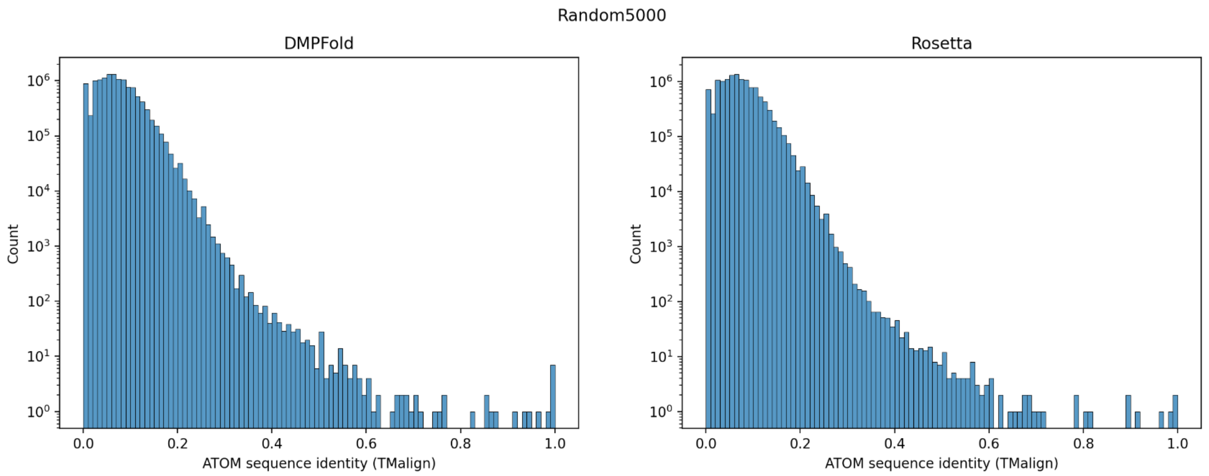

Supplementary Figure 70: By design, our MIP datasets (here from Random5000) exhibit very low pairwise sequence identity. The distribution has a maximum around 10% due to the pairwise nature of the comparison, even though some sequences have substantially higher sequence identity due to domain splitting, which allows few domains to be more similar.

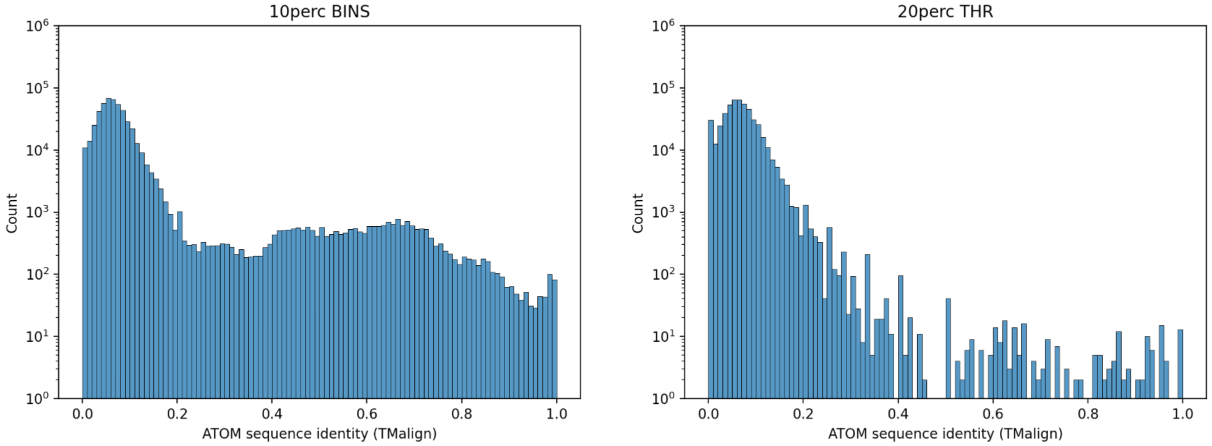

Supplementary Figure 71: Pairwise sequence identities on the subsets of the PDB (Left) that covers the range of sequence identities (10perc bins) and (Right) with a sequence identity cutoff of 20% (20perc thr). The binned subset on the left has higher baseline counts because the pairwise sequence identities cover the whole range.

1. Sequence-structure comparison

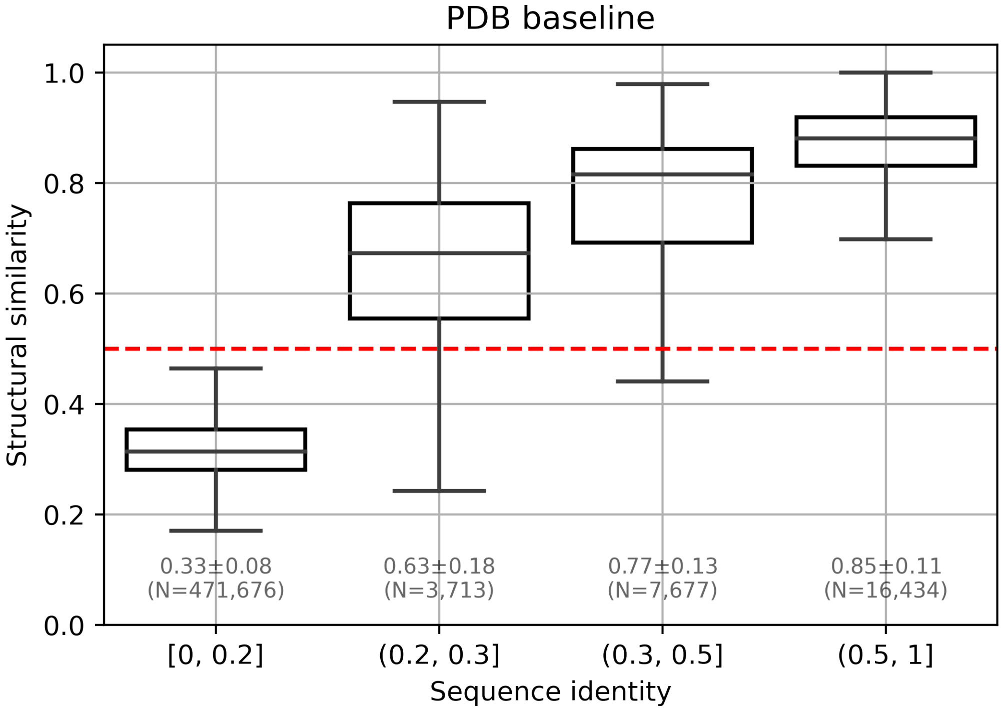

*Supplementary Figure 72: Trend between sequence identity and structural similarity (using the TMscore) for the PDB baseline in Fig. 2a. The box extends from Q1 to Q3 quartile values of the data, with a line at the median (Q2). The whiskers extend from the edges of box to show the range of the data. By Pandas default, they extend no more than 1.5 * inter-quartile range (IQR = Q3 - Q1) from the edges of the box, ending at the farthest data point within that interval. Below each box is the mean +/- stdev and the number of data points.*

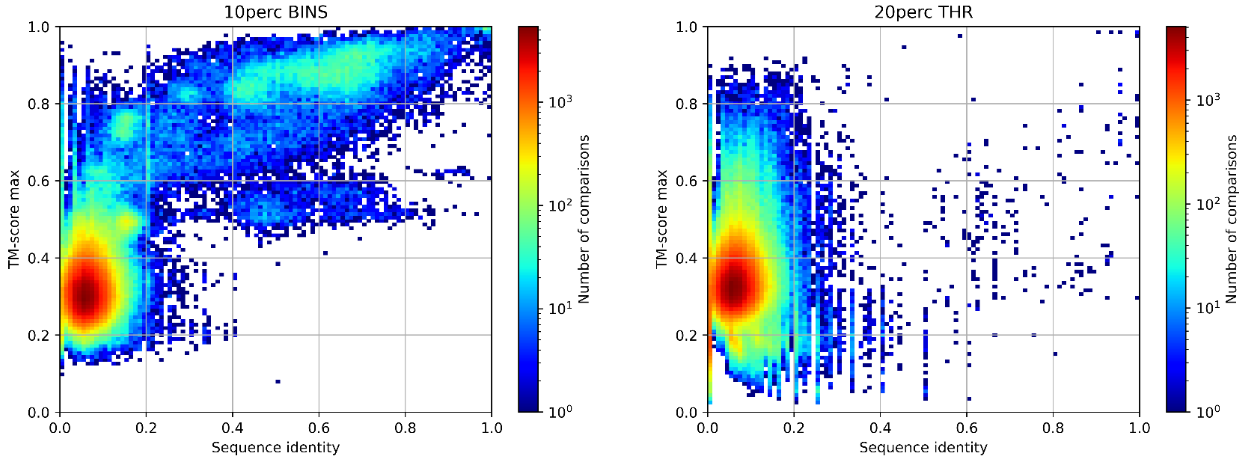

Supplementary Figure 73: Pairwise TM-score plotted over the sequence identity of the two PDB subsets described at the beginning of this section. These plots imply that sequence identity ≤ 0.2 implies low structural similarity and sequence identity > 0.2 implies high structural similarity. The binned subset of the PDB on the left is a more accurate representation of the entire PDB, which covers all pairwise sequence identities. The subset with the threshold is a more accurate representation of our MIP dataset because we picked the sequences specifically to have a low pairwise sequence identity. This is confirmed with comparing the right panel with panel (b) in manuscript Fig. 2.

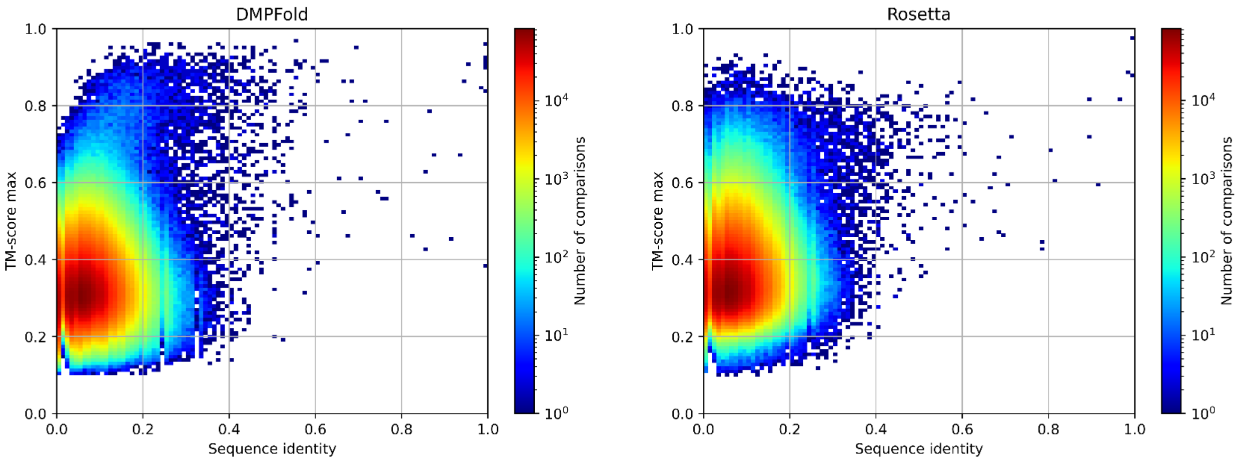

Supplementary Figure 74: Pairwise TM-score plotted over the sequence identity for the DMPFold (left) and Rosetta (right) models in the Random5000 dataset.

2. Sequence-function comparison

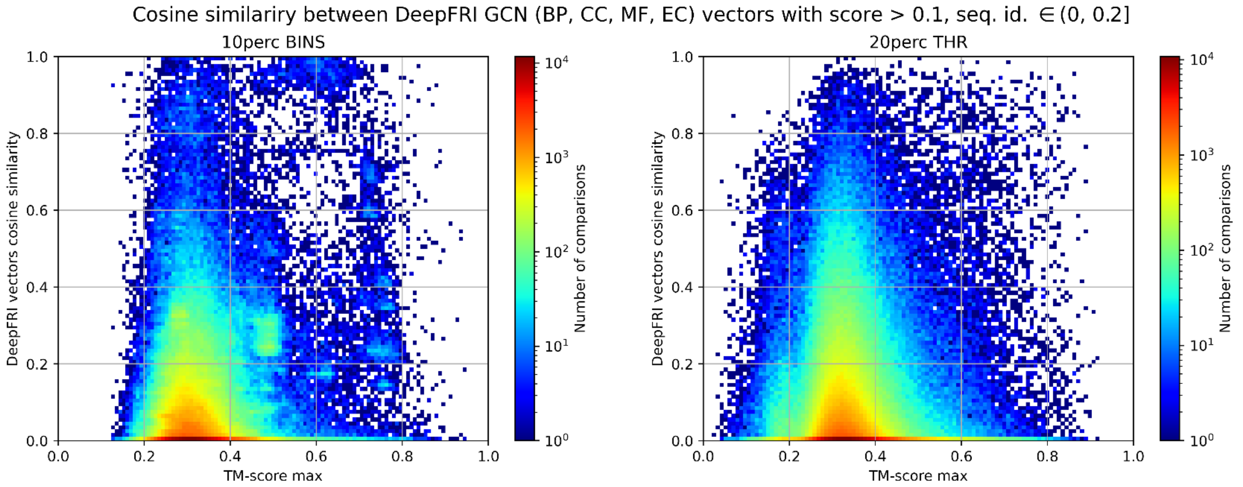

Supplementary Figure 75: Functional similarity (cosine similarity of DeepFRI vectors) plotted over the structural similarity (TM-score) for dissimilar sequences (sequence identities between 0 and 20%) for the binned PDB subset (left) and the PDB subset with a sequence identity cutoff of 20%. These plots show low structural similarity and therefore low functional similarity for the majority of the datasets.

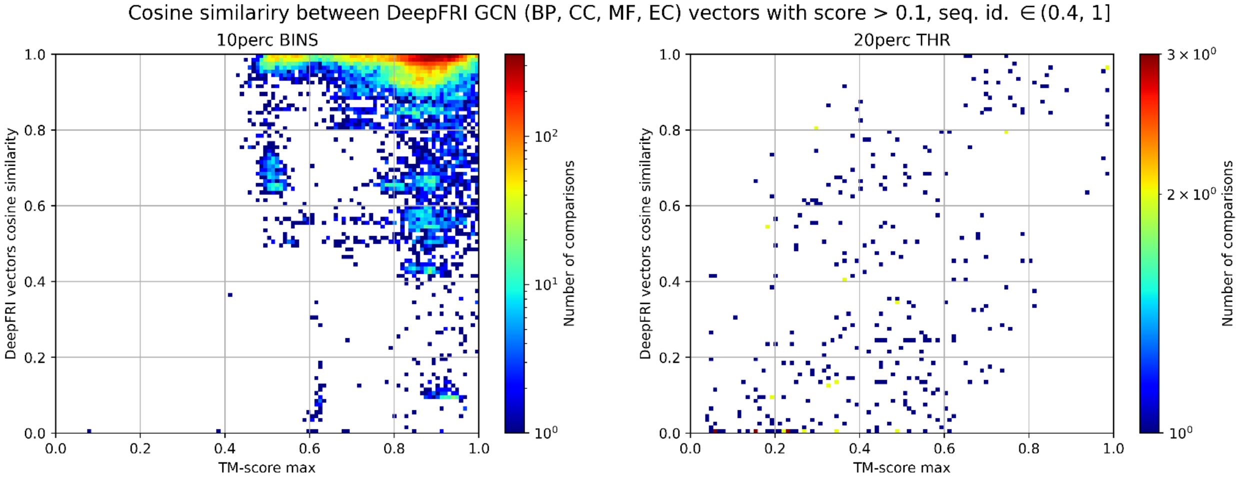

Supplementary Figure 76: Functional similarity (cosine similarity of DeepFRI vectors) plotted over the structural similarity (TM-score) for similar sequences (sequence identities between 40 and 100%) for the binned PDB subset (left) and the PDB subset with a sequence identity cutoff of 20%. These plots show low structural similarity and therefore low functional similarity for the majority of the datasets.

3. Structure-function comparison

We computed structural similarities within clusters of models that have the same functional prediction, i.e. the same GO term. To achieve this, we first grouped MIP models with DeepFRI predictions (score >= 0.2) were by GO-term. Only GO-terms that have 50 - 1000 models within *MIP_curated* were selected. Pairwise structural similarity was computed separately for each GO-term as maximum TM-score of two superimposed MIP structures. We find that structural similarity increases with increasing information content in each GO-group (BP, MF, CC) both for Rosetta and DMPFold predictions (Fig. S77). This is expected because higher information content corresponds to more specific functions which can be represented by a more limited number of folds - therefore their structural similarity is higher.

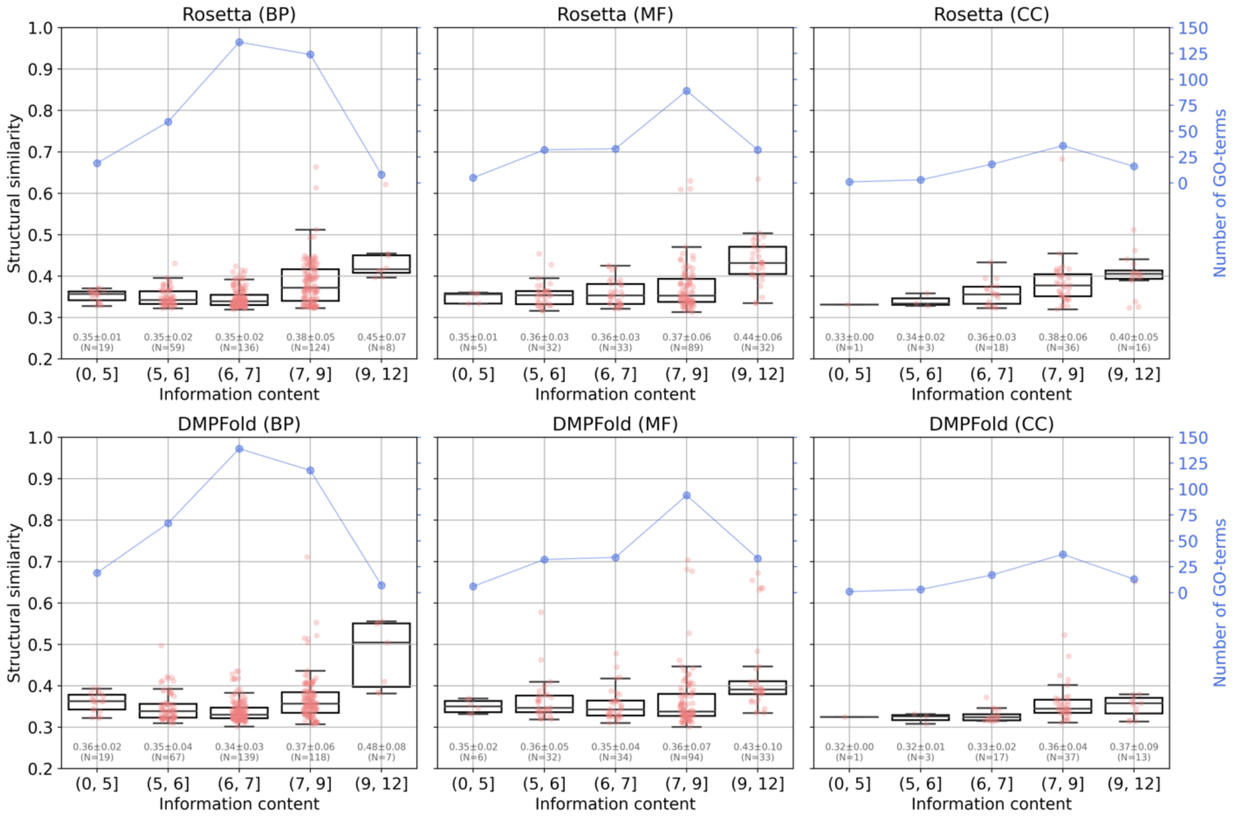

*Supplementary Figure 77: Box-and-whisker plots showing structural similarity across GO groups for MIP_curated Rosetta (upper panels) and DMPFold (lower panels) models sorted by increasing information content. Each point on the plot corresponds to the mean of such TM-scores (one point for each GO-term). The blue curve depicts the number of compared GO-terms. The box extends from Q1 to Q3 quartile values of the data, with a line at the median (Q2). The whiskers extend from the edges of box to show the range of the data. By Pandas default, they extend no more than 1.5 * inter-quartile range (IQR = Q3 - Q1) from the edges of the box, ending at the farthest data point within that interval. Outliers are plotted as separate dots. Below each box is the mean +/- stdev and the number of data points.*

Supplementary Note 8: Structure Function Motifs

1. Functional diversity of proteins with the same structure

Fig. 3 shows examples where we look at the functional diversity of proteins with the same structure. For the proteins in Fig. 3a the TM-scores are 0.55 and 0.70 for Rosetta / DMPFold models, respectively. For the proteins in Fig. 3b the TM-scores are 0.58 / 0.82 for Rosetta / DMPFold models, respectively. High similarities of the contact maps between the two proteins in each example indicate that it is the embedding of the structural motif (white rectangle) in the tertiary structure that is responsible for the function, instead of just the secondary structure. We also show the class activation maps with the functional predictions mapped onto the sequence to highlight the similarities there.

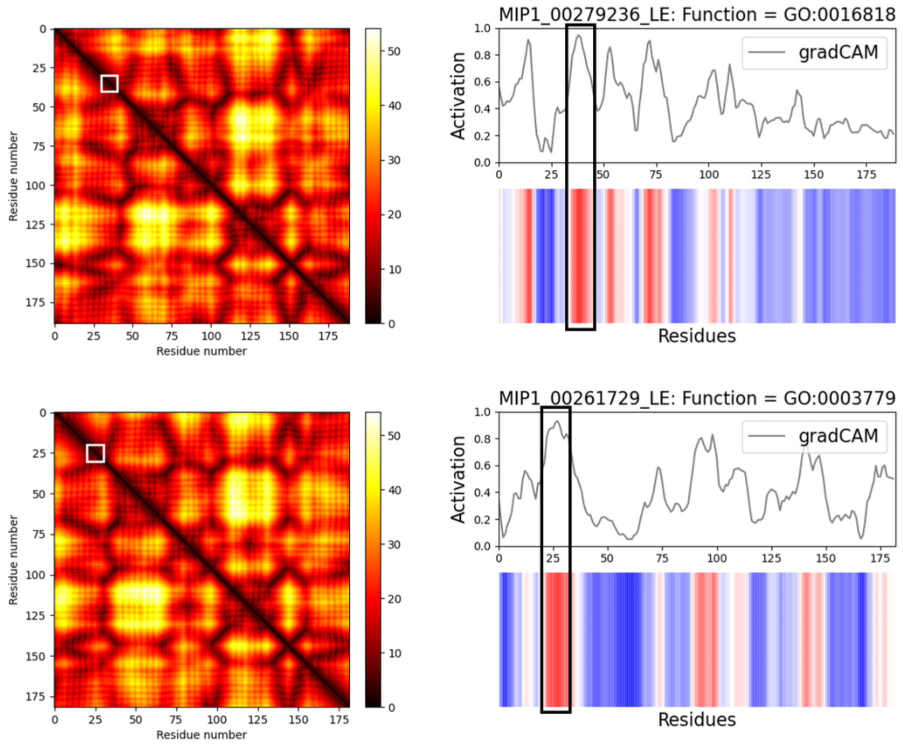

Supplementary Figure 78: Contact maps (left) and class activation maps (right) for both proteins in Fig. 3a. The boxes in white and black highlight the functional motif with the highest function prediction score.

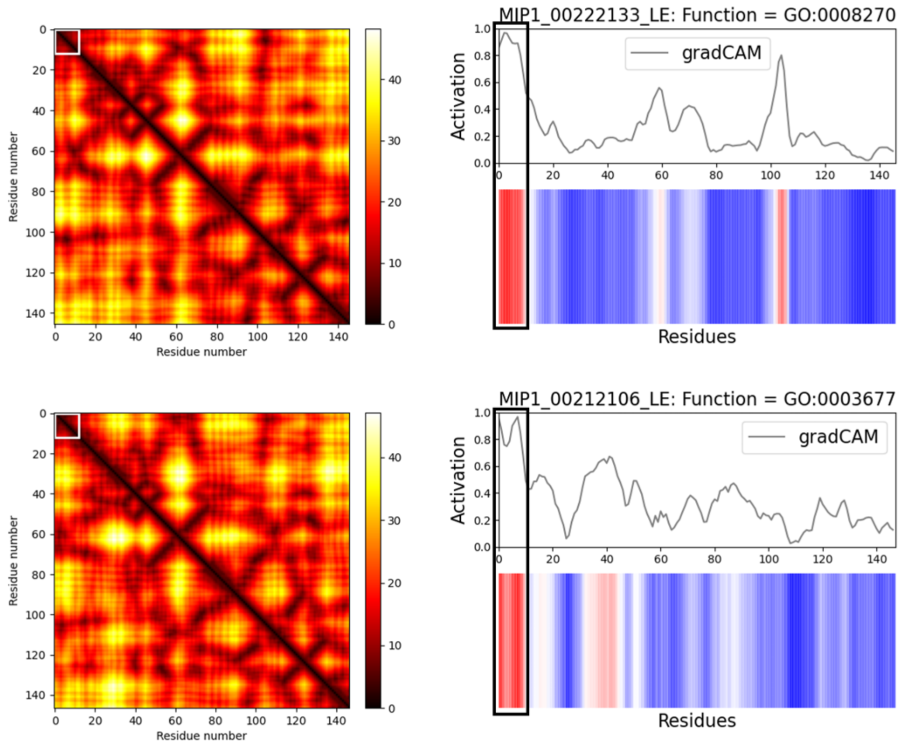

Supplementary Figure 79: Contact maps (left) and class activation maps (right) for both proteins in Fig. 3b. The boxes in white and black highlight the functional motif with the highest function prediction score.

2. Structural diversity of proteins with the same function

In Fig. 4 we highlight several cases where we explored the structural diversity of proteins with the same predicted function. Here we take a closer look at some of the highlighted structures and their functional relationships. For each of the three functions (in panels a, b, and c), Fig. 4 shows structural relationships of the proteins in each functional cluster via a heatmap (through pairwise TM-scores). Specific models are then shown on the right.

3. Carbohydrate binding (MF GO:0030246) - Fig. 4a

This functional cluster for carbohydrate binding covers many folds but includes one major fold (cluster shown in (a) as indicated by the yellow square in top-left corner of the heatmap in Fig. 4a. Additionally, this cluster represents the largest cluster of proposed novels folds from this study (example #1 in the *New Fold examples* section above). This molecular function is two levels below the top of the GO hierarchy and has more than 40 child terms. There are ~5,100 entries in the PDB annotated with this label. Carbohydrate binding is an important protein function for all microbes, particularly in gut microbiomes where carbohydrate metabolism benefits both the microbe and its host, as recently reviewed[32].

Carbohydrate binding modules (CBMs) direct carbohydrate-active enzymes (CAZymes) to specific carbohydrate motifs. CAZymes have been cataloged and classified in the CAZy Database[33]. CBMs have been classified based on their type and fold family[34]; the CAZy database currently describes 89 CBM families, which we compared our MIP models to (Supplementary Figure 80).

The novel fold cluster has features frequently found in carbohydrate binding modules such as a beta-sandwich topology and clusters of large-hydrophobic residues being indicated as salient for carbohydrate binding functionality (Supplementary Figure 81A), but doesn’t have high structural similarity to any experimentally determined structures of CBM listed in the CAZy Database (Supplementary Figure 80) or any structures annotated as having carbohydrate binding functionality (GO:0030246). It could potentially be a new class of CBM but further analysis and experiments would be required to verify this possibility.

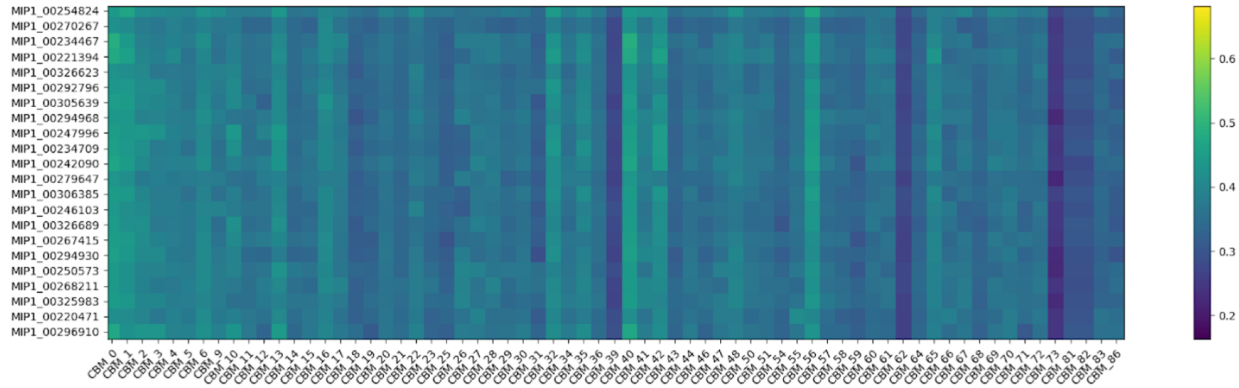

Supplementary Figure 80: Comparing MIP models with known Carbohydrate Binding Modules. Heatmap showing the highest TM-score between Rosetta models of MIP sequences belonging to the novel fold cluster predicted to have

carbohydrate binding functionality and a representative of experimentally determined structures of all 89 classes of carbohydrate binding modules listed in the CAZy database.

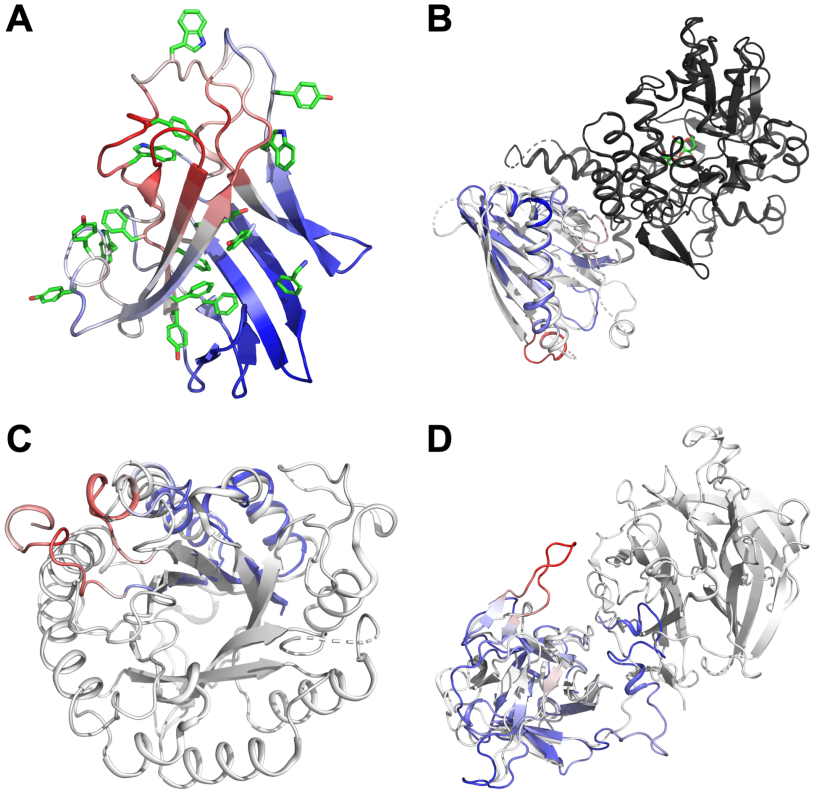

Supplementary Figure 81: (A) Representative structure of the novel fold cluster with PHE, TRP, TYR residues shown as green sticks. As in other Figures, MIP models are colored by DeepFRI score to indicate predicted saliency for the given function. (B - D) Rosetta predicted models of several MIP sequences super imposed on structurally similar experimentally determined structures. Experimentally determined structures used in superpositions are shown white or black. (B) MIP_00224264 superimposed on PDB ID 4XZV chain A (TM-score 0.63) of the SLMO1-TRIAP1 complex[35]. SLOM1 does not bind carbohydrates, but is associated with maltose-binding periplasmic protein TRIAP1. (C) MIP_00094272 superimposed on PDB ID 1LF1 chain A[36] (TM-score 0.64), the catalytic core of a glucoside hydrolyase. (D) MIP_00326772 superimposed on PDB ID 3VT1 chain D[37] (TM-score 0.63). The MIP sequence superimposes with the carbohydrate binding module (type 13) of a glycoside hydrolase.

4. Maintenance of CRISPR repeat elements (BP GO:0043571) - Fig. 4b

This functional cluster covers several folds but includes one major fold (cluster shown in (A) as indicated by the yellow square in the heatmap in Fig. 4b). This biological process has no child terms in the GO hierarchy and there are ~200 PDB entries with this label. The CRISPR sequences are DNA sequences found in the genomes of prokaryotic organisms like bacteria and archaea and they derive from DNA fragments of bacteriophages that previously infected those organisms. Hence, the CRISPR-Cas system functions as a microbial immune system and it is not surprising to find CRISPR sequences in our MIP database. There are few gene products associated with this function and they are associated with Cas1,2,5,6,9,D,and A. Cas1 and Cas2 identify the site in the bacterial genome where they insert viral DNA for later identification and ultimately cleavage by Cas9. As an example, the structure with PDB ID 5XVP[38] has four copies of Cas1 (chains

ABCD) and two copies of Cas2 (chains EF); Cas1 and Cas2 have a different fold. The structural cluster in (A) overlays with a large part of Cas2 and the salient residues highlighted by the function prediction DeepFRI bind DNA in the structure. Clusters marked (D) and (E) in Fig. 4b are similar to parts of Cas1 but don't overlay perfectly. Supplementary Figure 82 below shows the Rosetta predicted models of MIP_00052663 superimposed on 5XVP chain F and MIP_00011079 superimposed on 5XVP chain C.

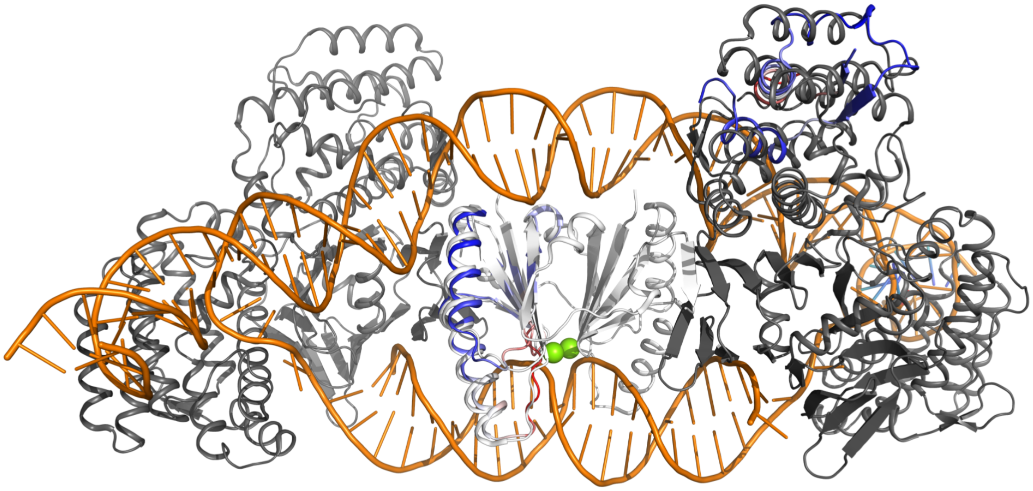

Fig. S82: MIP_00052663 aligned to 5XVP chain F (TM-score 0.87) and MIP_00011079 aligned to 5XVP chain C (TM-score 0.47).

5. Sole sub-class for lyases that do not belong in the other subclasses (EC 4.99.1.-) - Fig. 4c

As shown in Fig. 4c, all models in this functional cluster have the same fold and the salient residues overlap nicely across all structures. Lyases are enzymes that catalyze the breaking of chemical bonds by means other than hydrolysis or oxidation. Because they break bonds, lyases only require a single substrate, unlike for the reverse reaction, which requires two substrates. The E.C.4. class of enzymes are all lyases that cleave carbon-carbon bonds (E.C.4.1.), carbon-oxygen bonds (E.C.4.2.), carbon-nitrogen bonds (E.C.4.3.), carbon-sulfur bonds (E.C.4.4.), carbon-halide bonds (E.C.4.5.), and phosphorus-oxygen bonds (E.C.4.6.). The E.C.4.99.1.- class of lyases is therefore the "sole subclass for lyases that do not belong in the other subclasses" and covers several chelatases for iron, nickel, cobalt, and magnesium, dehydratases, a lyase and a ligase. None of the lyases in the other classes with known structures has the same fold as our predicted MIP models, even though there are structural similarities. Our models have an (αβ)x3 fold with sequential strand connections the other lyases have various (αβ)xN folds but their strand connections are non-sequential, with some strands inserting between existing ones. Supplementary Figure 83 below shows the Rosetta prediction of MIP_00077627 superimposed with PDB ID 5Z7T chain B[39]. The experimentally determined structure is of SirB, a ferrochelatase from *Bacillus subtilis*. The salient esidues for the function predicted by DeepFRI superimpose to residues in the experimentally determined structure that coordinate a Cobalt ion with which it was co-crystalized.

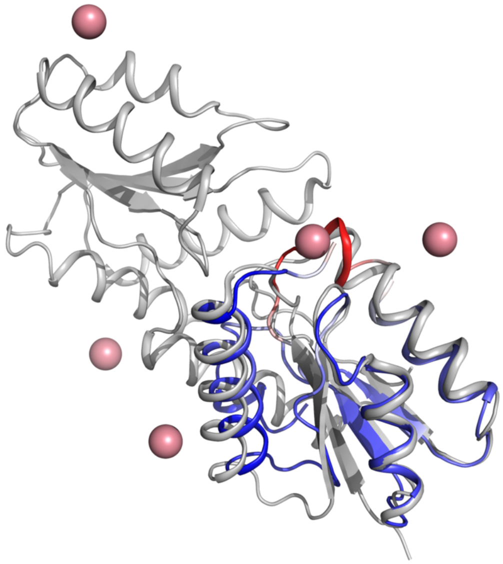

Supplementary Figure 83: MIP_00077627 aligned to 5Z7T chain B (TM-score 0.77).

Supplementary References

1. Mukherjee, S. *et al.* 1,003 reference genomes of bacterial and archaeal isolates expand coverage of the tree of life. *Nat. Biotechnol. 2017 357* **35**, 676–683 (2017).
2. Hyatt, D. *et al.* Prodigal: Prokaryotic gene recognition and translation initiation site identification. *BMC Bioinformatics* **11**, 1–11 (2010).
3. Mirdita, M. *et al.* Uniclust databases of clustered and deeply annotated protein sequences and alignments. *Nucleic Acids Res.* **45**, D170–D176 (2017).
4. Steinegger, M. & Söding, J. Clustering huge protein sequence sets in linear time. *Nat. Commun. 2018 91* **9**, 1–8 (2018).
5. Steinegger, M. & Söding, J. MMseqs2 enables sensitive protein sequence searching for the analysis of massive data sets. *Nat. Biotechnol. 2017 3511* **35**, 1026–1028 (2017).
6. Remmert, M., Biegert, A., Hauser, A. & Söding, J. HHblits: lightning-fast iterative protein sequence searching by HMM-HMM alignment. *Nat. Methods* **9**, 173–175 (2011).
7. Gront, D., Kulp, D. W., Vernon, R. M., Strauss, C. E. M. & Baker, D. Generalized Fragment Picking in Rosetta: Design, Protocols and Applications. *PLoS One* **6**, e23294 (2011).
8. Altschul, S. F. *et al.* Gapped BLAST and PSI-BLAST:a new generation of protein database search programs. *Nucleic Acids Res* **25**, 3389–3402 (1997).
9. Yang, Y., Faraggi, E., Zhao, H. & Zhou, Y. Improving protein fold recognition and template-based modeling by employing probabilistic-based matching between predicted one-dimensional structural properties of query and corresponding native properties of templates. *Bioinformatics* **27**, 2076–2082 (2011).
10. Jones, D. T. Protein secondary structure prediction based on position-specific scoring matrices. *J. Mol. Biol.* **292**, 195–202 (1999).
11. Balakrishnan, S., Kamisetty, H., Carbonell, J. G., Lee, S. I. & Langmead, C. J. Learning generative models for protein fold families. *Proteins Struct. Funct. Bioinforma.* **79**, 1061–1078 (2011).
12. Jones, D. T., Buchan, D. W. A., Cozzetto, D. & Pontil, M. PSICOV: precise structural contact prediction using sparse inverse covariance estimation on large multiple sequence alignments. *Bioinformatics* **28**, 184–190 (2012).
13. Alford, R. F. *et al.* The Rosetta all-atom energy function for macromolecular modeling and design. 1–35 (2017) doi:10.1101/106054.
14. Greener, J. G., Kandathil, S. M. & Jones, D. T. Deep learning extends de novo protein modelling coverage of genomes using iteratively predicted structural constraints. *Nat. Commun. 2019 101* **10**, 1–13 (2019).
15. Gligorijević, V. *et al.* Structure-based protein function prediction using graph convolutional networks. *Nat. Commun. 2021 121* **12**, 1–14 (2021).
16. Heinig, M. & Frishman, D. STRIDE: a web server for secondary structure assignment from known atomic coordinates of proteins. *Nucleic Acids Res.* **32**, W500 (2004).
17. Viklund, H. & Elofsson, A. OCTOPUS: improving topology prediction by two-track ANN-based preference scores and an extended topological grammar. *Bioinformatics* **24**, 1662–8 (2008).
18. Hayat, S. & Elofsson, A. BOCTOPUS: Improved topology prediction of transmembrane beta-barrel proteins. *Bioinformatics* **28**, 516–522 (2012).
19. Necci, M., Piovesan, D., Dosztanyi, Z. & Tosatto, S. C. E. MobiDB-lite: fast and highly specific consensus prediction of intrinsic disorder in proteins. *Bioinformatics* **33**, 1402–1404 (2017).
20. Jones, D. T. & Cozzetto, D. DISOPRED3: precise disordered region predictions with annotated protein-binding activity. *Bioinformatics* **31**, 857–863 (2015).

21. Sillitoe, I. *et al.* CATH: increased structural coverage of functional space. *Nucleic Acids Res.* **49**, D266–D273 (2021).
22. Rose, P. W. *et al.* The RCSB Protein Data Bank: views of structural biology for basic and applied research and education. *Nucleic Acids Res.* **43**, D345-56 (2015).
23. Zhang, Y. & Skolnick, J. TM-align: a protein structure alignment algorithm based on the TM-score. *Nucleic Acids Res.* **33**, 2302–2309 (2005).
24. Olechnovič, K. & Venclovas, Č. VoroMQA: Assessment of protein structure quality using interatomic contact areas. *Proteins* **85**, 1131–1145 (2017).
25. Jones, D. T. Do transmembrane protein superfolds exist? *FEBS Lett.* **423**, 281–5 (1998).
26. Wallin, E. & von Heijne, G. Genome-wide analysis of integral membrane proteins from eubacterial, archaean, and eukaryotic organisms. *Protein Sci.* **7**, 1029–38 (1998).
27. Wimley, W. C. The versatile b -barrel membrane protein. 404–411 (2003) doi:10.1016/S0959-440X(03)00099-X.
28. Ward, J. J., Sodhi, J. S., McGuffin, L. J., Buxton, B. F. & Jones, D. T. Prediction and Functional Analysis of Native Disorder in Proteins from the Three Kingdoms of Life. *J. Mol. Biol.* **337**, 635–645 (2004).
29. Xu, J. & Zhang, Y. How significant is a protein structure similarity with TM-score = 0.5? *Bioinformatics* **26**, 889 (2010).
30. Jumper, J. *et al.* Highly accurate protein structure prediction with AlphaFold. *Nat. 2021 5967873* **596**, 583–589 (2021).
31. Faisal, F. E. *et al.* GRAFENE: Graphlet-based alignment-free network approach integrates 3D structural and sequence (residue order) data to improve protein structural comparison. *Sci. Rep.* **7**, (2017).
32. Wardman, J. F., Bains, R. K., Rahfeld, P. & Withers, S. G. Carbohydrate-active enzymes (CAZymes) in the gut microbiome. *Nat. Rev. Microbiol. 2022* 1–15 (2022) doi:10.1038/s41579-022-00712-1.
33. Drula, E. *et al.* The carbohydrate-active enzyme database: functions and literature. *Nucleic Acids Res.* **50**, D571–D577 (2022).
34. Boraston, A. B., Bolam, D. N., Gilbert, H. J. & Davies, G. J. Carbohydrate-binding modules: fine-tuning polysaccharide recognition. *Biochem. J.* **382**, 769–781 (2004).
35. Miliara, X. *et al.* Structural insight into the TRIAP1/PRELI-like domain family of mitochondrial phospholipid transfer complexes. *EMBO Rep.* **16**, 824–835 (2015).
36. Shaw, A. *et al.* A Novel Combination of Two Classic Catalytic Schemes. *J. Mol. Biol.* **320**, 303–309 (2002).
37. Jiang, D. *et al.* Crystal structure of 1,3Gal43A, an exo-β-1,3-galactanase from Clostridium thermocellum. *J. Struct. Biol.* **180**, 447–457 (2012).
38. Xiao, Y., Ng, S., Hyun Nam, K. & Ke, A. How type II CRISPR–Cas establish immunity through Cas1–Cas2-mediated spacer integration. *Nat. 2017 5507674* **550**, 137–141 (2017).
39. Fujishiro, T., Shimada, Y., Nakamura, R. & Ooi, M. Structure of sirohydrochlorin ferrochelatase SirB: the last of the structures of the class II chelatase family. *Dalt. Trans.* **48**, 6083–6090 (2019).
