# Supplementary material for: Sequence-structure-function relationships in the microbial protein universe
Source: Nat Commun. 2023 Apr 26;14:2351. doi: 10.1038/s41467-023-37896-w (PMC10133388; doi:10.1038/s41467-023-37896-w)
Supplement: Supplementary file 3 — Description to Additional Supplementary Information [file 41467_2023_37896_MOESM3_ESM.pdf]

Description of supplementary data files

File name: Supplementary Data 1
Description: Excel file of MIP IDs for the novel fold clusters. These include false positives.

File name: Supplementary Data 2
Description: Visualizations of novel fold clusters

File name: Supplementary Data 3
Description: Structure-to-function examples: comparing functions for novel fold structural clusters

File name: Supplementary Data 4
Description: Function-to-structure examples: comparing structures for specific functions
