# Supplementary material for: Sequence-structure-function relationships in the microbial protein universe
Source: Nat Commun. 2023 Apr 26;14:2351. doi: 10.1038/s41467-023-37896-w (PMC10133388; doi:10.1038/s41467-023-37896-w)
Supplement: Supplementary file 5 — Supplementary Dataset 2 [file 41467_2023_37896_MOESM5_ESM.pdf]

Sequence-structure-function relationships in the microbial protein universe

Supplementary Data 2

Visualization of novel fold clusters

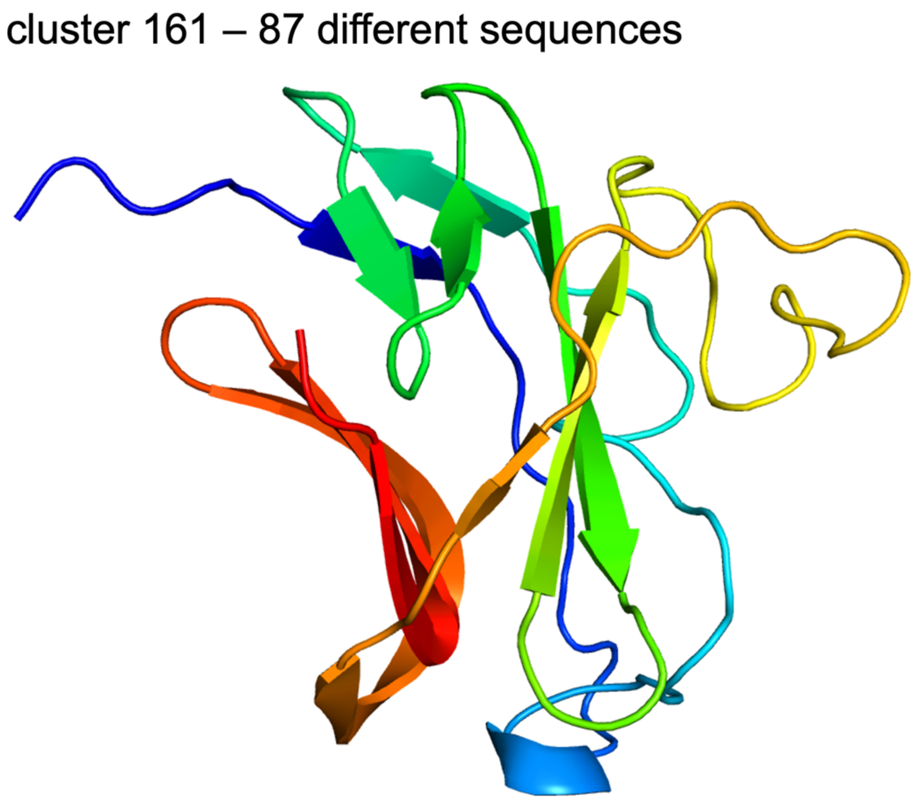

Fig. 1: Representative Rosetta models for all novel fold clusters (numbered).Figures with red background are hard false positives and with yellow background are soft false positives.

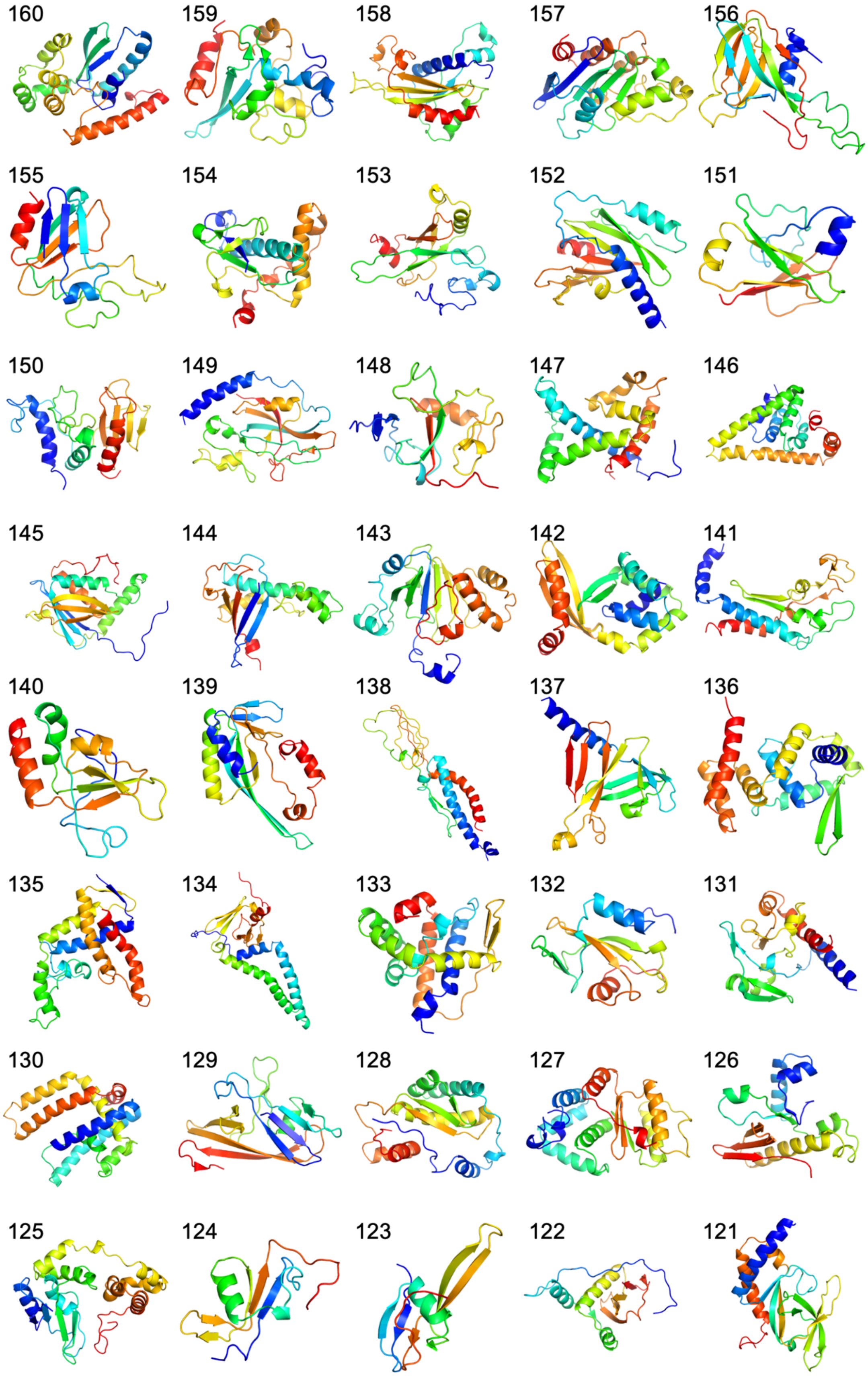

Fig. 1 continued

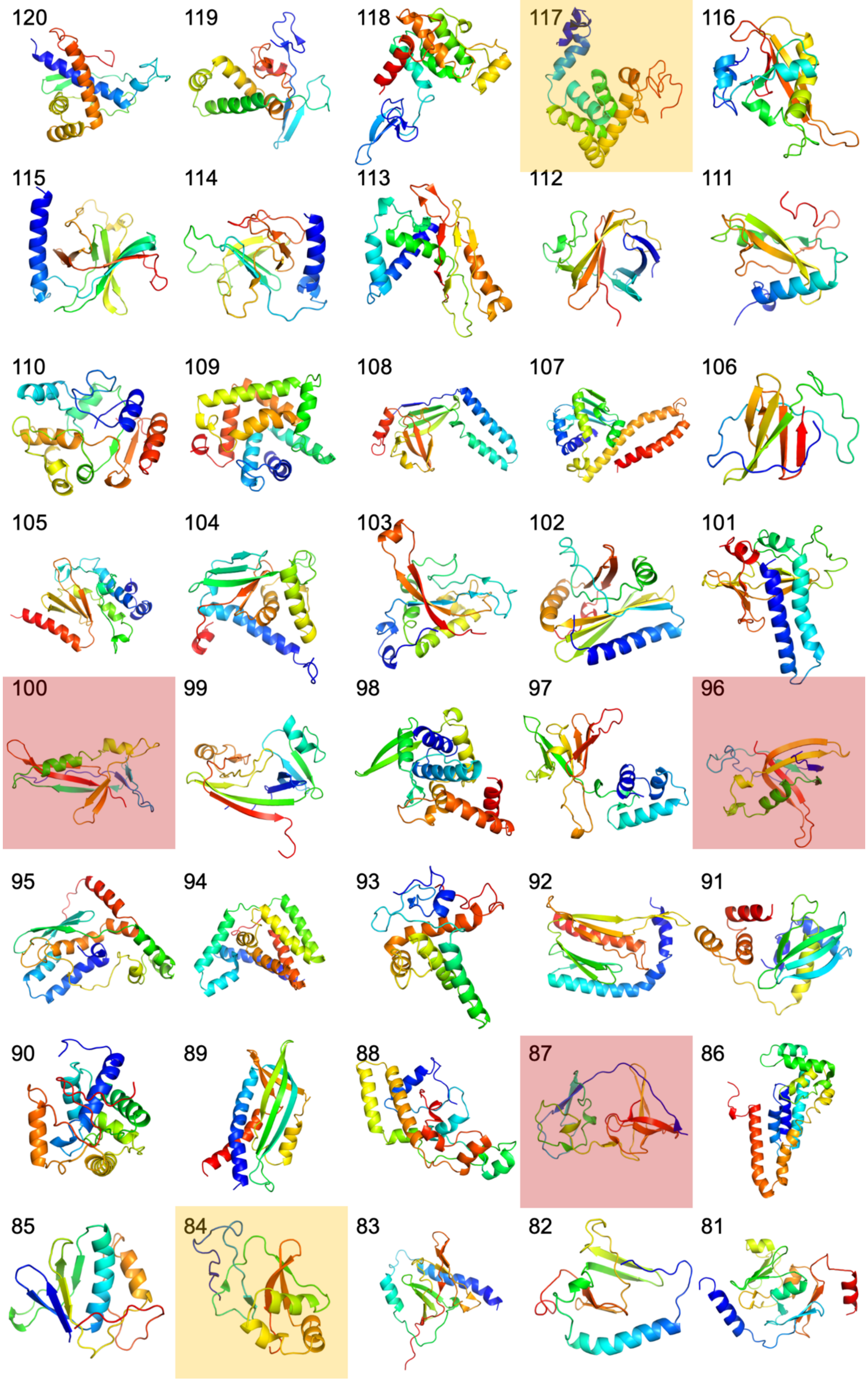

Fig. 1 continued

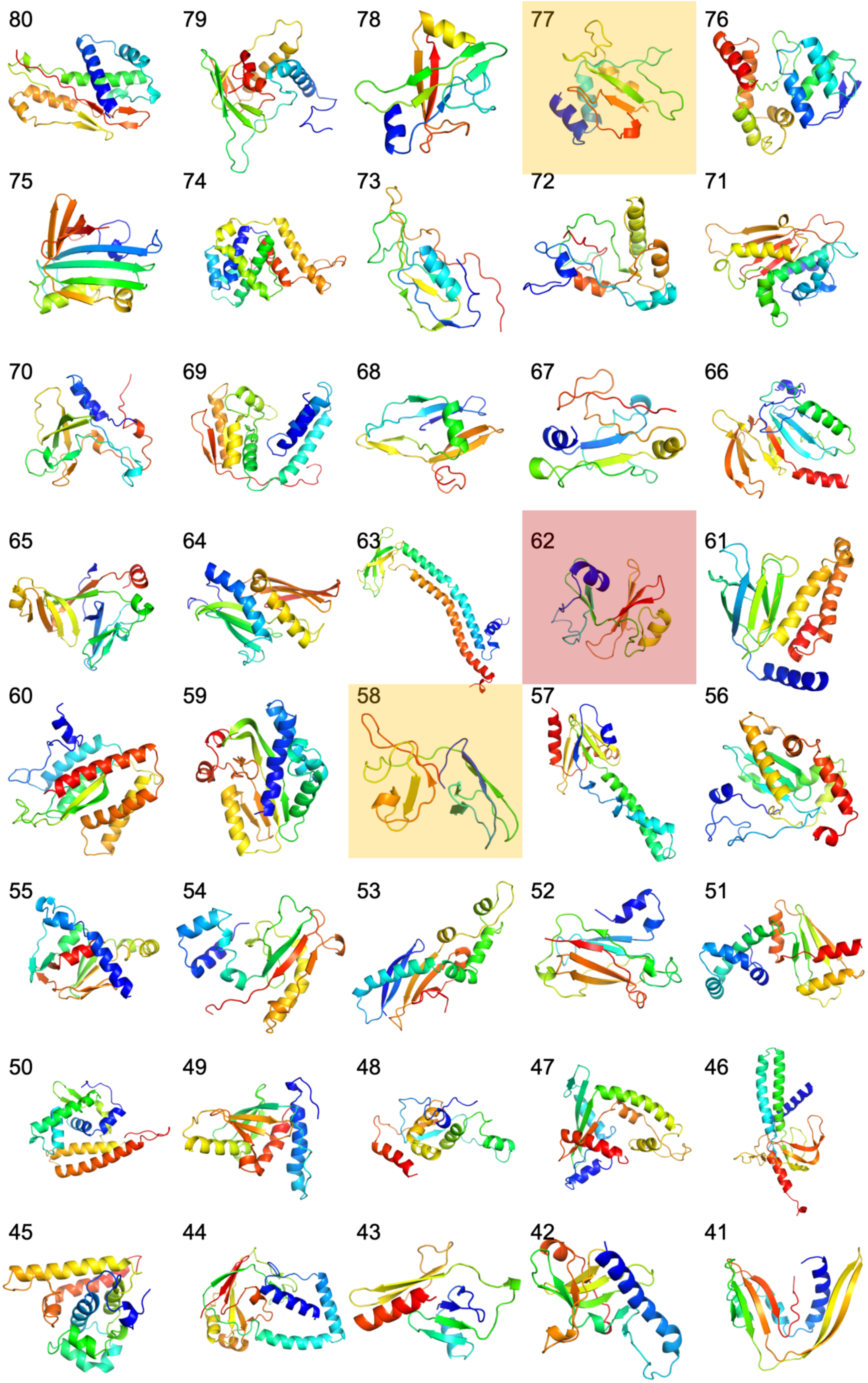

Fig. 1 continued

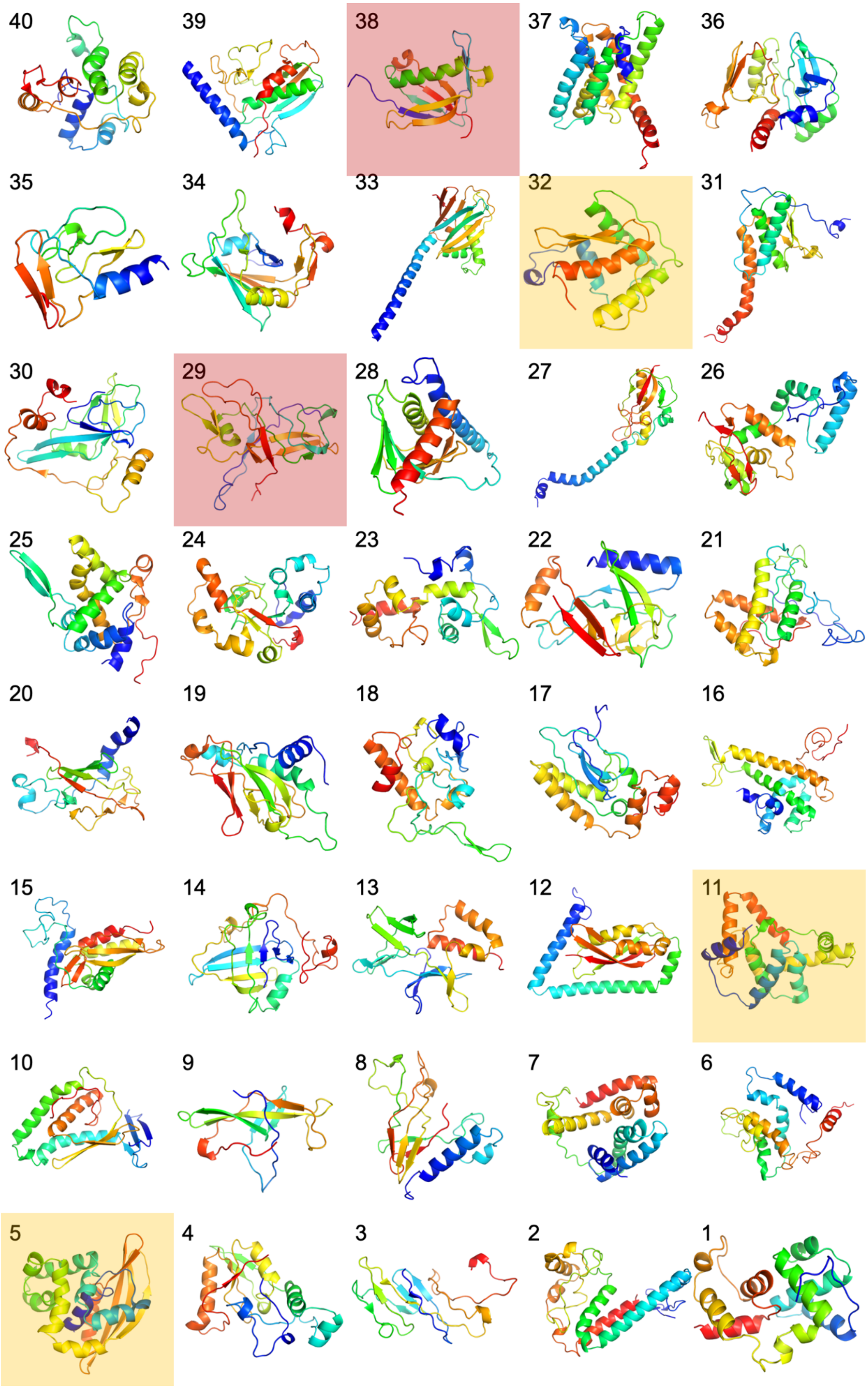

Fig. 1 continued
