# Supplementary material for: Sequence-structure-function relationships in the microbial protein universe
Source: Nat Commun. 2023 Apr 26;14:2351. doi: 10.1038/s41467-023-37896-w (PMC10133388; doi:10.1038/s41467-023-37896-w)
Supplement: Supplementary file 6 — Supplementary Dataset 3 [file 41467_2023_37896_MOESM6_ESM.pdf]

Sequence-structure-function relationships in the microbial protein universe

Supplementary Data 3

Structure-to-function examples: comparing functions for novel fold structural clusters

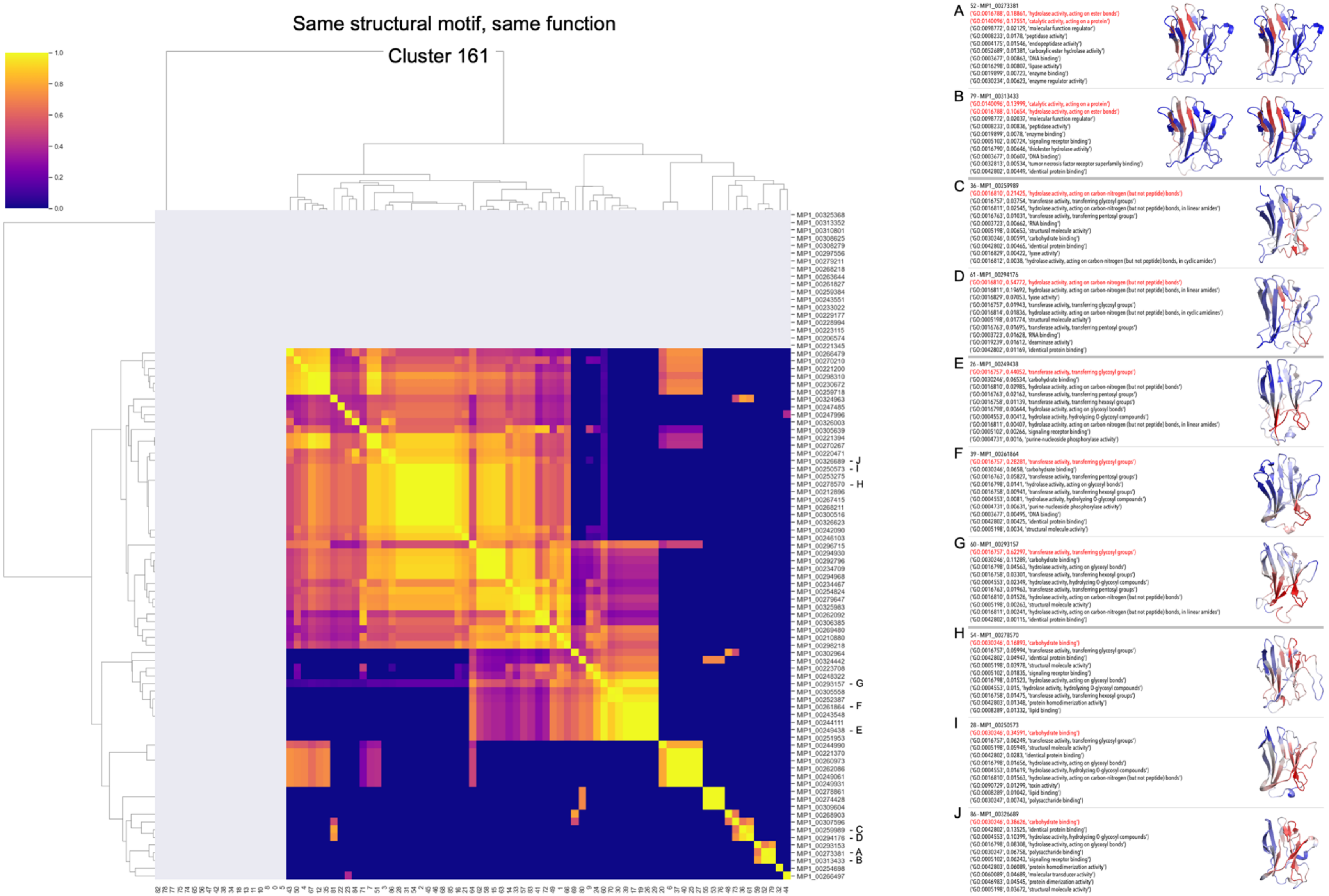

Fig. 1: Comparison between functions across proteins for the largest novel-fold cluster. The 452 proteins with previously unseen structures were clustered into 161 folds. Here we show the largest of the structural clusters, which has 87 representatives. The heatmap on the left shows the functional similarity (cosine similarity of function vectors) between protein pairs in this cluster. The majority of proteins (in this cluster and in general) follow the well-known observation that similar structures generate similar functions. When mapping residues-specific functions onto the protein structures (residues responsible for the function in red), one can see that different structural motifs are responsible for different functions - panel on the right.

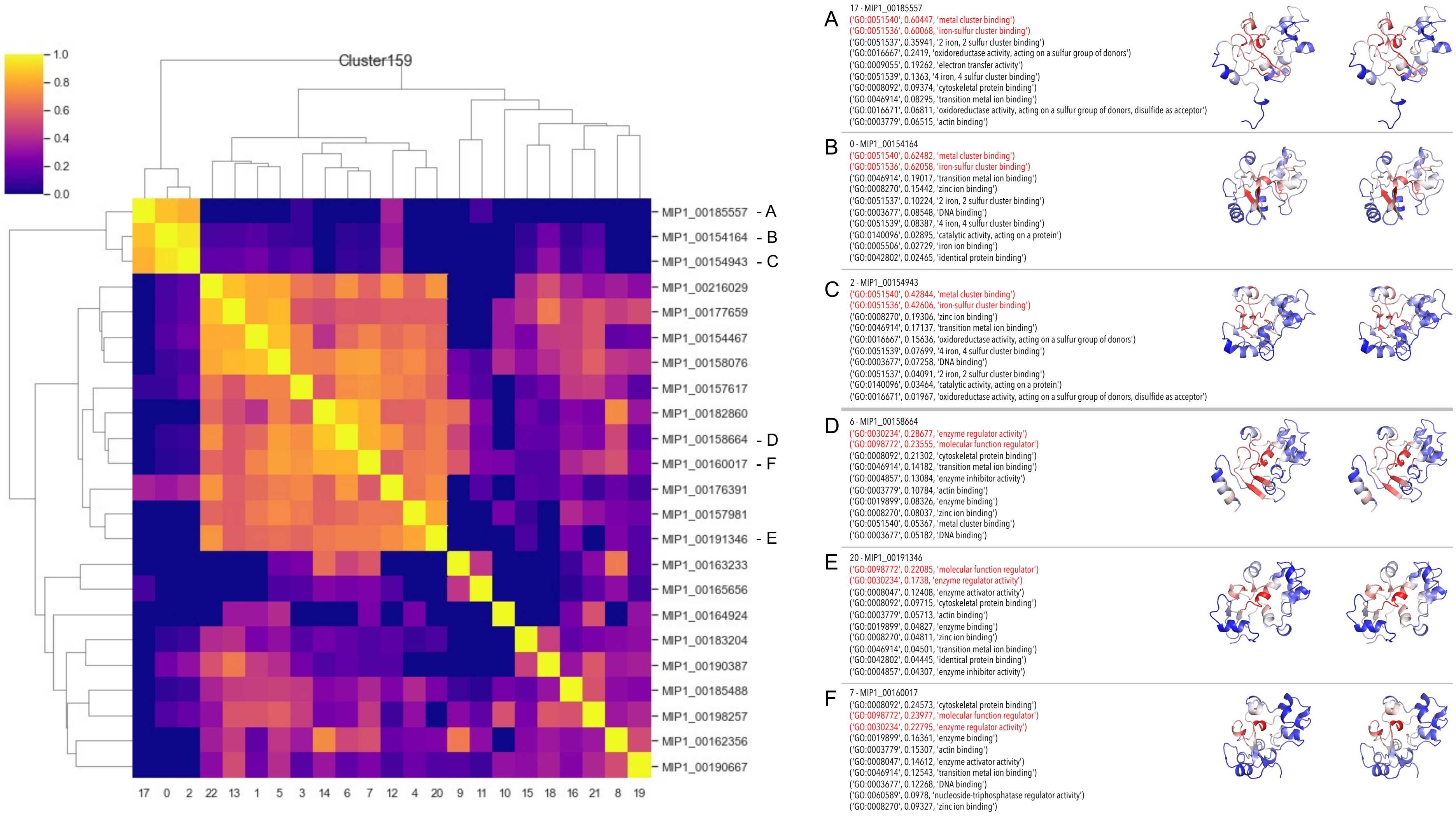

Fig. 2. Cluster159 with examples of similar structural motifs that produce similar functions. A, B, and C regulate iron-sulfur cluster binding which is a child term of metal cluster binding, and D, E, and F have molecular function regulator activity which includes enzyme regulator activity.

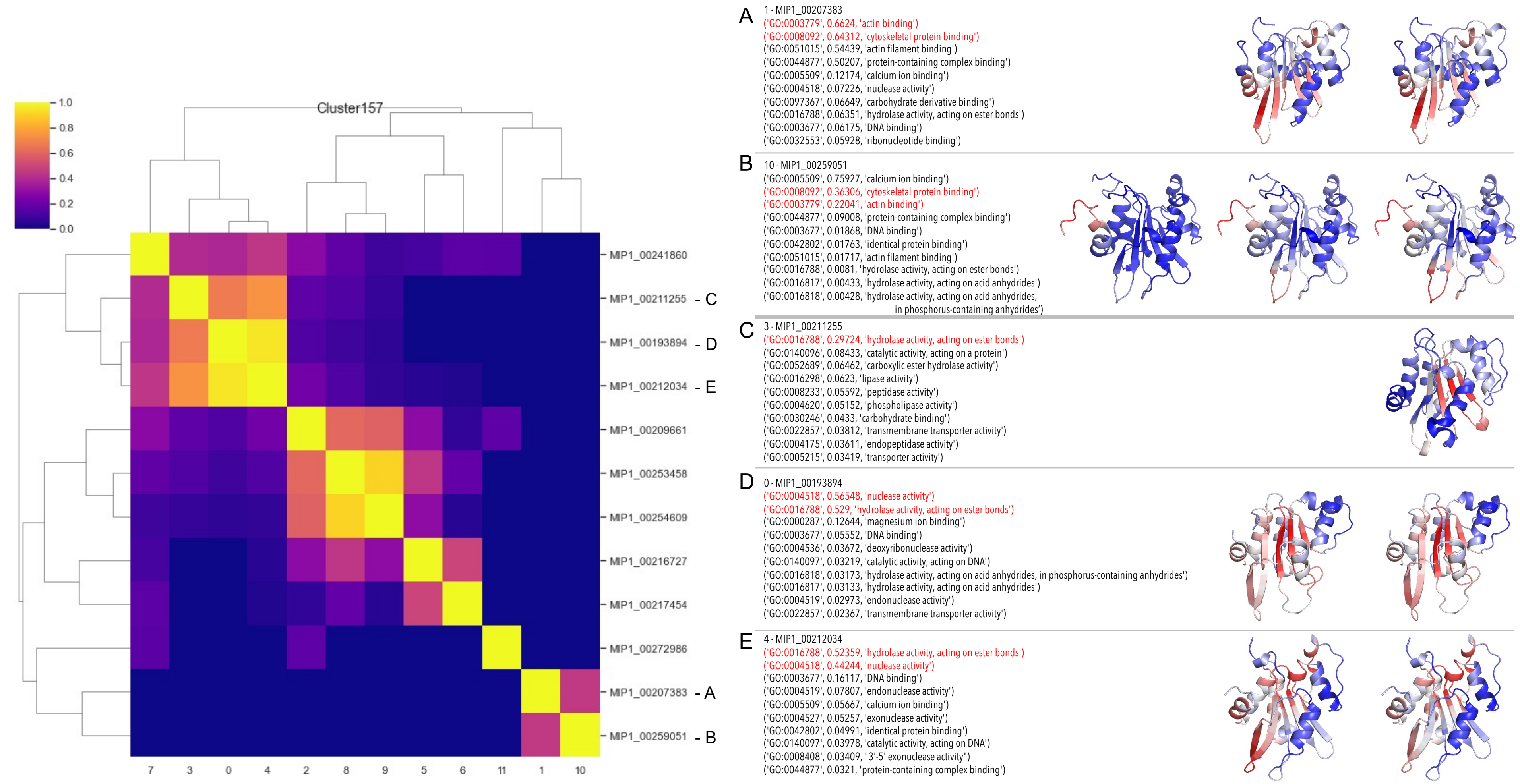

Fig. 3. Cluster 157 with examples of structure-function disparity. For A and B the same functions (cytoskeletal protein binding, which includes actin binding) can be produced by different structural motifs. For A, D and E the same structural motif produce different functions (cytoskeletal protein binding / actin binding and hydrolase activity, acting on ester bonds / nuclease activity).

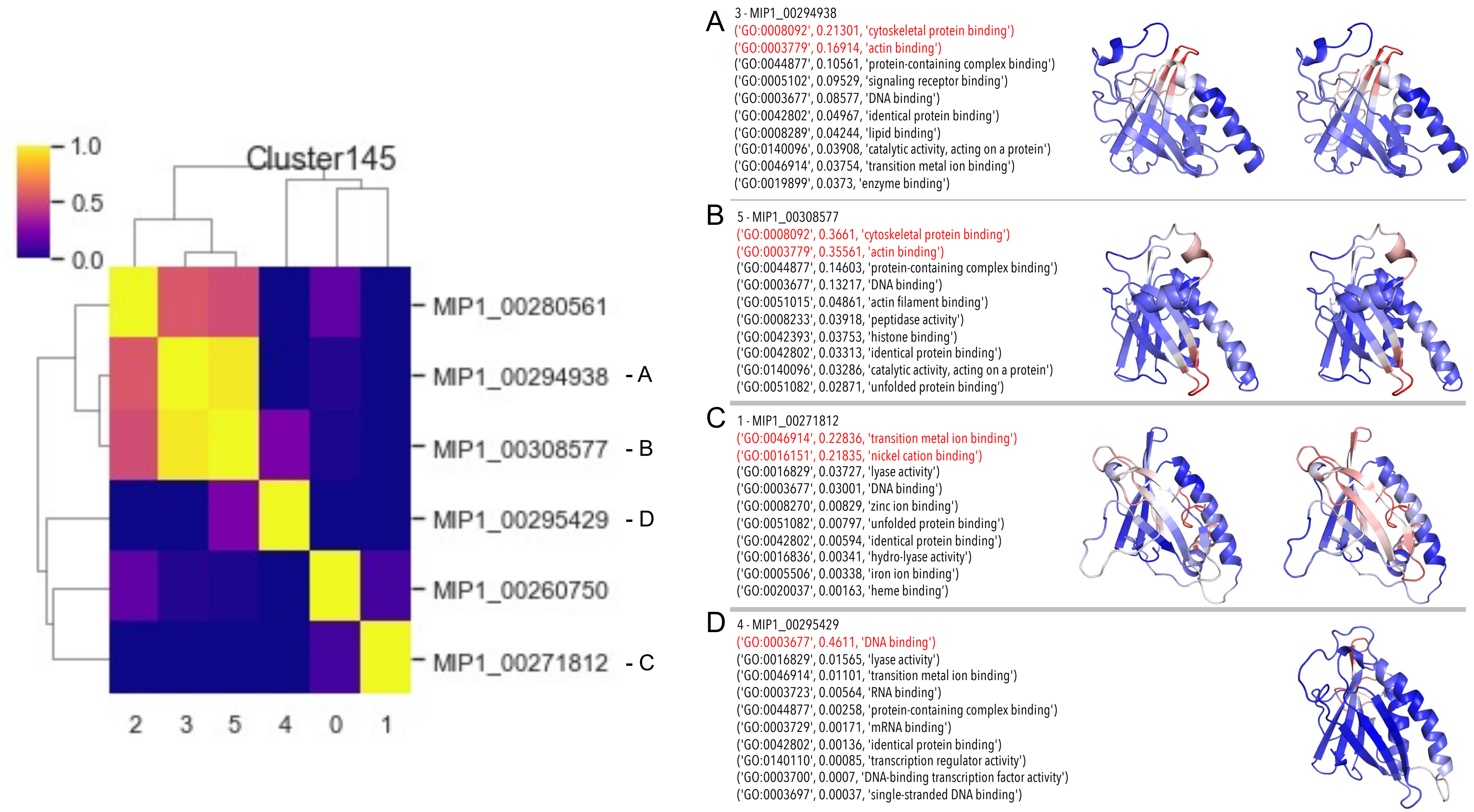

Fig. 4. Cluster 145 with examples of different structural motifs producing similar functions. In A and B, cytoskeletal protein binding, specifically actin binding, are produced by a different β-turn in the structure.

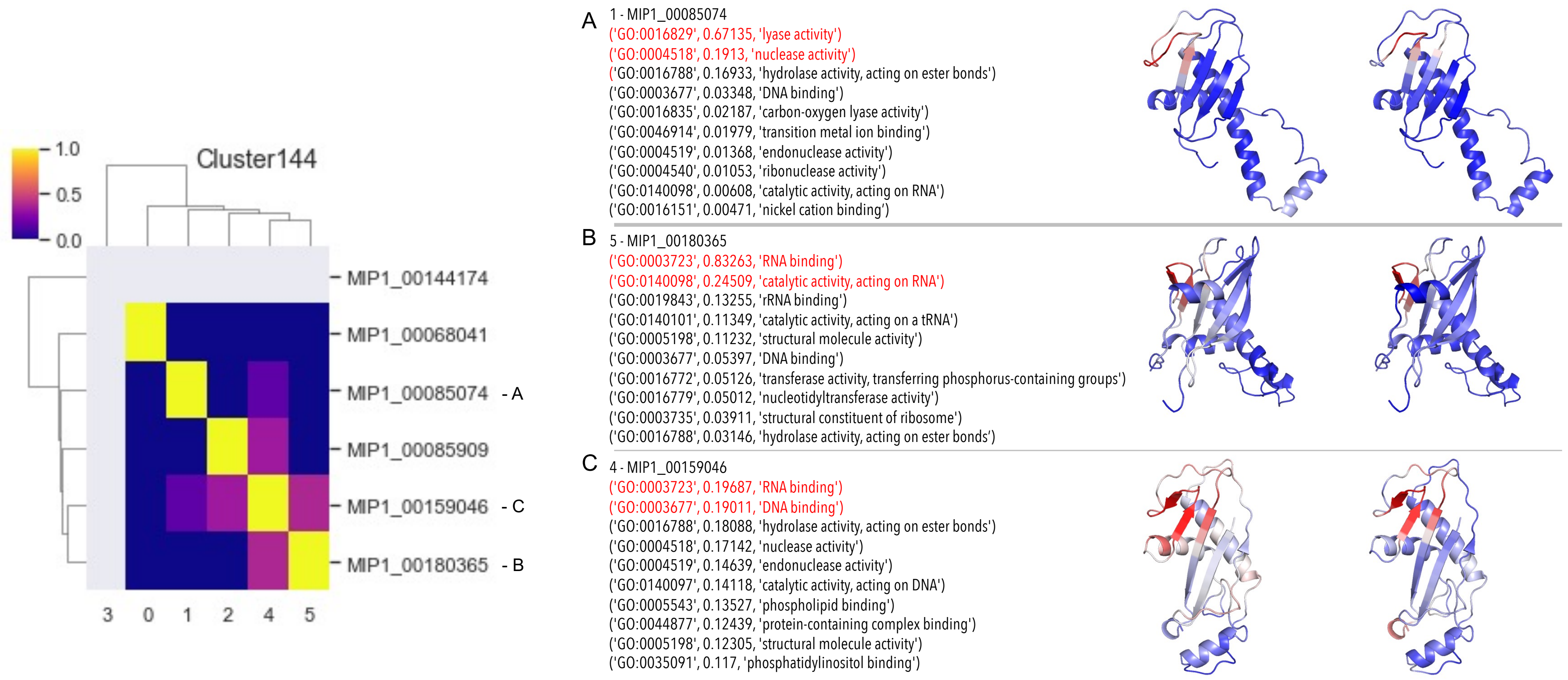

Fig. 5. Cluster 144 with examples of similar structural motifs producing different functions. In A, the β-turn produces lyase and nuclease activity, whereas in B and C, the same turn is responsible for RNA and DNA binding.

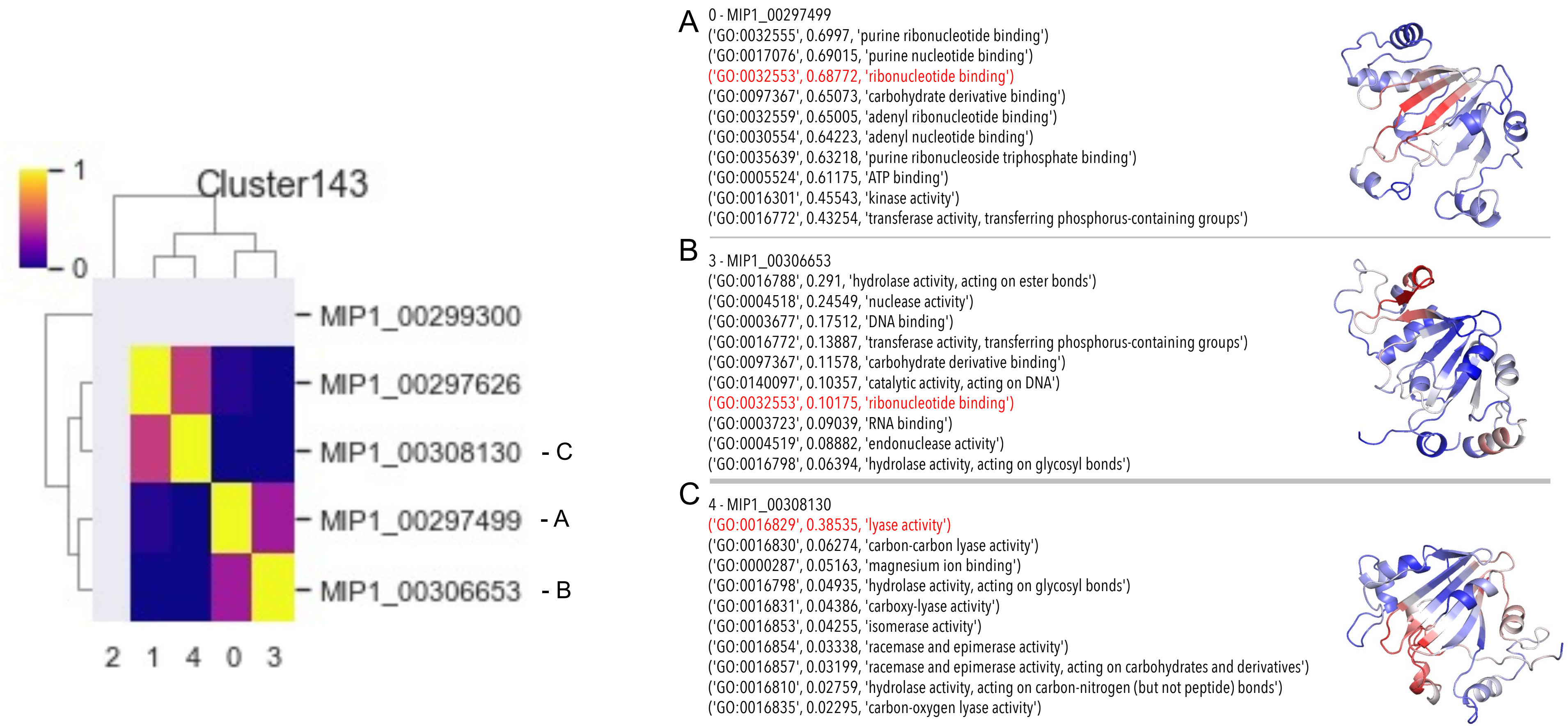

Fig. 6. Cluster 143. (A) and (B) have overlap in the structural motif that produces the same function (ribonucleotide binding). (C) shows a different function (lyase activity) that is produced by a different structural motif.

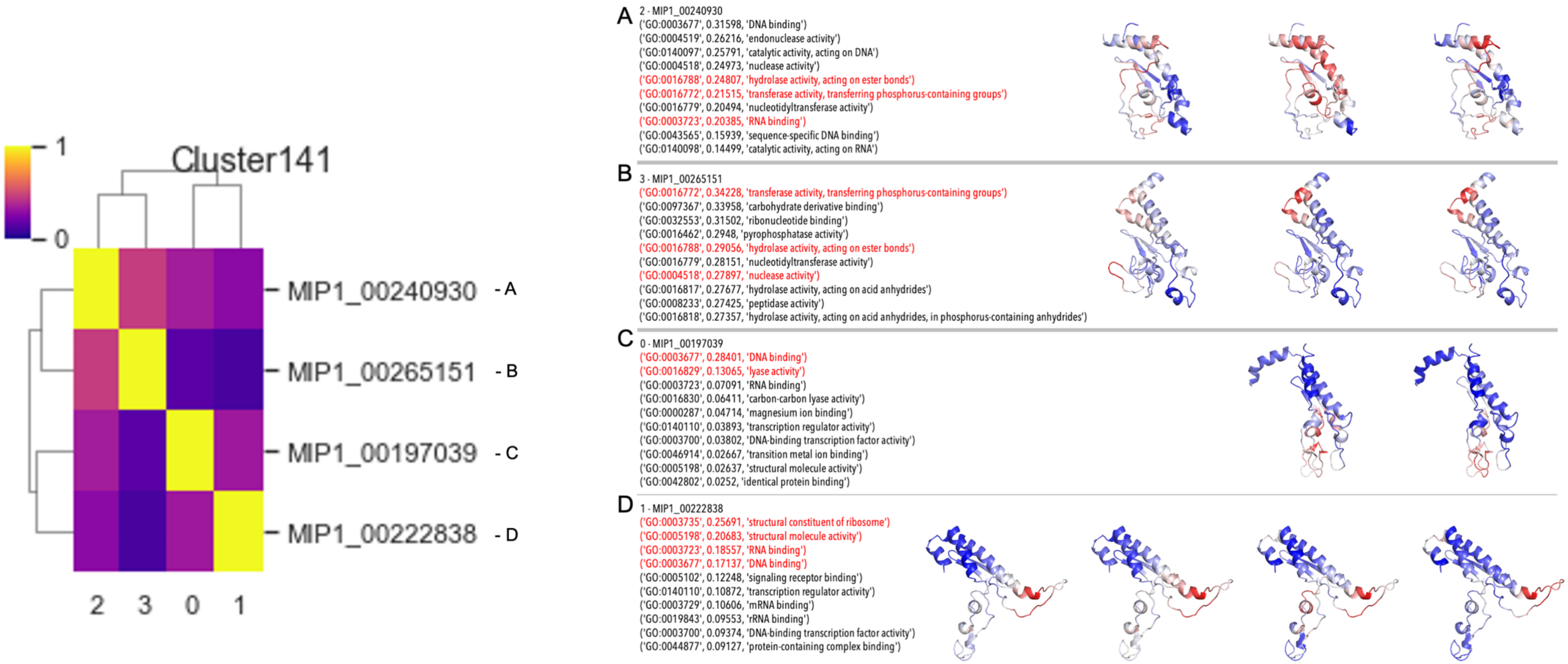

Fig. 7. Cluster 141 includes examples where overlapping structural motifs having the same function. Transferase activity in (A) and (B) are carried out by some of the same residues in the structure and DNA binding in (C) and (D) are carried out by the same structural motif.

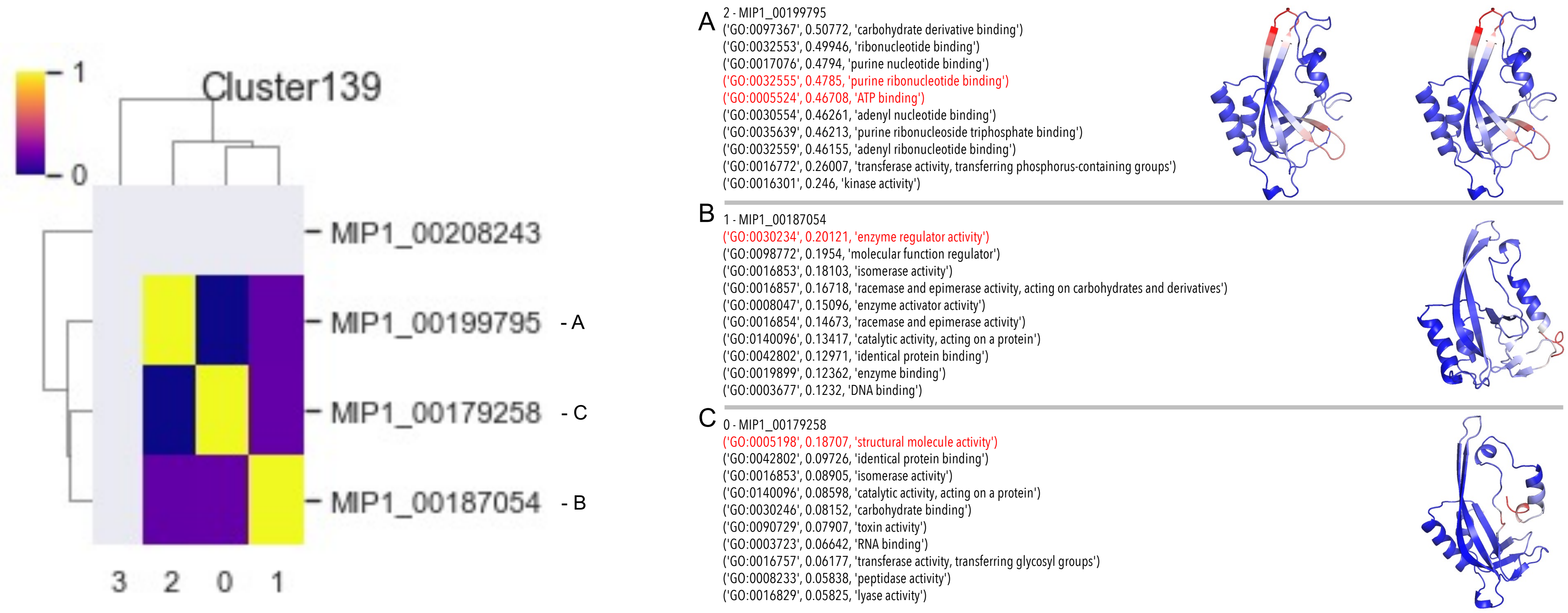

Fig. 8. Cluster 139 contains examples have low functional similarity. The termini in (B) and (C) have either enzyme regular activity or structural molecule activity.

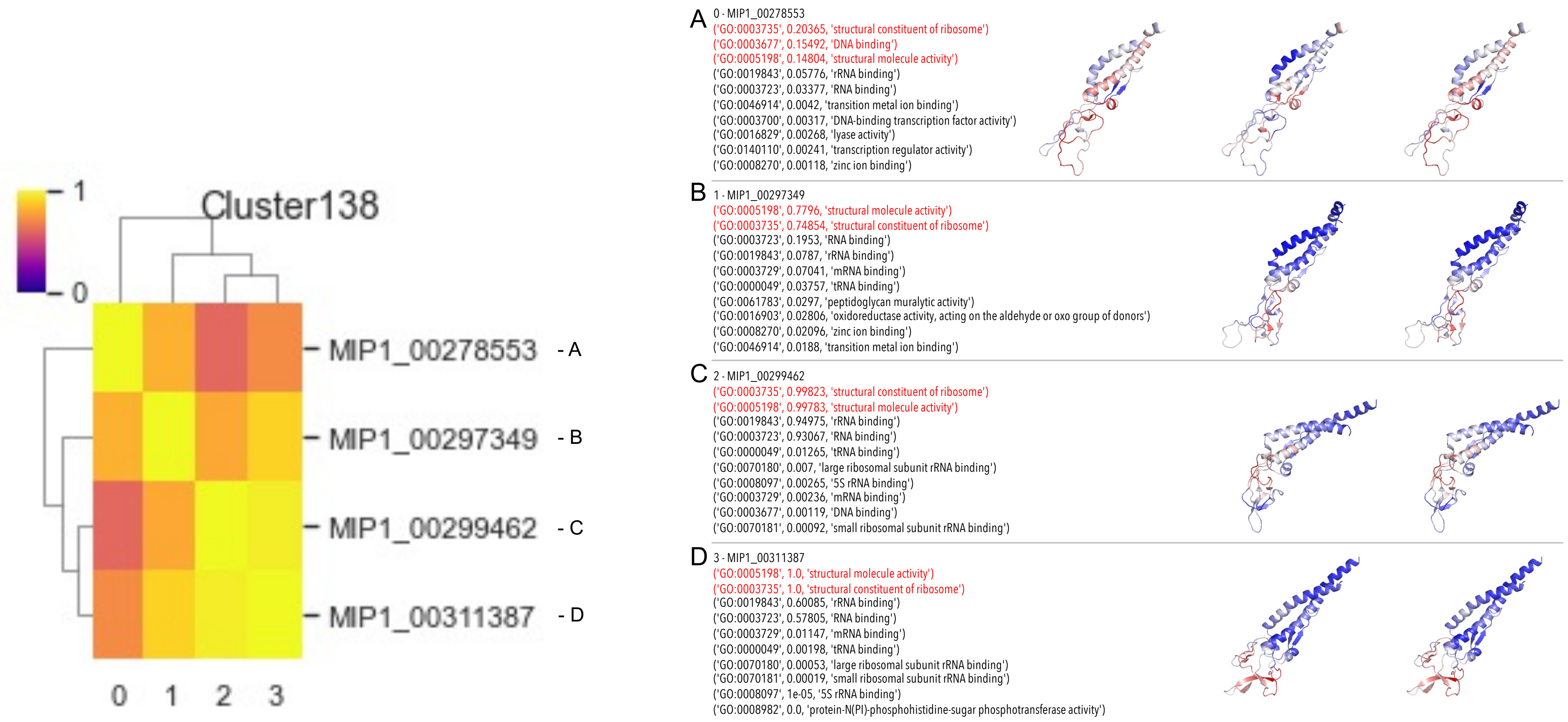

Fig. 9. Cluster 139 with examples of similar structural motifs producing similar functions. While the two helices show functional activitity in some examples, the loop region consistently has structural molecule activity.

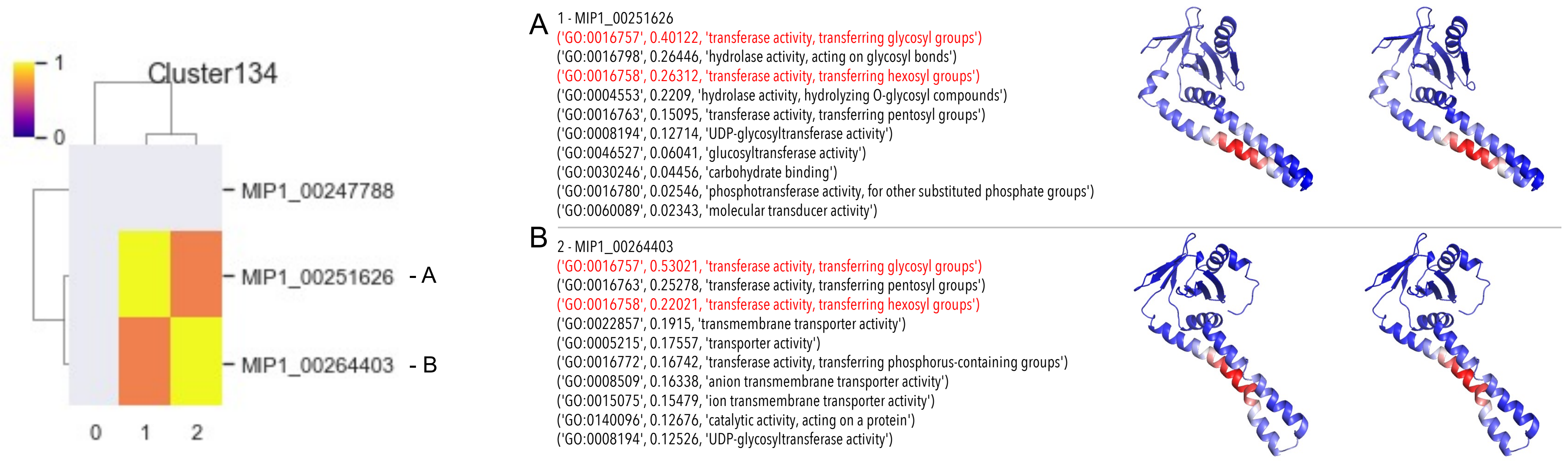

Fig. 10. Cluster 134. While both proteins have an overlap of functions as shown in the heatmap, the function transferase activity, transferring glycosyl or hexosyl groups pertains to the same structural motif, i.e. the center of one of the helices.

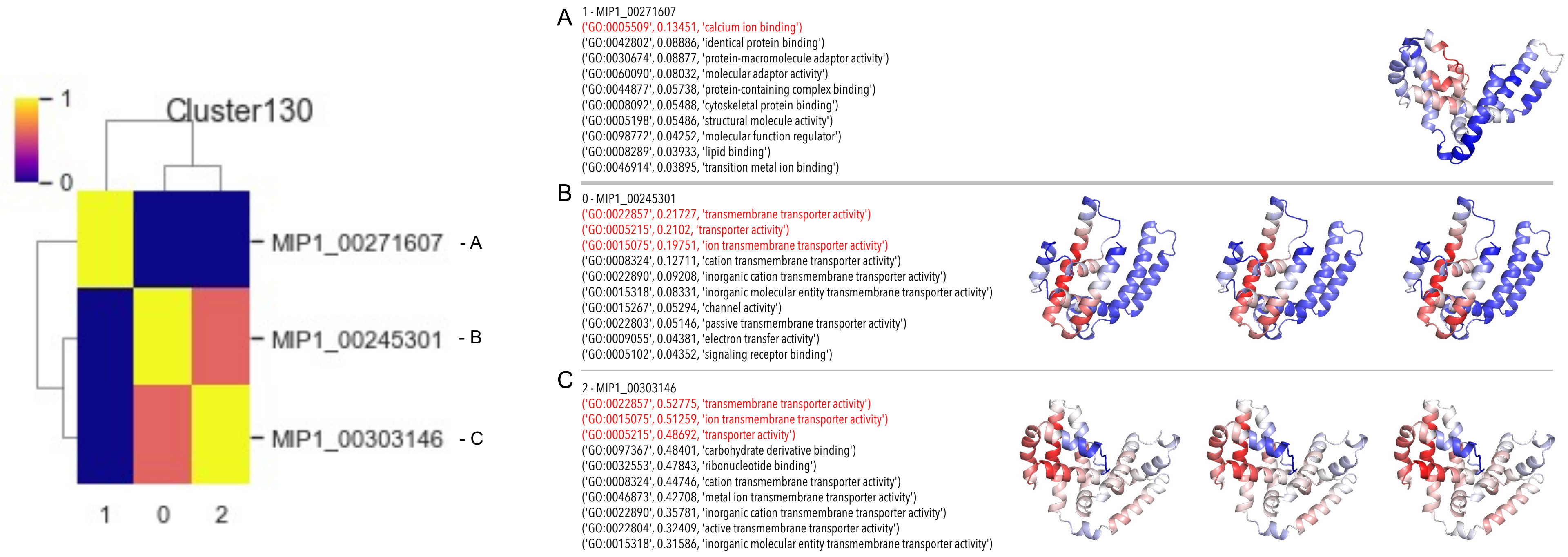

Fig. 11. Cluster 130. The protein in (A) has mostly different functions than (B) and (C) and the latter two have overlapping structural motifs that generate the functions of transmembrane transporter activity.

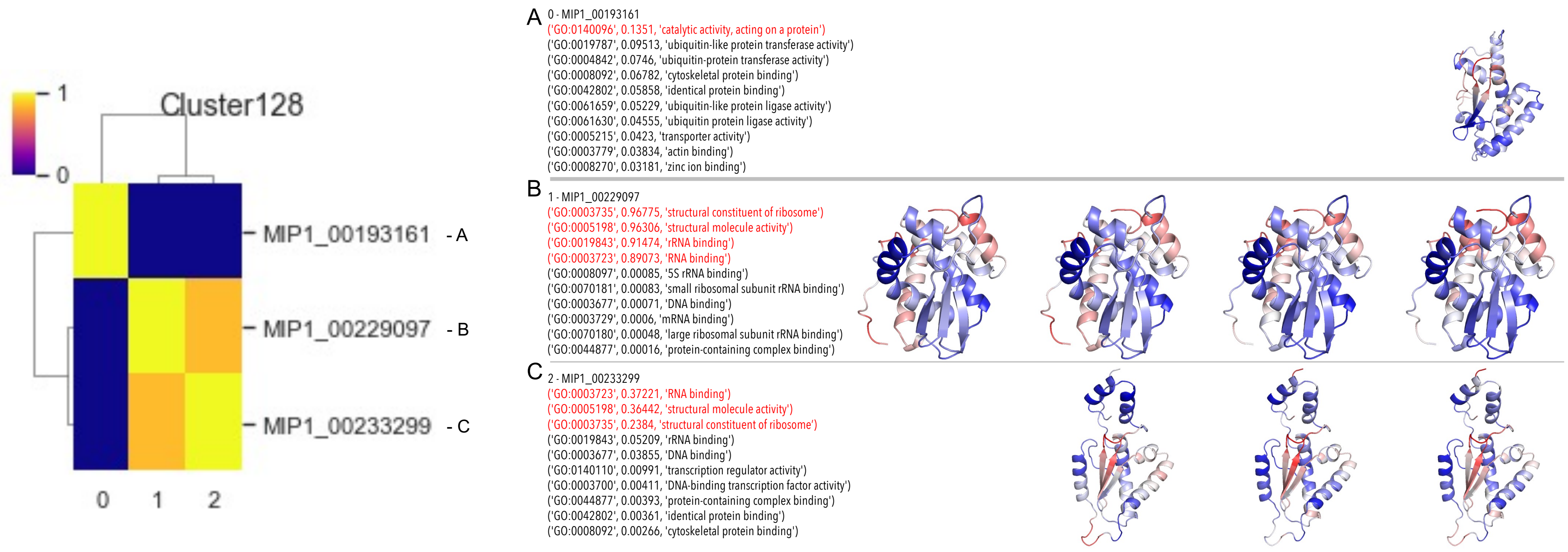

Fig. 12. Cluster 128. The functions of (A) are very different than those of (B) and (C). For the latter two, the same functions are created by different structural motifs. Further, the superposition is poor because the N- and C-termini have different interfaces to the core of the fold.
