# Supplementary material for: Sequence-structure-function relationships in the microbial protein universe
Source: Nat Commun. 2023 Apr 26;14:2351. doi: 10.1038/s41467-023-37896-w (PMC10133388; doi:10.1038/s41467-023-37896-w)
Supplement: Supplementary file 7 — Supplementary Dataset 4 [file 41467_2023_37896_MOESM7_ESM.pdf]

Sequence-structure-function relationships in the microbial protein universe

Supplementary Data 4

Function-to-structure examples: comparing structures for specific functions

BP GO:0030683 - mitigation of host immune response by virus

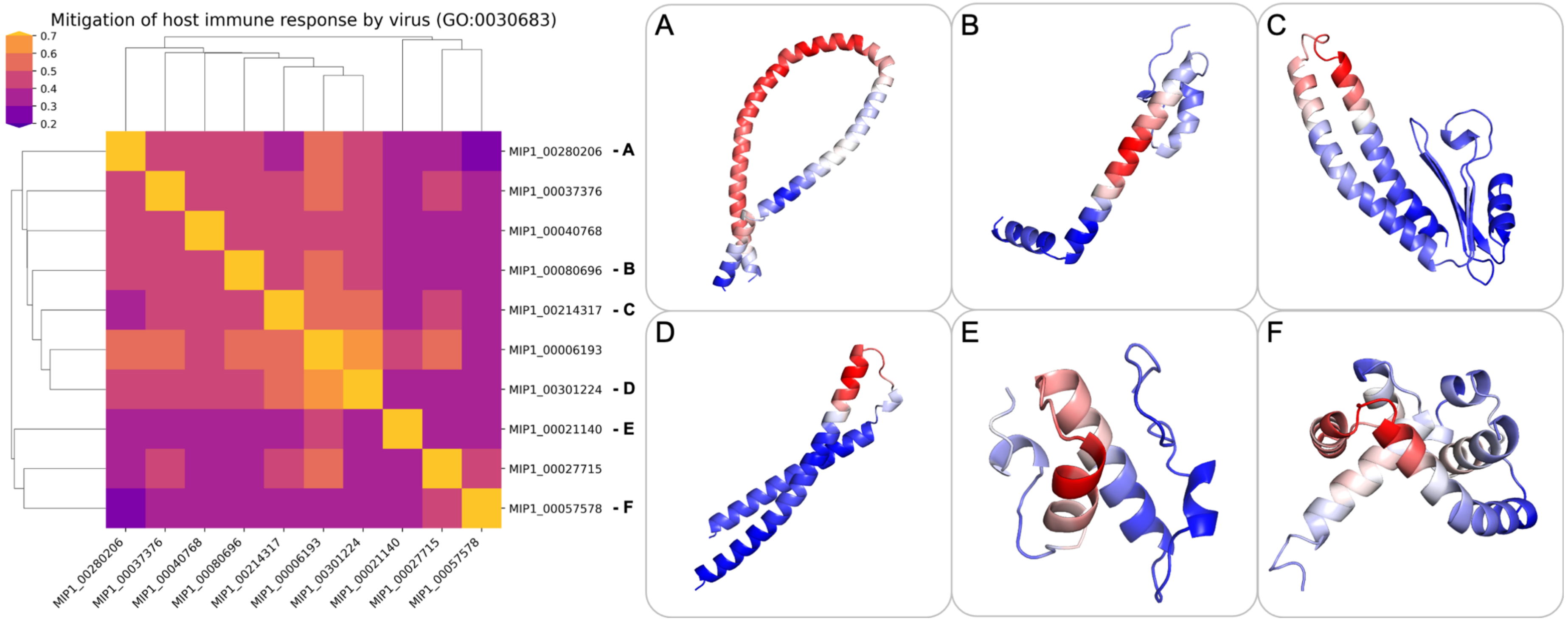

Fig. 1: The figure shows a functional cluster with a heatmap of pairwise TM-scores of the structures in that cluster. The proteins in that cluster evade the host immune response by a virus; this GO-term has six child terms. The PDB has over 6,000 entries with the GO-term, many of which are virus capsid proteins or proteases. The structures in this cluster have very different folds but most often contain two helices. We suspect that different folds generate this function because the function is very general.

EC 2.7.10.- Protein tyrosine kinases

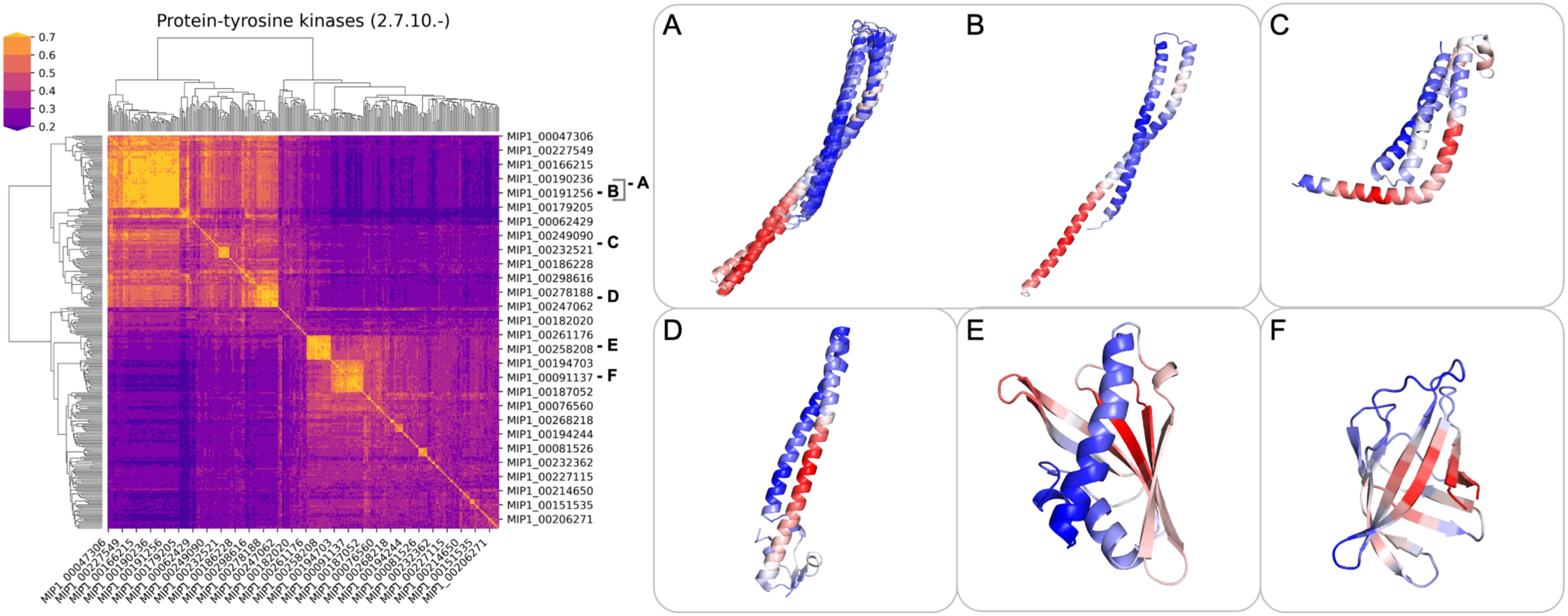

Fig. 2: The figure shows a functional cluster with a heatmap of pairwise TM-scores of the structures in that cluster. The heatmap shows that the functional cluster is divided into two groups, predominantly helical, denoting the upper left yellow square and covering (A), (B), (C), and (D), and with predominantly β-sheet content, denoted by the cluster in the lower right and covering models (E) and (F). This structural diversity is corroborated by the generality of this particular function: Tyrosine kinases are a large and diverse group of enzymes that are involved in many key events in the body. They catalyze the reaction of transferring a phosphate group from ATP to tyrosine residues of specific proteins, essentially switching them "on" or "off". Phosphorylation of tyrosine residues in proteins controls functions such as subcellular localization, enzyme activity, and signal transduction. Tyrosine kinases come in two forms: transmembrane receptor tyrosine kinases (RTKs) and cytosolic non-receptor tyrosine kinases. RTKs are signalizing molecules that homo- and hetero-dimerize in the membrane and transduce the signal from outside of the cell to the intracellular region. RTKs also have a number of domains that dictate to which family they belong. Mutations in RTKs are implicated in a variety of cancers, making RTKs important drug targets.

EC 4.2.1.1 - carbonic anhydrase

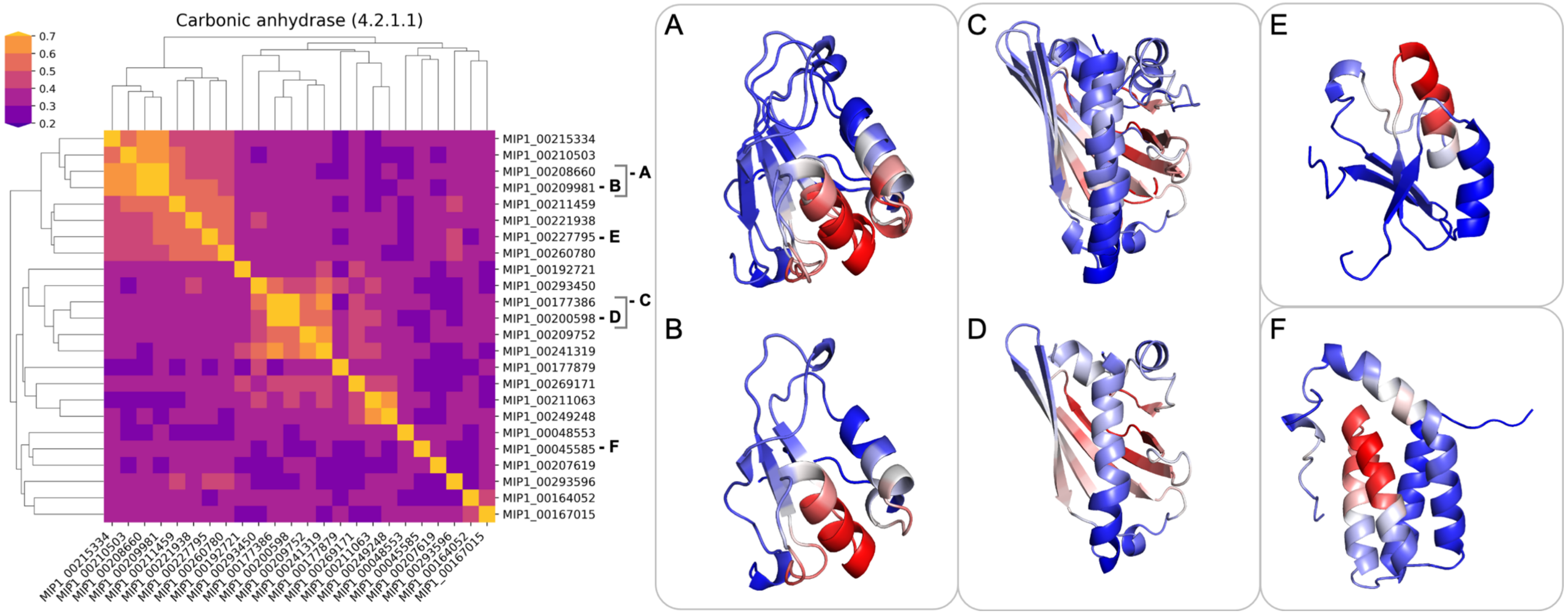

Fig. 3: The figure shows a functional cluster with a heatmap of pairwise TM-scores of the structures in that cluster. In our MIP dataset, carbonic anhydrases are a smaller class of proteins with three small clusters, covering several different folds. Carbonic anhydrases are enzymes that catalyze the conversion of carbon dioxide to carbonic acid, often with the help of zinc in the active site, making them metalloenzymes. Carbonic anhydrases help regulate pH and fluid balance. Three main families (α, β, γ) exist in addition to smaller families (δ, ζ, η, ι) that are less well-studied: α-CAs occur in mammals, β-CAs in bacteria and plants, and γ-CAs in methanogen bacteria in hot springs. The families are structurally different and appear to have evolved independently, which supports the structural diversity in this functional cluster in our MIP database. Interestingly, the fold in (C) superimposes perfectly with the structures of pathogenesis related proteins (for instance PDBID 4c94) for a variety of allergens and also cytokinin-specific binding proteins (for instance PDBID 2flh). The two latter proteins have an additional strand at the C-terminus that extends the twisted beta-sheet, which is not present in the MIP models.

EC 2.7.1.21 - thymidine kinase

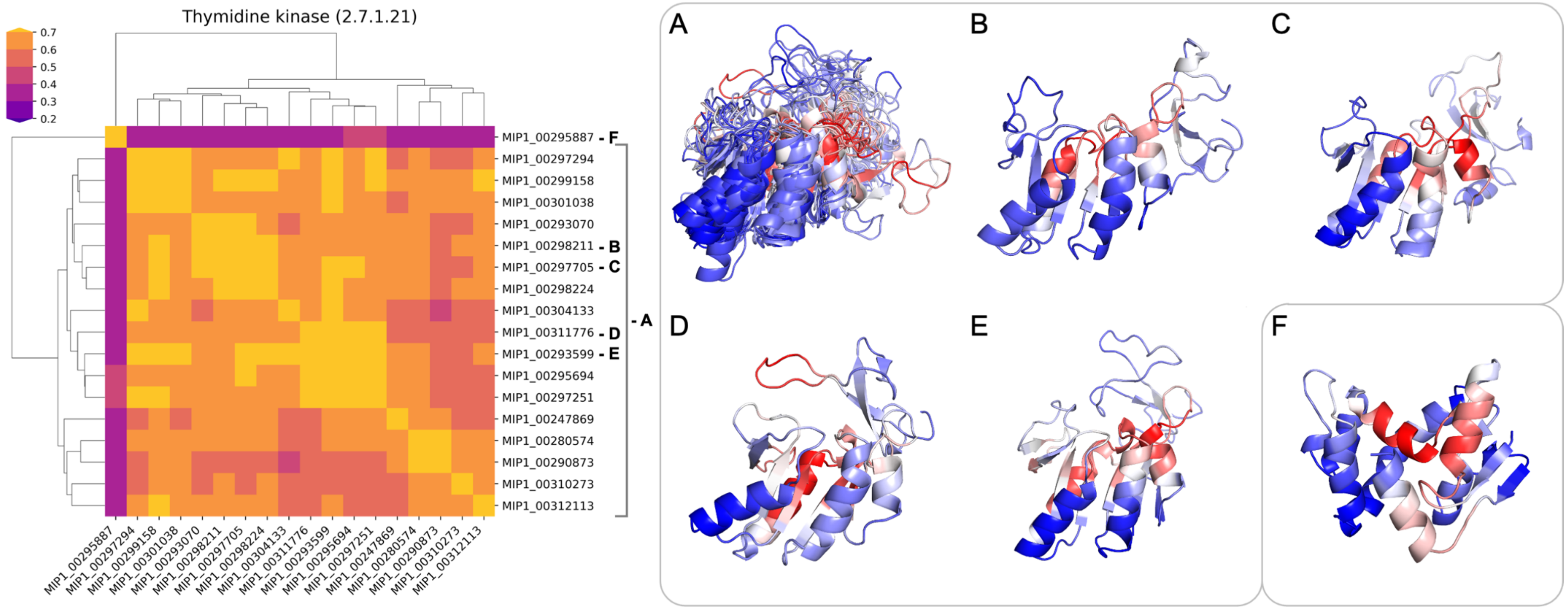

Fig. 4: The figure shows a functional cluster with a heatmap of pairwise TM-scores of the structures in that cluster. The functional cluster shows one main fold (A) and one minor fold with a single representative (F). The salient residues in cluster (A) overlay nicely for all representatives and have similar sequence motifs for this cluster with sequences of GKST[SLIH]LL / [LFI][LIVC]DEAQFL / G[LI]RTD[FA]. Thymidine kinases are enzymes that catalyze the transfer of a phosphate group from ATP to thymidine, creating thymidine monophosphate and ADP. They are a key element in the synthesis of DNA, as they introduce thymidine into the DNA. There are two families, one found in herpesviruses, and one found in mammals, bacteria and viruses. The latter family encompasses two types, type I and type II, which differ structurally. The main cluster in the MIP dataset, shown in (A) superimposes almost perfectly with type II thymidine kinases, examples of which are PDB IDs 1w4r (human) and 2b8t (ureaplasma parvum). The structure is an alternating $(\beta\alpha)$x5(β) fold containing six strands and five helices, in addition to a small sheet embedded in loop regions at the C-terminus. The structure in (F) does not overlay with any known thymidine kinase structures, so it is possible that this might be a divergent fold.

MF GO:0005125 - cytokine activity

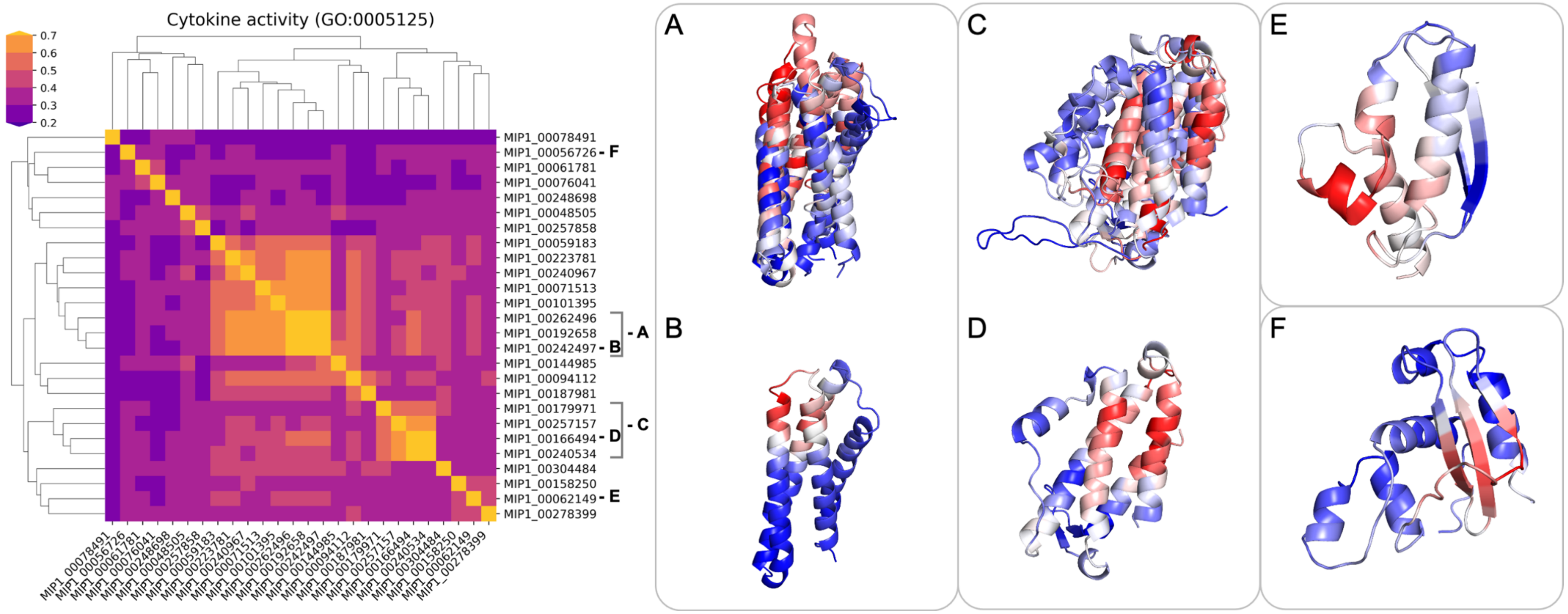

Fig. 5: The figure shows a functional cluster with a heatmap of pairwise TM-scores of the structures in that cluster. Cytokines are small proteins involved in cell signaling and they cannot cross the membrane bilayer. Rather, they modulate the function of the receptors they interact with to control growth, survival, differentiation and effector functions of cells and tissues. Cytokines are immune-modulating agents and are therefore important for the immune system, yet they are not limited to it. Cytokines include interleukins, chemokines, interferons, lymphokines and tumor necrosis factors but generally do not include hormones or growth factors. Their terminology overlaps with hormones and the distinction between the two is still being researched. Hormones are important cell signaling molecules that act distantly from the production site, but usually circulate in much higher concentrations than cytokines (nanomolar vs. picomolar concentrations). In our MIP dataset, the functional cluster with predicted cytokine activity contains 26 members, covering two fold clusters and several other folds. The covered folds are often helical bundles or have high helical content. Given that cytokine activity is a broad function carried out by various proteins, their structures cover a variety of folds. Our MIP models look overall similar to known folds for interleukin or interferon, yet their helix/strand connections are different.

MF GO:0009881 - photoreceptor activity

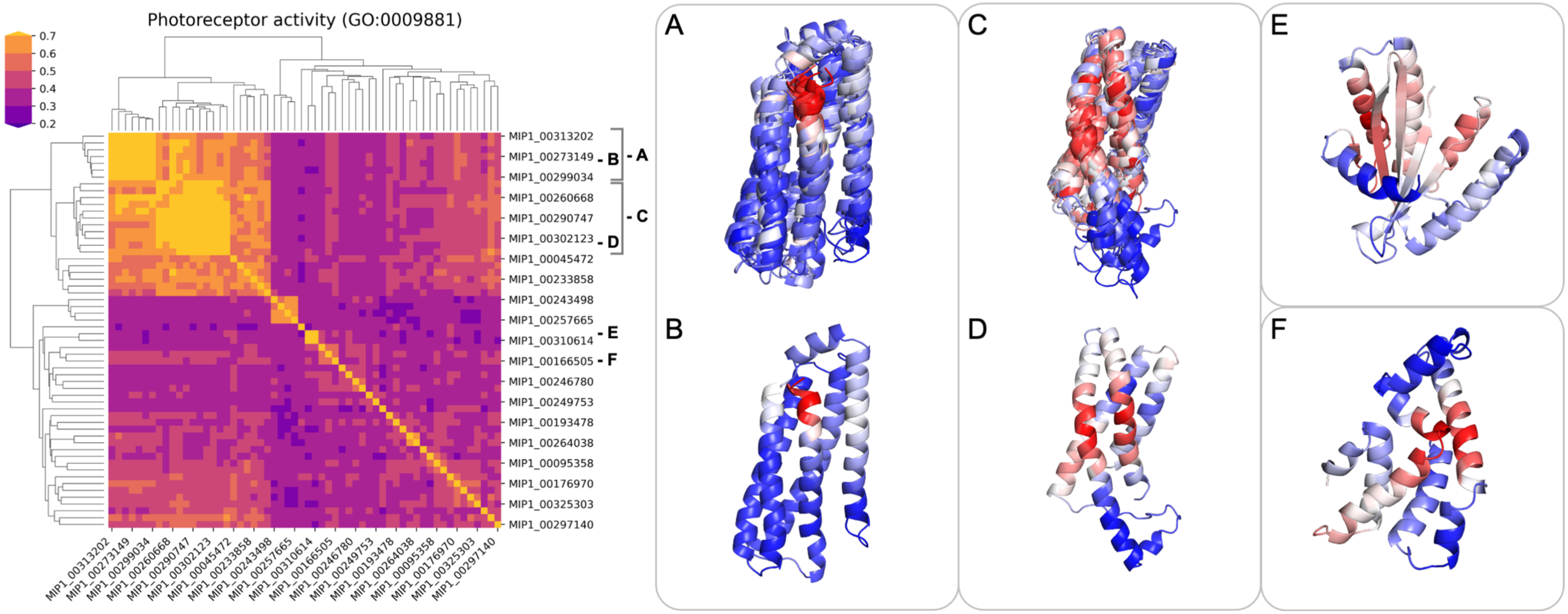

Fig. 6: The figure shows a functional cluster with a heatmap of pairwise TM-scores of the structures in that cluster. In our MIP database, the functional cluster with predicted photoreceptor activity is an intermediate-sized cluster that has one large structural cluster (splitting into two smaller ones for 4 and 5-helix bundles), few smaller clusters and several other folds. The vast majority of structures in this functional cluster consists of helical bundles, covering different folds. Note that bacteriorhodopsin folds would not be covered here because they are with ~300 residues larger than cutoff of protein sizes we have predicted structures for. The clusters utilize similar salient residues to accomplish their function. One exception in terms of fold is the structure in (E) with a $(\alpha\alpha\alpha\beta\beta)$x2 fold. This structure looks similar to small $\alpha\beta$ photoreceptor proteins like LOV (light-oxygen-voltage-sensing) domains and photoactive proteins, but the helix/strand connections differ, making them a different fold. Photoreceptor activity is the response to light and examples of proteins with photoreceptor activity include rhodopsin in the retina of vertebrae, phytochromes in plants, bacteriorhodopsins and bacteriophytochromes in some bacteria. Photoreceptors usually have a photopigment ligand that reacts to light and induces a conformational change in the ligand, for instance through isomerization, which in turn triggers a conformational change in the receptor, causing a signaling cascade. Examples of pigments include retinal, flavin and bilin, even though some proteins work without pigments.

MF GO:0004930 - G protein-coupled receptor activity

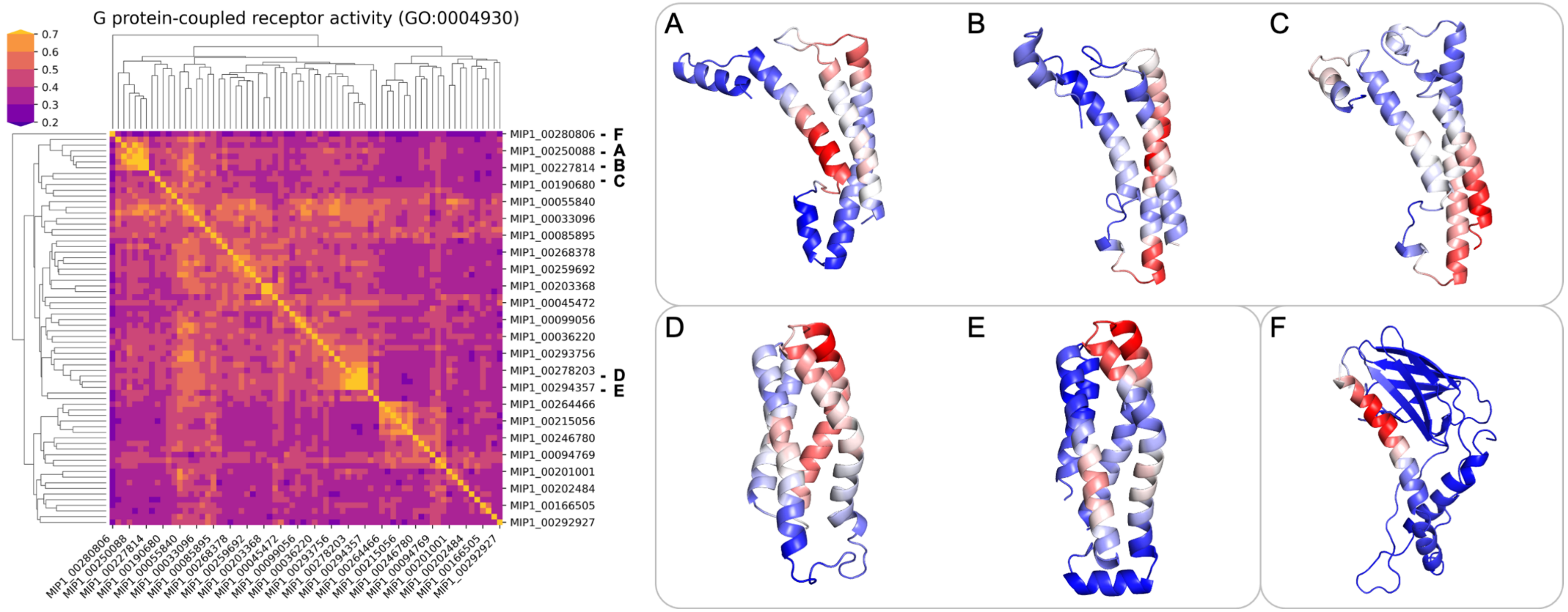

Fig. 7: The figure shows a functional cluster with a heatmap of pairwise TM-scores of the structures in that cluster. The function GPCR activity covers an intermediate-sized cluster with several smaller clusters. The covered folds are vastly different, albeit mostly small, helical bundles and with very little strand content. The typical GPCR 7-helix bundle is not represented because these folds are with ~300 residues larger than the sequence-length cutoff of 200 residues we chose. QuickGO defines GPCR activity as "Combining with an extracellular signal and transmitting the signal across the membrane by activating an associated G-protein; promotes the exchange of GDP for GTP on the alpha subunit of a heterotrimeric G-protein complex."
