# Supplementary material for: Sequence-structure-function relationships in the microbial protein universe
Source: Nat Commun. 2023 Apr 26;14:2351. doi: 10.1038/s41467-023-37896-w (PMC10133388; doi:10.1038/s41467-023-37896-w)
Supplement: Supplementary file 8 — Reporting Summary [file 41467_2023_37896_MOESM8_ESM.pdf]

nature portfolio

Corresponding author(s): Julia Koehler Leman, Tomasz Kosciolek

Last updated by author(s): Feb 28, 2023

Reporting Summary

Nature Portfolio wishes to improve the reproducibility of the work that we publish. This form provides structure for consistency and transparency in reporting. For further information on Nature Portfolio policies, see our Editorial Policies and the Editorial Policy Checklist.

Statistics

For all statistical analyses, confirm that the following items are present in the figure legend, table legend, main text, or Methods section.

n/a	Confirmed	
☐	☒	The exact sample size (*n*) for each experimental group/condition, given as a discrete number and unit of measurement
☒	☐	A statement on whether measurements were taken from distinct samples or whether the same sample was measured repeatedly
☒	☐	The statistical test(s) used AND whether they are one- or two-sided<br>*Only common tests should be described solely by name; describe more complex techniques in the Methods section.*
☐	☒	A description of all covariates tested
☐	☒	A description of any assumptions or corrections, such as tests of normality and adjustment for multiple comparisons
☐	☒	A full description of the statistical parameters including central tendency (e.g. means) or other basic estimates (e.g. regression coefficient) AND variation (e.g. standard deviation) or associated estimates of uncertainty (e.g. confidence intervals)
☒	☐	For null hypothesis testing, the test statistic (e.g. *F*, *t*, *r*) with confidence intervals, effect sizes, degrees of freedom and *P* value noted<br>*Give P values as exact values whenever suitable.*
☐	☒	For Bayesian analysis, information on the choice of priors and Markov chain Monte Carlo settings
☐	☒	For hierarchical and complex designs, identification of the appropriate level for tests and full reporting of outcomes
☐	☒	Estimates of effect sizes (e.g. Cohen's *d*, Pearson's *r*), indicating how they were calculated

Our web collection on statistics for biologists contains articles on many of the points above.

Software and code

Policy information about availability of computer code

Data collection	The Rosetta Macromolecular Modeling Suite, based on release 2016.32.58837, was used for protein structure prediction on the World Community Grid. DMPfold (downloaded September 2019) was used to predict the structures of all MIP sequences.

Data analysis	TM-align v.20190822 (https://zhanggroup.org//TM-align/) was used for computing TM-scores and sequence identities of aligned structures11. Structure visualizations were created in Pymol v.2.4.0 (https://github.com/schrodinger/pymol-open-source). Secondary structure assignments were generated using Stride v.2002102236. Alpha-helical transmembrane annotations were generated using OCTOPUS (as a part of TOPCONS2 software37; singularity image downloaded on July 17, 2020, dependencies: Blast v.2.2.26, Uniref90 v.20200119, Pfam 20191204). Beta-strand transmembrane annotations were generated using BOCTOPUS238 (zip downloaded on August 8, 2020; dependencies: HH-suite v.2.0.16, Blast v.2.2.26, Uniprot20 v.20160226). Absolute and relative contact order was computed from definition39. For disordered sequence identification we used MobiDB-lite40 v.1.0 (March 2016) and DISOPRED341 (zip downloaded on September 16, 2021; dependencies: Blast42 v.2.2.26, Uniref90 v.20210731). Putative new fold clusters were computed using Python package NetworkX v.2.7.1. For putative new fold verification, we used AlphaFold2 with "preset" flag set to full_pdb (repository downloaded on August 16, 2021; reference databases which includes the PDB downloaded on July 31, 2021). A cosmetically modified version of the Rosetta Macromolecular Modeling Suite43,44, based on release 2016.32.58837, was used for protein structure prediction on the World Community Grid. The fragment picking pipeline45 is also part of the standard Rosetta distribution. Both are obtainable from the Rosetta Commons (https://www.rosettacommons.org/). Residue-residue pair constraints were obtained using GREMLIN34 version 2.0.1. DMPfold9 v1.0 (https://github.com/psipred/DMPfold, downloaded September 2019) was used to predict the structures of all MIP sequences. All custom codes generated for this study are part of the Zenodo repository (url https://doi.org/10.5281/zenodo.6611431) and on Github at https://github.com/microbiome-immunity-project/protein_universe under commit ID 23354bf. This includes information on the directory structure and how to search the database via workflows and scripts using a query sequence, a query structure, or a query function, to find similar proteins in the MIP dataset.

For manuscripts utilizing custom algorithms or software that are central to the research but not yet described in published literature, software must be made available to editors and reviewers. We strongly encourage code deposition in a community repository (e.g. GitHub). See the Nature Portfolio guidelines for submitting code & software for further information.

Data

Policy information about availability of data

All manuscripts must include a data availability statement. This statement should provide the following information, where applicable:

- Accession codes, unique identifiers, or web links for publicly available datasets
- A description of any restrictions on data availability
- For clinical datasets or third party data, please ensure that the statement adheres to our policy

All sequence, structure and function data generated in the project, along with relevant metadata are deposited on Zenodo with the DOI 10.5281/zenodo.6477242 and on Github at https://github.com/microbiome-immunity-project/protein_universe. Information on the directory structure and how to search the database are available both on Zenodo.

Human research participants

Policy information about studies involving human research participants and Sex and Gender in Research.

Reporting on sex and gender	NA
Population characteristics	NA
Recruitment	NA
Ethics oversight	NA

Note that full information on the approval of the study protocol must also be provided in the manuscript.

Field-specific reporting

Please select the one below that is the best fit for your research. If you are not sure, read the appropriate sections before making your selection.

☒ Life sciences ☐ Behavioural & social sciences ☐ Ecological, evolutionary & environmental sciences

For a reference copy of the document with all sections, see nature.com/documents/nr-reporting-summary-flat.pdf

Life sciences study design

All studies must disclose on these points even when the disclosure is negative.

Sample size	information on sample sizes is provided in the Methods and the Supplement
Data exclusions	information on data exclusions is provided in the Methods and the Supplement
Replication	NA as these are not experimental multiplets

Randomization	used where appropriate; information on randomization is provided in the Methods and the Supplement
Blinding	used where appropriate; information on blinding is provided in the Methods and the Supplement

Reporting for specific materials, systems and methods

We require information from authors about some types of materials, experimental systems and methods used in many studies. Here, indicate whether each material, system or method listed is relevant to your study. If you are not sure if a list item applies to your research, read the appropriate section before selecting a response.

Materials & experimental systems

n/a	Involved in the study
☒	☐ Antibodies
☒	☐ Eukaryotic cell lines
☒	☐ Palaeontology and archaeology
☒	☐ Animals and other organisms
☒	☐ Clinical data
☒	☐ Dual use research of concern

Methods

n/a	Involved in the study
☒	☐ ChIP-seq
☒	☐ Flow cytometry
☒	☐ MRI-based neuroimaging
