# Supplementary figures and images for: Sequence-structure-function relationships in the microbial protein universe
Source: Nat Commun. 2023 Apr 26;14:2351. doi: 10.1038/s41467-023-37896-w (PMC10133388; doi:10.1038/s41467-023-37896-w)

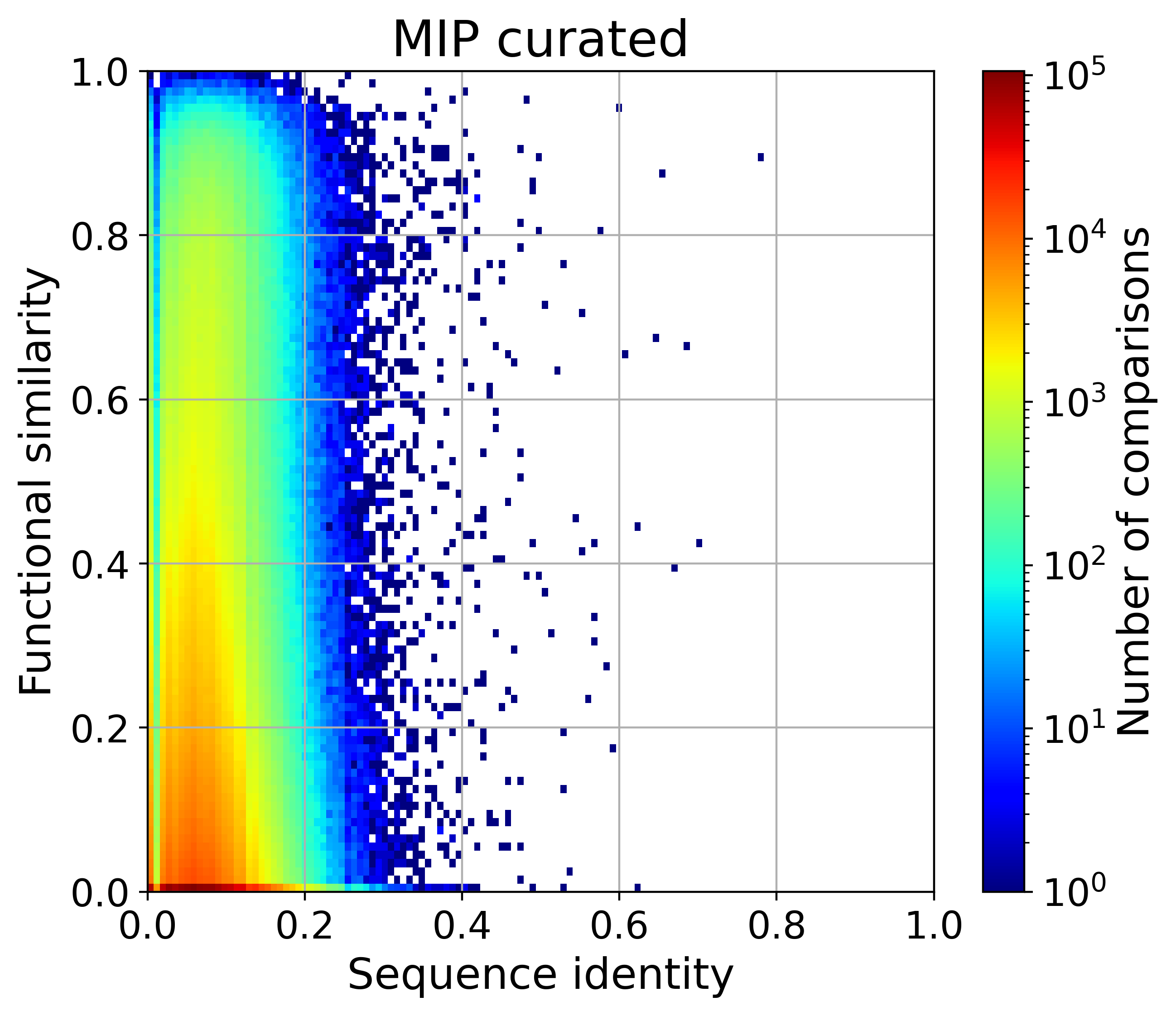

Supplement: Supplementary file 9 — Source Data [file 41467_2023_37896_MOESM9_ESM.zip › SourceData/source_data_Fig2/Figure_2_MIP-curated_Rosetta_sequence-identity_vs_functional-similarity.png]

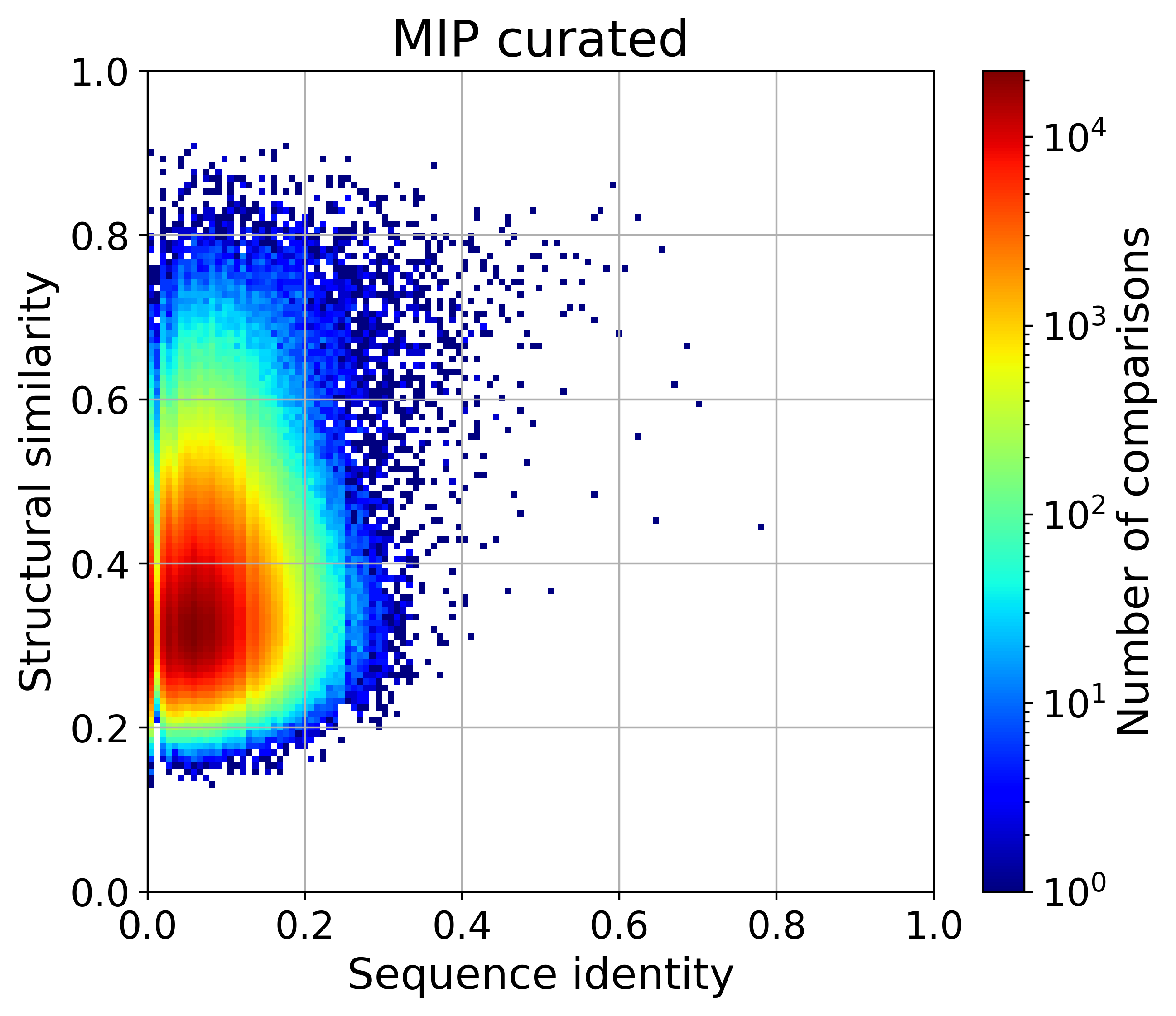

Supplement: Supplementary file 9 — Source Data [file 41467_2023_37896_MOESM9_ESM.zip › SourceData/source_data_Fig2/Figure_2_MIP-curated_Rosetta_sequence-identity_vs_structural-similarity.png]

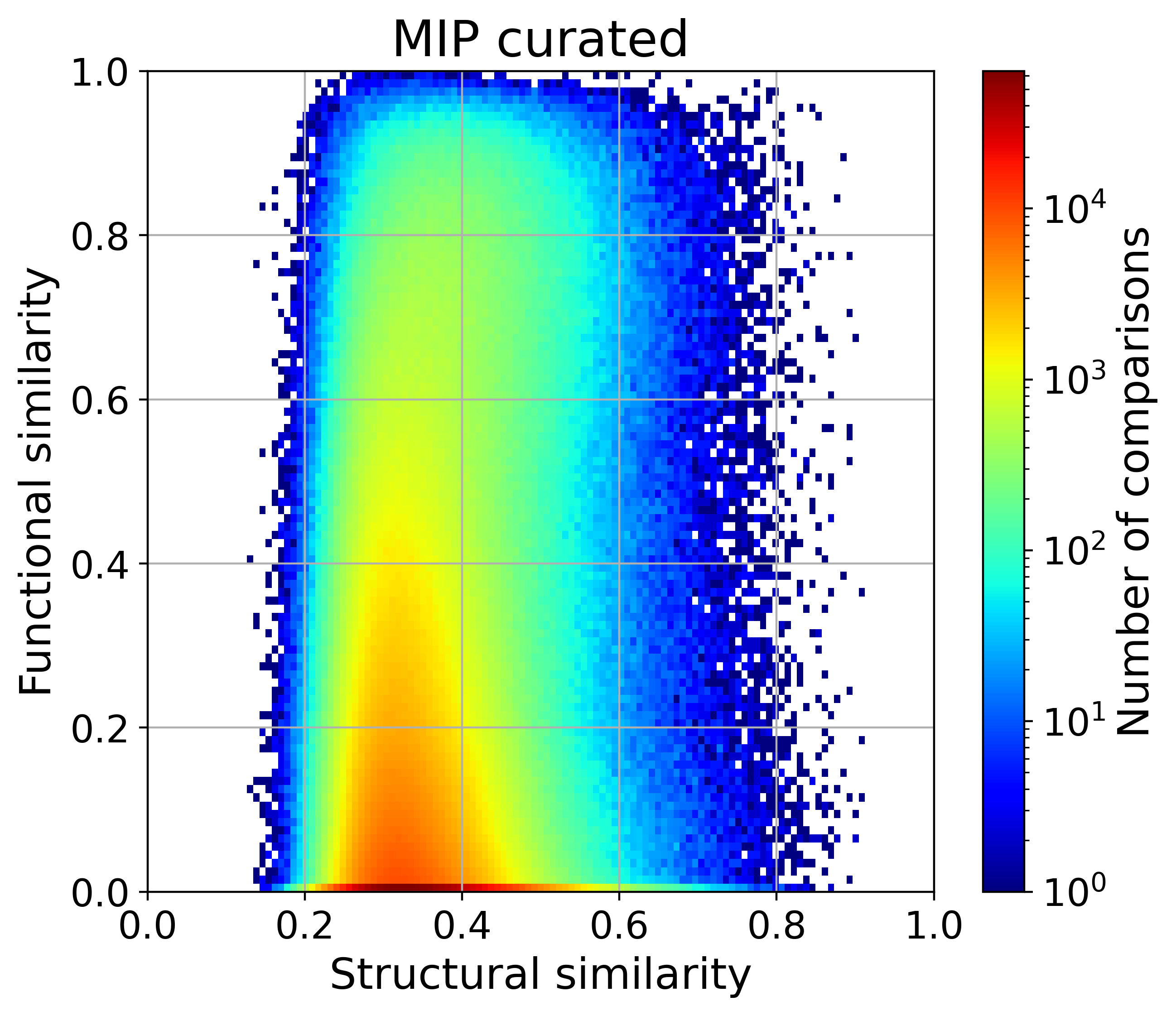

Supplement: Supplementary file 9 — Source Data [file 41467_2023_37896_MOESM9_ESM.zip › SourceData/source_data_Fig2/Figure_2_MIP-curated_Rosetta_structural-similarity_vs_functional-similarity.png]

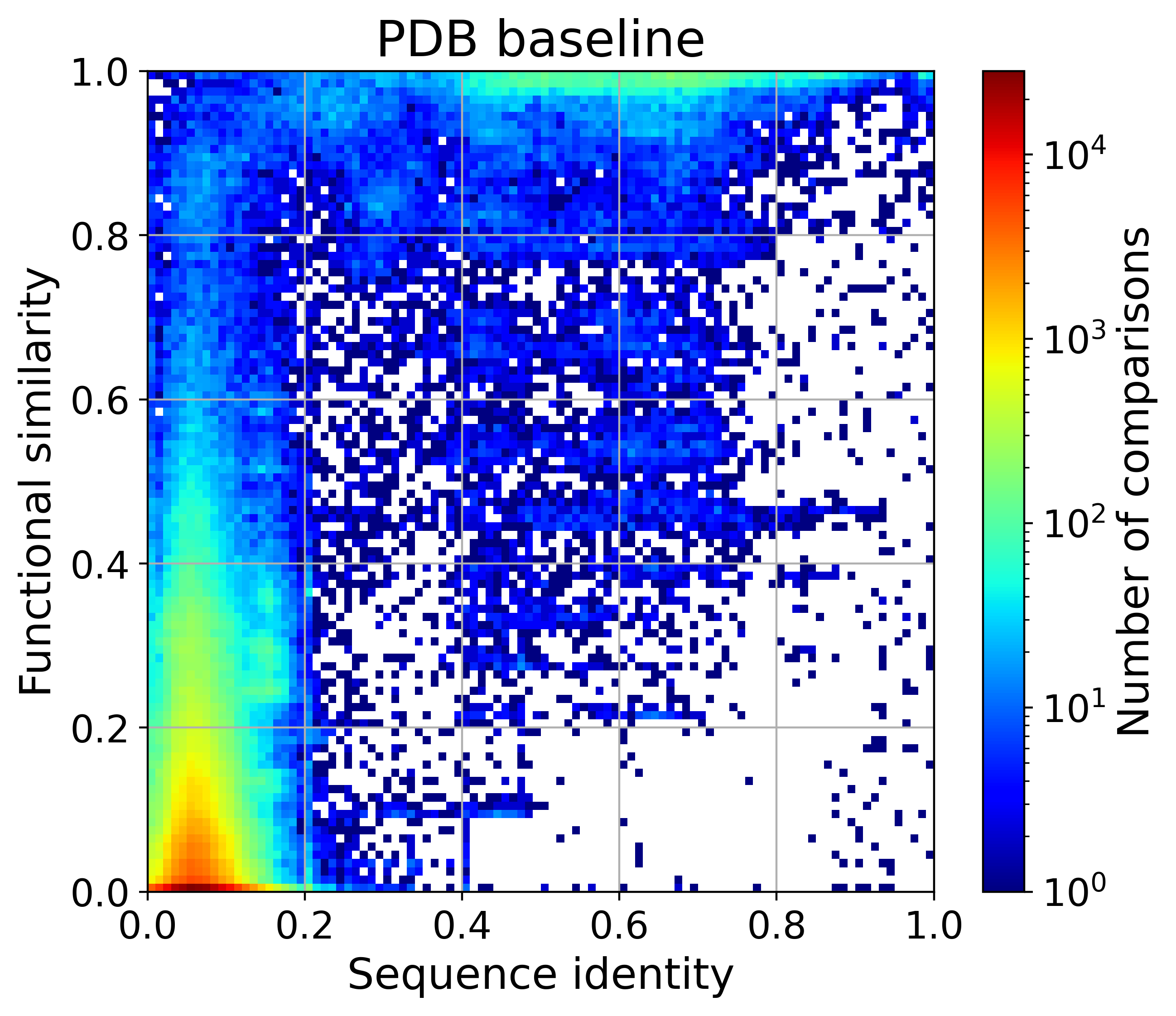

Supplement: Supplementary file 9 — Source Data [file 41467_2023_37896_MOESM9_ESM.zip › SourceData/source_data_Fig2/Figure_2_PDB-baseline_sequence-identity_vs_functional-similarity.png]

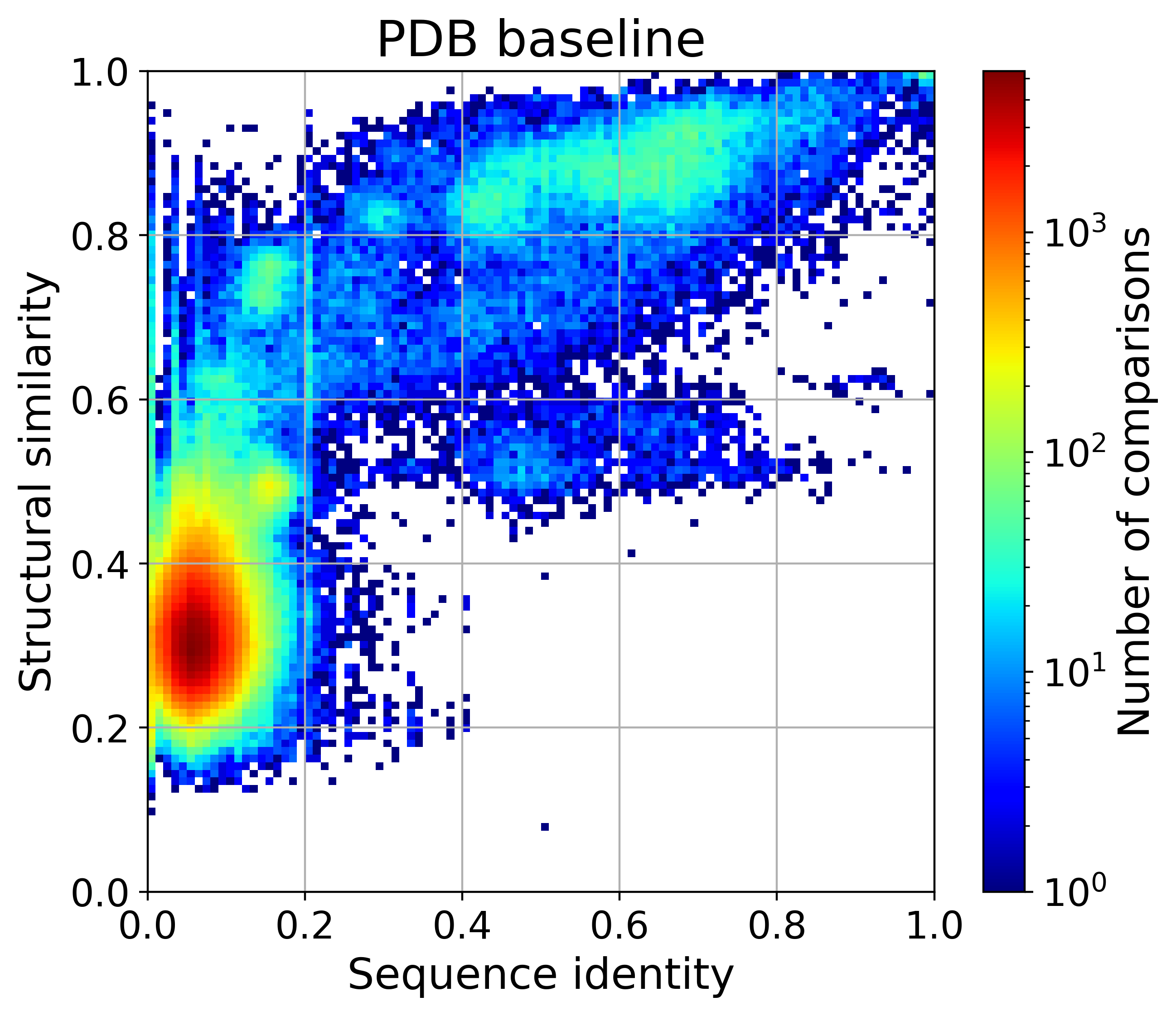

Supplement: Supplementary file 9 — Source Data [file 41467_2023_37896_MOESM9_ESM.zip › SourceData/source_data_Fig2/Figure_2_PDB-baseline_sequence-identity_vs_structural-similarity.png]

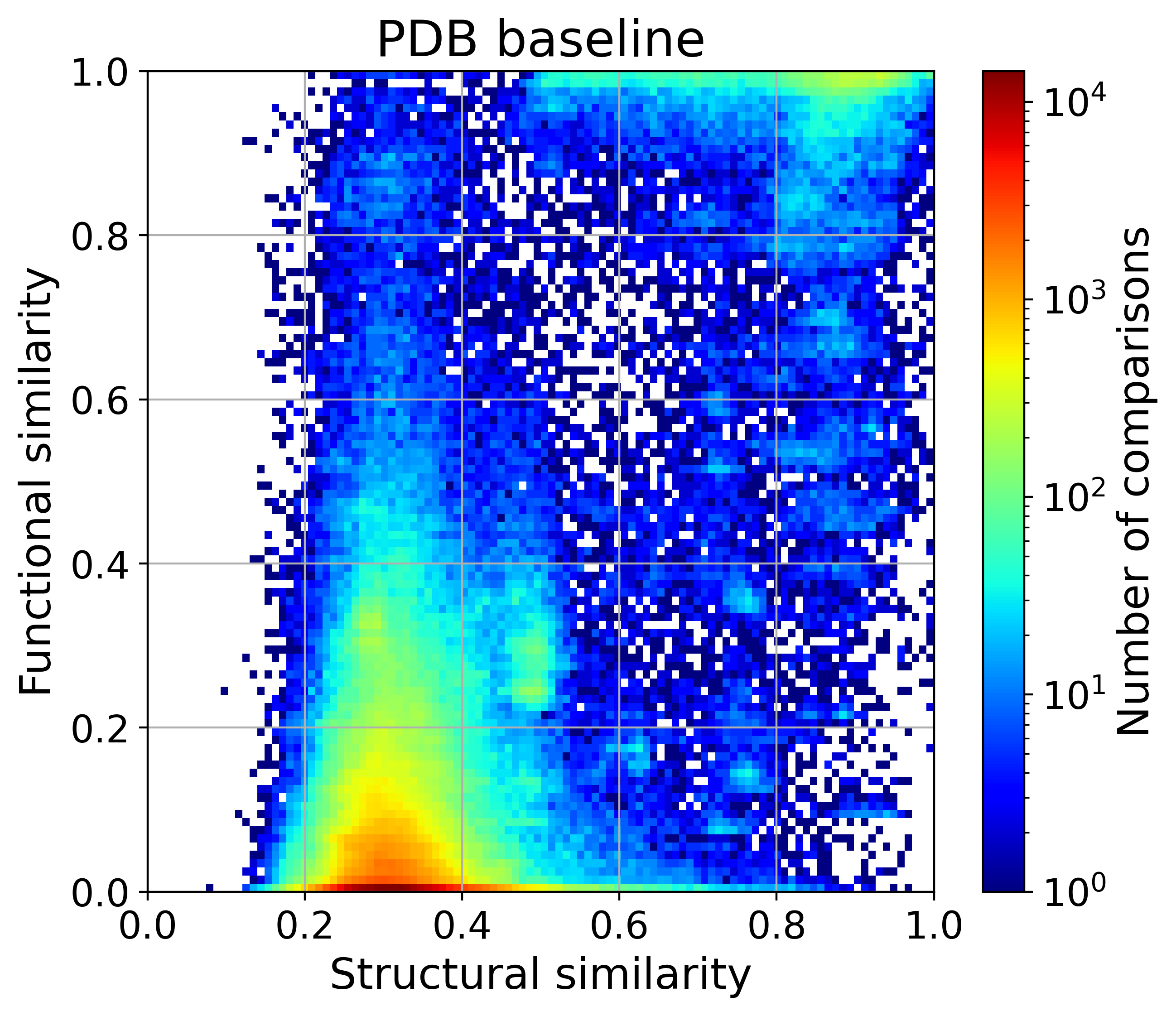

Supplement: Supplementary file 9 — Source Data [file 41467_2023_37896_MOESM9_ESM.zip › SourceData/source_data_Fig2/Figure_2_PDB-baseline_structural-similarity_vs_functional-similarity.png]

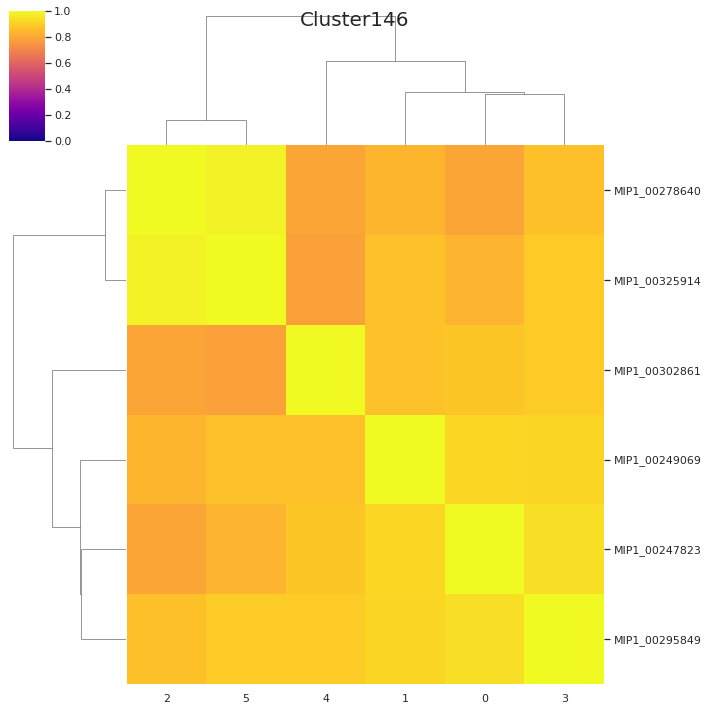

Supplement: Supplementary file 9 — Source Data [file 41467_2023_37896_MOESM9_ESM.zip › SourceData/source_data_Fig3/Figure_3_cluster=146.png]

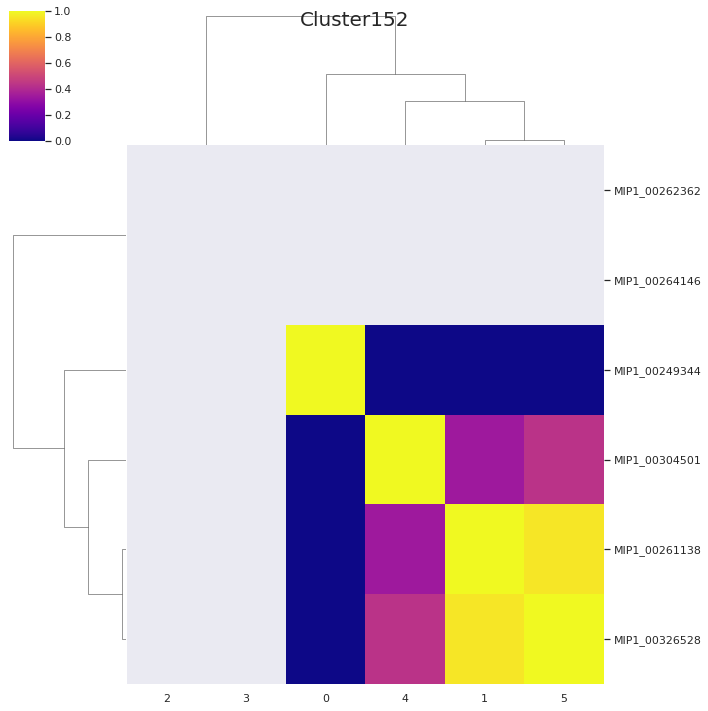

Supplement: Supplementary file 9 — Source Data [file 41467_2023_37896_MOESM9_ESM.zip › SourceData/source_data_Fig3/Figure_3_cluster=152.png]

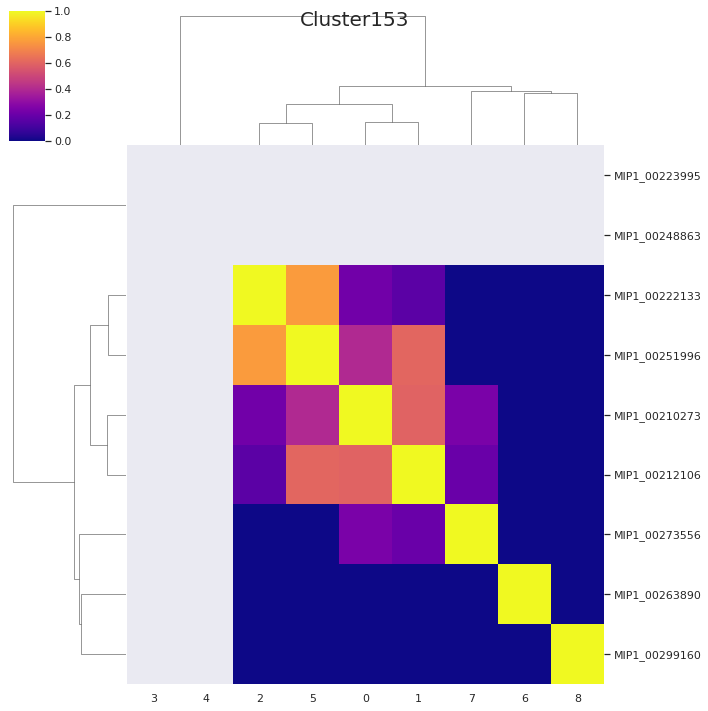

Supplement: Supplementary file 9 — Source Data [file 41467_2023_37896_MOESM9_ESM.zip › SourceData/source_data_Fig3/Figure_3_cluster=153.png]

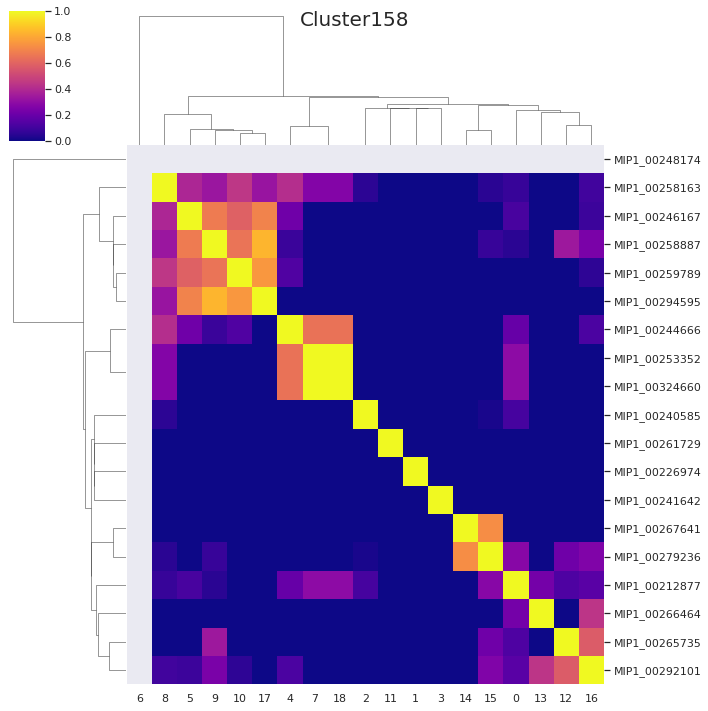

Supplement: Supplementary file 9 — Source Data [file 41467_2023_37896_MOESM9_ESM.zip › SourceData/source_data_Fig3/Figure_3_cluster=158.png]

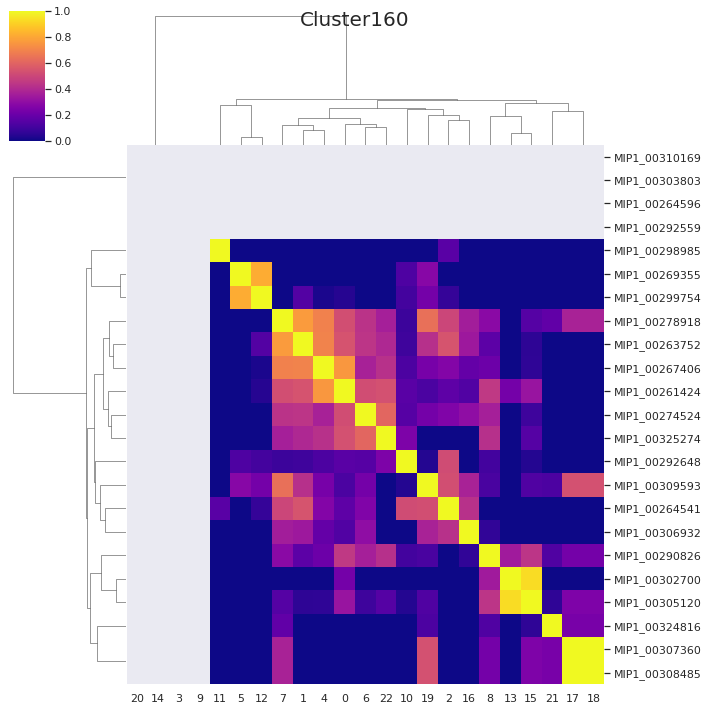

Supplement: Supplementary file 9 — Source Data [file 41467_2023_37896_MOESM9_ESM.zip › SourceData/source_data_Fig3/Figure_3_cluster=160.png]

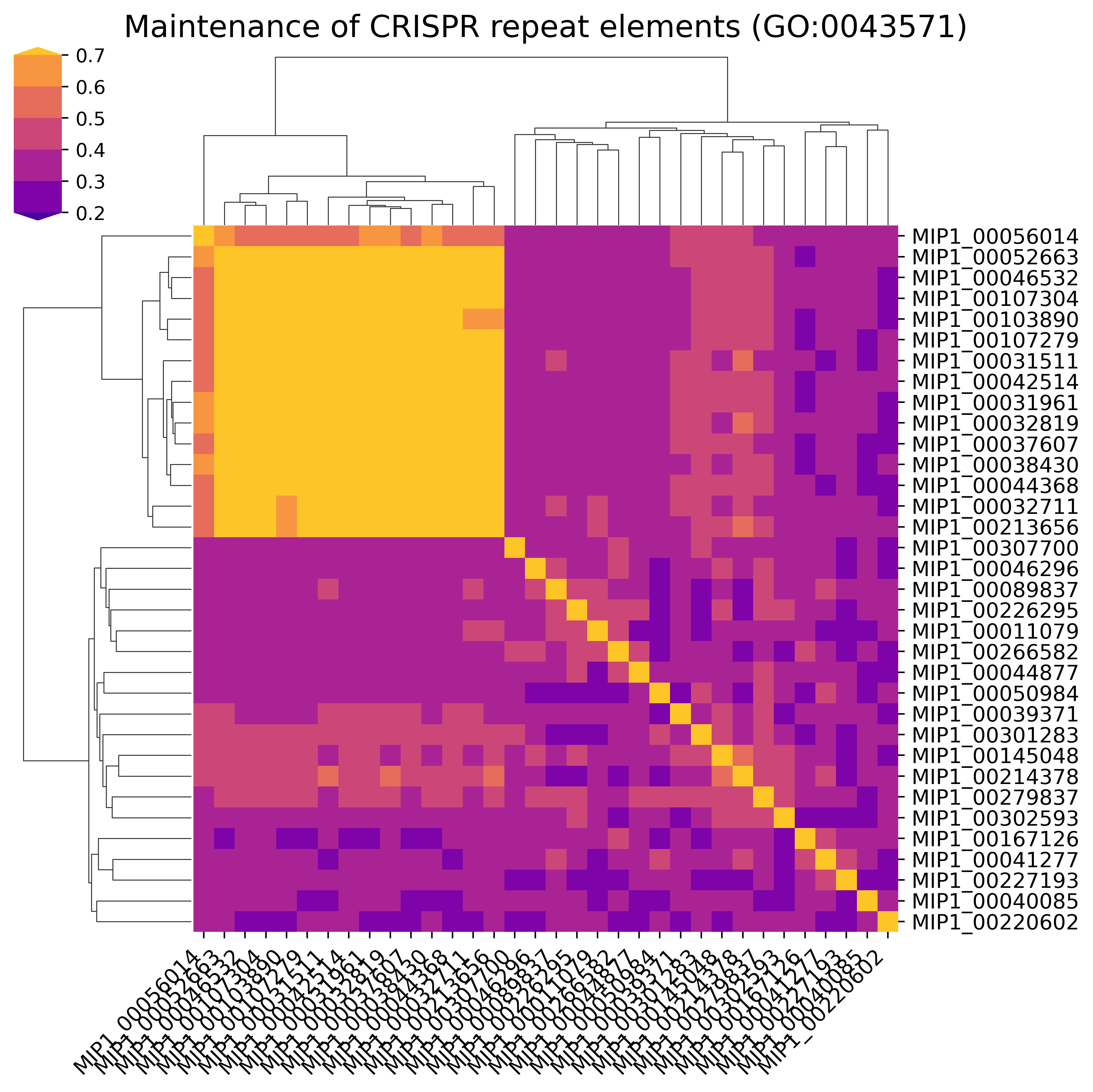

Supplement: Supplementary file 9 — Source Data [file 41467_2023_37896_MOESM9_ESM.zip › SourceData/source_data_Fig4/Figure_4_Rosetta_BP_GO_0043571.png]

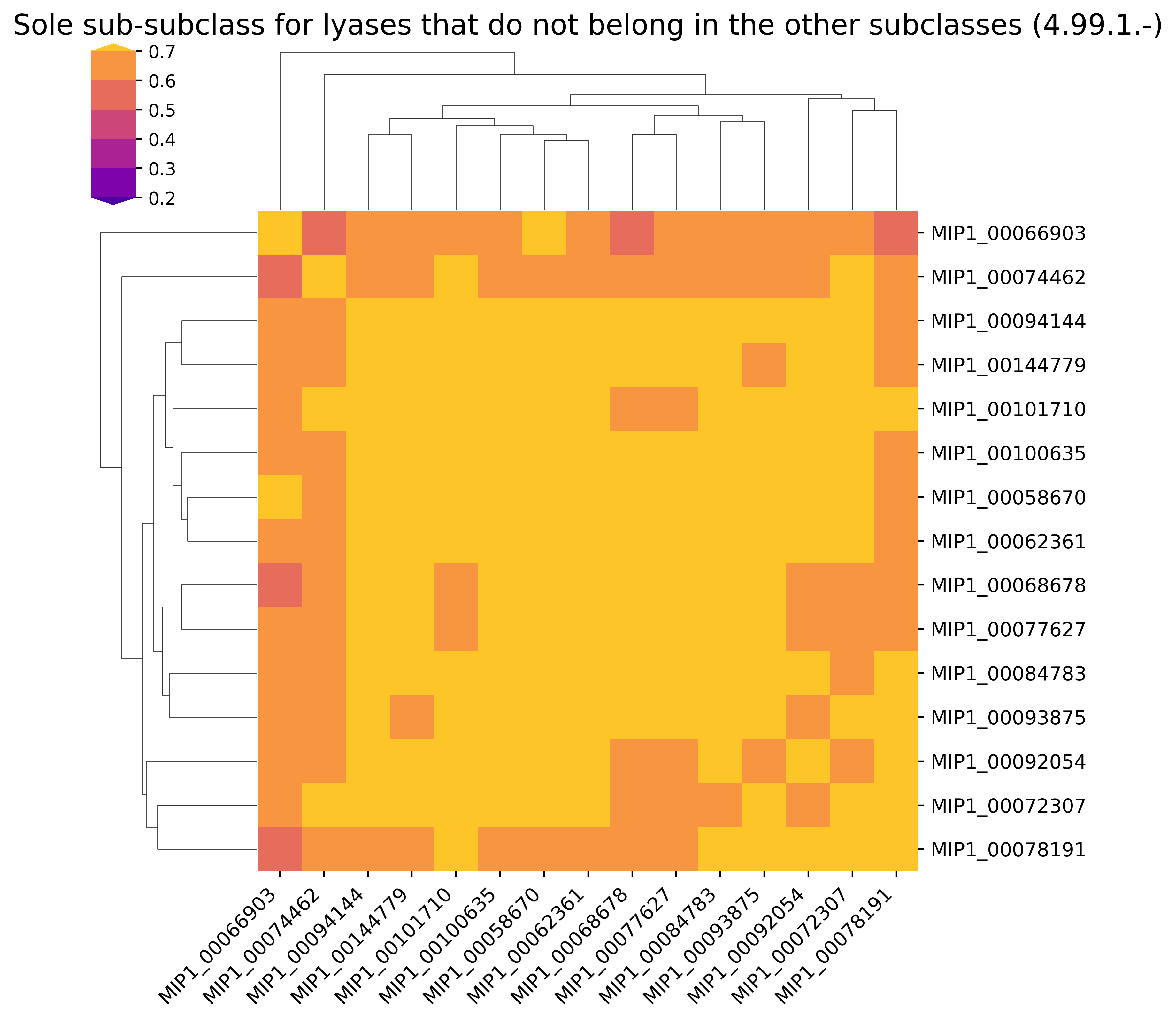

Supplement: Supplementary file 9 — Source Data [file 41467_2023_37896_MOESM9_ESM.zip › SourceData/source_data_Fig4/Figure_4_Rosetta_EC_4.99.1.-.png]

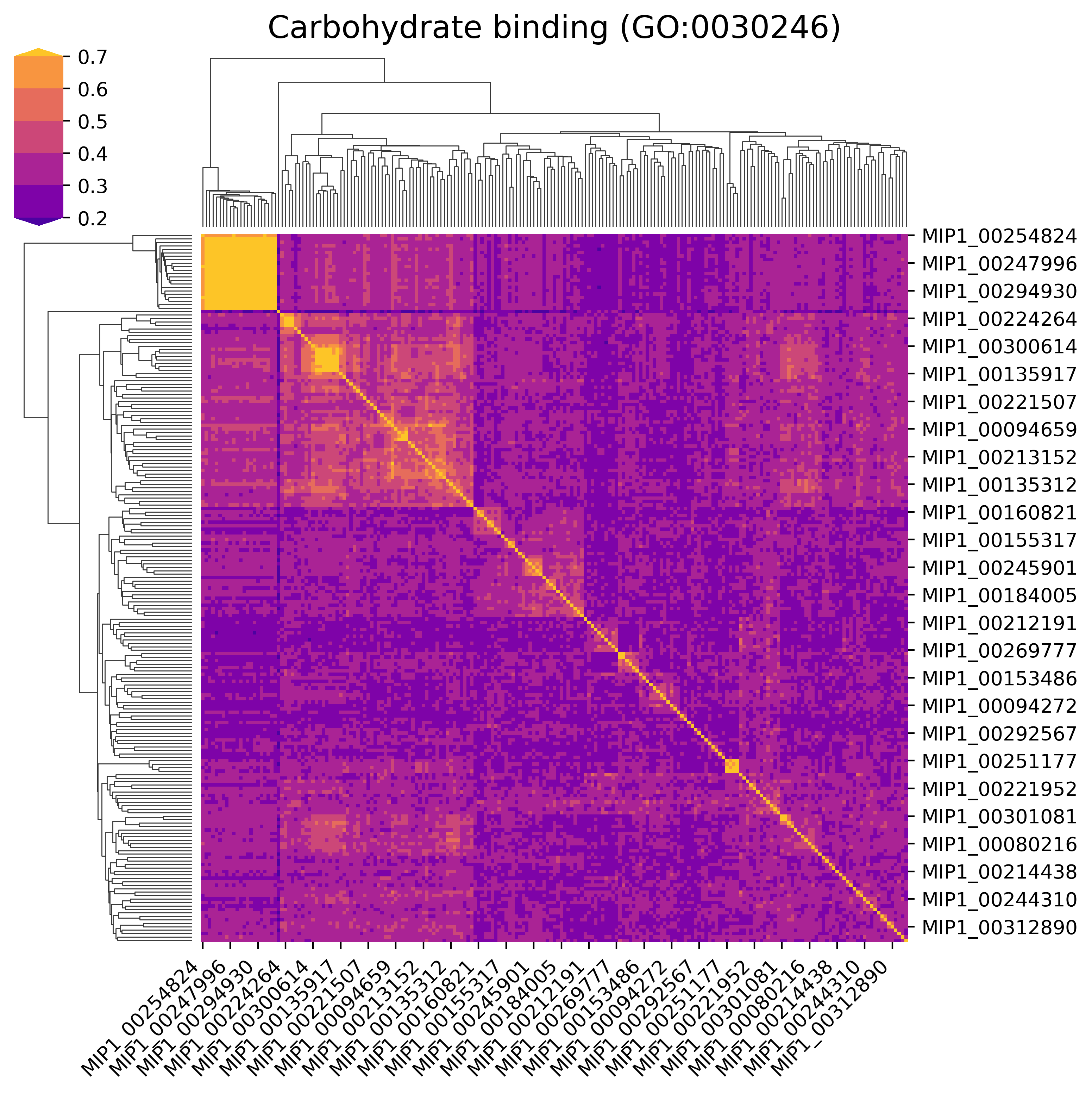

Supplement: Supplementary file 9 — Source Data [file 41467_2023_37896_MOESM9_ESM.zip › SourceData/source_data_Fig4/Figure_4_Rosetta_MF_GO_0030246.png]
